# Examining subjective views of the aging process in older adults: a systematic review following the COSMIN methodology

**DOI:** 10.1186/s12877-025-06472-w

**Published:** 2025-11-13

**Authors:** Enrico Sella, Elena Carbone, Riccardo Domenicucci, Maria Letizia Tanturri, Antonio Paoli, Erika Borella

**Affiliations:** 1 Department of General Psychology, University of Padova, Via Venezia, 8, Padova, 35131 Italy; 2 Department of Statistical Sciences, University of Padova, Via Cesare Battisti, 241/243, Padova, 35123 Italy; 3 Department of Biomedical Sciences, University of Padova, Via Ugo Bassi 58/B, Padova, 35131 Italy

**Keywords:** Views of Aging, Awareness of Aging, Self-reported measures, Systematic review

## Abstract

**Background:**

This systematic review is intended to identify, categorize, and evaluate instruments assessing generalized and personal views of aging (VoA) in older adults as well as how these instruments have addressed views of changes in cognition with aging.

**Methods:**

The Preferred Reporting Items for Systematic Reviews and Meta-Analyses (PRISMA) guidelines were followed, and the review was preregistered in PROSPERO (CRD42023407986). Studies presenting VoA instruments, also including a specific focus on views of changes in cognition with aging, were identified using the Scopus, PsycINFO, and Web of Science databases. Based on the Consensus-based Standards for the Selection of Health Measurement Instruments (COSMIN) guidelines, risk of bias and GRADE assessments were conducted for each instrument.

**Results:**

Of the 3,578 records emerged, 77 studies assessing measurement properties of 26 VoA instruments in samples of older adults were included. These instruments reflected diverse generalized and personal VoA concepts; few ones assessed or included items depicting views of changes in cognition with aging, with memory being the most frequently addressed cognitive domain.

**Conclusions:**

Notwithstanding psychometric and measurement issues, this review provides a comprehensive summary of existing instruments assessing subjective VoA, which have clinical and applied utility in promoting healthy aging, especially for older adults at risk of pathological aging. The development and validation of new VoA scales, particularly to assess views on age-related cognitive changes with aging, is warranted.

**Supplementary Information:**

The online version contains supplementary material available at 10.1186/s12877-025-06472-w.

## Introduction

Subjective views of aging (VoAs) encompass individuals’ perceptions, attitudes, and expectations related to old age and older adults in general and their own aging process [1, 2]. They are an integral part of adults’ experience of growing older, and their well-established link with health, well-being, and longevity outcomes (e.g., [3] for a meta-analysis) accounts for the renewed and increasing interest in this complex, multidimensional construct. According to a recent theoretical framework (see [4]), subjective VoA, in fact, is an umbrella term containing a variety of concepts depicting various aspects of a person’s aging experience, and related measures to examine them, which have been recently categorized in generalized and personal VoA (see [2]).

The generalized VoA subcategory encompasses old age stereotypes, namely socially shared beliefs about the aging process [5] and about older adults as a social group in general, as well as attitudes toward aging, that is, expectations, experiences, and feelings regarding the aging process and old age. These are classical concepts that have been the focus of research in the VoA field for several years. Therefore, a variety of self-report questionnaires or implicit association tests have been developed to capture old age stereotypes across various age populations (e.g. [6, 7]). A recent review by Ayalon et al. [8] revealed 11 instruments for the assessment of old age stereotypes. However, this review showed that none of these instruments demonstrated robust psychometric properties (such as content validity, structural validity, and internal consistency). Self-reported measures have been also developed for assessing attitudes toward aging (see [9] for a review), such as the Attitudes to Ageing Questionnaire (AAQ) [5], which were determined to have suitable measurement properties for older adults.

The concept of essentialist and nonessentialist beliefs of aging has been recently added [10, 11], which capture individuals’ views of aging either as a fixed, inevitable, and biologically determined (essential) or a malleable, modifiable, and flexible (nonessential) process [10]. To evaluate this new concept’s empirical usefulness, a questionnaire assessing (non)essentialist beliefs about aging has been recently proposed (see [10]).

The personal VoA subcategory, on the other hand, refers to individuals’ beliefs about their own aging process and the state of being old. This second category includes subjective or felt age (i.e., how old someone feels), representing a self-perception of one’s aging self (e.g., [1, 12]). Personal VoA also includes the Attitudes Toward Own Aging (ATOA) [13], which refers to individuals’ evaluations of their aging process (e.g., [1]). These two concepts have a long-lasting history of accounting for personal VoA. The former is usually operationalized with the approach of asking individuals how old they feel and calculating the discrepancy between an individual’s felt age and actual chronological age, and the well-known ATOA subscale of the Philadelphia Geriatric Center Morale Scale (PGCMS) [13] is a well-established questionnaire for addressing the latter. These concepts—and related measurements—capture nuances of personal VoA from a general, unidimensional perspective (e.g., [1]).

A more recent conceptualization anchored to the personal VoA subcategory involves the awareness of age-related change (AARC) construct. It emphasizes the need to depict the personal behavioral experiences that give rise to individuals’ self-perceptions and awareness of aging, recognizing the multidimensional nature of the aging process and hence the fact that adults may have different aging-related experiences in different life domains [14]. The AARC therefore captures an individual’s awareness that their behavior, level of performance, or ways of experiencing life in different domains of functioning (e.g., physical, cognitive, socio-emotional) have changed as a consequence of growing older (e.g., [14]). A self-reported questionnaire has been developed to assess this concept’s explanatory relevance (AARC 50- or 10-item version), which has shown good psychometric properties (see [15, 16]).

Despite the recent and fundamental conceptual organization of subjective VoA into generalized and personal VoA categories, a systematic review of the assessment characteristics of instruments addressing this theoretical reconceptualization of VoA has not been conducted.

Only Klusmann et al. [17] have provided a review of instruments assessing VoA but categorized them into dimensions that reflect common broad assumptions and conceptualizations of this construct (e.g., whether VoA represents the status of being old or the process of getting older, a state or a trait characteristic, a uni- versus a multidimensional construct, a subconscious or a conscious process). This review did not consider neither recent advances in the theoretical framework grounding the assessment of VoA (i.e. [4]), however, nor the methodological quality of the included instruments. In addition, there is a gap in identifying subjective VoA instruments for older adults. This underscores the need for a more comprehensive synthesis and categorization of VoA instruments that not only considers recent theoretical advancements but also evaluates their methodological quality, particularly with regard to instruments designed for older adults.

Furthermore, a significant gap persists in understanding whether and how these instruments address older adults’ experiences and expectations of their cognitive functioning with increasing age. It is well known that aging is characterized by changes in various cognitive domains [18]. Memory, in particular, is often identified as one of the cognitive abilities susceptible to age-related decline that older adults frequently perceive as changing and are concerned about (e.g., [19–21]). Older adults are known to hold more negative views of and beliefs about their cognitive abilities than their younger counterparts, are driven by negative socially shared stereotypes, and experience lower self-efficacy and control over their cognitive abilities with a subsequent drop in their cognitive performance in everyday functioning [21]. This underscores the importance of addressing, within the multidimensional assessment of VoA, older adults’ views of changes in the cognitive domain of functioning with aging.

This study was therefore intended to provide a more comprehensive synthesis of subjective VoA instruments developed to assess subjective VoA among typically aging older adults. Following the current conceptual framework (see [2]), subjective VoA were here categorized as (i) generalized VoA (old age stereotypes, attitudes toward aging, and (non)essentialist beliefs about aging) and (ii) personal VoA (subjective age, self-perceptions of aging, attitudes toward one’s aging, awareness of age-related change). We also aimed to ascertain, for the first time to our knowledge, whether subjective VoA instruments assess older adults’ views of changes in cognitive functioning with aging, identifying items or subscales in each instrument and classifying the cognitive domains targeted (e.g., memory, learning, attention, language).

In addition, following the Consensus-based Standards for the Selection of Health Measurement Instruments (COSMIN) methodology (see [22]), another aim was to evaluate the VoA instruments’ quality of measurement and psychometric properties, employing the GRADE assessment (see [23]).

For the identified valid and reliable instruments for older adults, we also considered all the validation studies on VoA instruments published in various countries. This was important given the increasing cross-cultural perspective of VoA and the growing publication of evidence across various countries.

## Method

### Study eligibility criteria

The present study was conducted following the guidelines of the Preferred Reporting Items for Systematic Reviews and Meta-Analyses (PRISMA) [24] and the Joanna Briggs Institute (JBI) for conducting a systematic review of relevant and reliable instruments and their evidence-based measurement properties. The review protocol (CRD42023407986) was preregistered in the PROSPERO International Prospective Register of Systematic Reviews [25]. The literature search criteria were established based on the existing self-reported instruments for assessing VoA among older adults. These criteria were defined to align with the PICO(S) components (population, instruments, construct, outcome, and study type) for systematic reviews of measurement properties, in accordance with the JBI guidelines [26]. See Table S1 (Supplemental Material) for inclusion and exclusion criteria.

### Search strategy

Three authors (ES, EC, EB) led the development of the search strategy. The search strategy combines search terms describing the population (“older adult*” OR “older people” OR “elderly”), constructs of interest (“cognitive concerns” OR “awareness of age-related cognitive change*” OR “cognitive perceptions” OR “aging concerns” OR “felt age” OR “aging awareness” OR “awareness of age-related change*” OR “subjective aging” OR “views on aging” OR “age perception” OR “self-perceptions of aging” OR “age-related belief*” OR “belief* about aging” OR “belief* on aging” OR “age stereotyp*” OR “aging stereotyp*” OR “ageism” OR “myth* of aging” OR “attitud* toward aging”), and types of instruments and outcomes (“Survey” OR “Scales” OR “Tools” OR “Measurement*” OR “Instrument*” OR “Assessment*” OR “Validity” OR “reliability” OR “consistency” OR “screening”). The comprehensive literature search for relevant peer-reviewed articles was undertaken in March 2023, and updated in February 2025, in three electronic databases: Scopus, PsycINFO, and Web of Science. To ensure comprehensiveness, a specific search for articles mentioning the identified scales (see Results’ section) was also conducted in selected databases to locate all validation studies evaluating the instruments’ psychometric and measurement properties. Then, a snowball search was conducted to identify additional records for full-text review using Google Scholar’s “cited by” functions for each of the articles included in the original search [27]. Therefore, any validation study that referenced the scales identified through our literature search was included in the study. Additionally, we made a note of any additional instruments that were identified during the screening process for eligibility. See Table S2 (Supplementary Material) for the complete search algorithm with the keywords for each database.

### Study selection

Eligible studies were identified and then deduplicated using Zotero [28]. After the literature search, two reviewers (ES, GS -a neuropsychologist expert in psychology of aging) independently screened the titles and abstracts of the articles retrieved for eligibility. If they disagreed, one other author (EB) was consulted to reach a final decision.

Due to the high heterogeneity of studies and measurement instruments related to the constructs of interest found during the literature search, the first author (ES) developed an algorithm in collaboration with the other authors (EC, EB) to identify eligible articles for review. This decision-making algorithm was shared with the second reviewer, who screened the articles during the identification phase (see Figure S1, Supplemental Material, for the full study eligibility process).

### Data synthesis

Data were extracted and charted using tailored adaptations of the JBI data extraction templates for systematic reviews of measurement properties. One review author (ES) conducted data extraction, and a second reviewer (GS) conducted spot checks for comprehensiveness and accuracy on the included studies. Any disagreements were resolved through discussion.

Data extracted from the included studies encompassed various aspects, including the characteristics of the sample, such as age, gender, and sample size. The assessed construct focused on domains related to subjective VoA and older adults’ views of age-related changes in cognition with aging. Information about the measurement instruments, including the number of items, subscales (if applicable), and response scale, was also collected. Furthermore, the extracted data included the instruments’ psychometric properties, such as validity, consistency, and reliability. If no data was available for particular measurements’ properties, that was also reported.

To summarize the reviewed studies’ characteristics, the authors (ES, EC, EB) first categorized them into two main types of outcomes, following the latest theoretical framework in this research field [2]: (a) generalized VoA and (b) personal VoA. In each category, the studies were further subcategorized based on the main VoA components targeted. In particular, generalized VoA included beliefs about cognitive changes with aging, old age stereotypes, and attitudes toward aging. Personal VoA encompassed subjective age, self-perceptions of aging, attitudes toward one’s aging, and awareness of age-related change (see [2] for more details).

Finally, three expert psychologists specialized in the psychology of aging and age-related changes in cognition (MV, JCM, VV) were involved in identifying the items whose content encompassed VoA concepts related to cognitive functioning and age-related cognitive changes in aging. The authors (ES, EB, EC) then categorized the selected items into distinct cognitive domains of interest.

### Methodological quality assessment (risk of bias)

The included studies’ methodological quality was assessed using the risk of bias tool from the COSMIN guidelines [22, 23]. Nine psychometric properties were evaluated for each scale: content validity, structural validity, internal consistency, cross-cultural validity, reliability, measurement error, criterion validity, construct validity, and responsiveness. The COSMIN guidelines were employed to assess each scale’s measurement properties, categorizing them as adequate (+), inadequate (-), inconsistent (±), or indeterminate (?). Additionally, the methodological quality of each measurement property per study was evaluated as very good, adequate, doubtful, or inadequate utilizing the COSMIN criteria [29]. Two raters (ES, RD) independently assessed the risk of bias for each psychometric property in each study. Any disagreements were resolved through consensus with a third reviewer (EB).

## Results

### Search results

A total of 3,578 records were initially retrieved through the literature search, and after duplicates were removed, 2,467 records remained. Screening the titles and abstracts resulted in the identification of 149 eligible reports. The level of agreement between the reviewers was assessed using Cohen’s kappa, and the calculated value of 0.77 indicated substantial and good agreement [30].

**Fig. 1:**
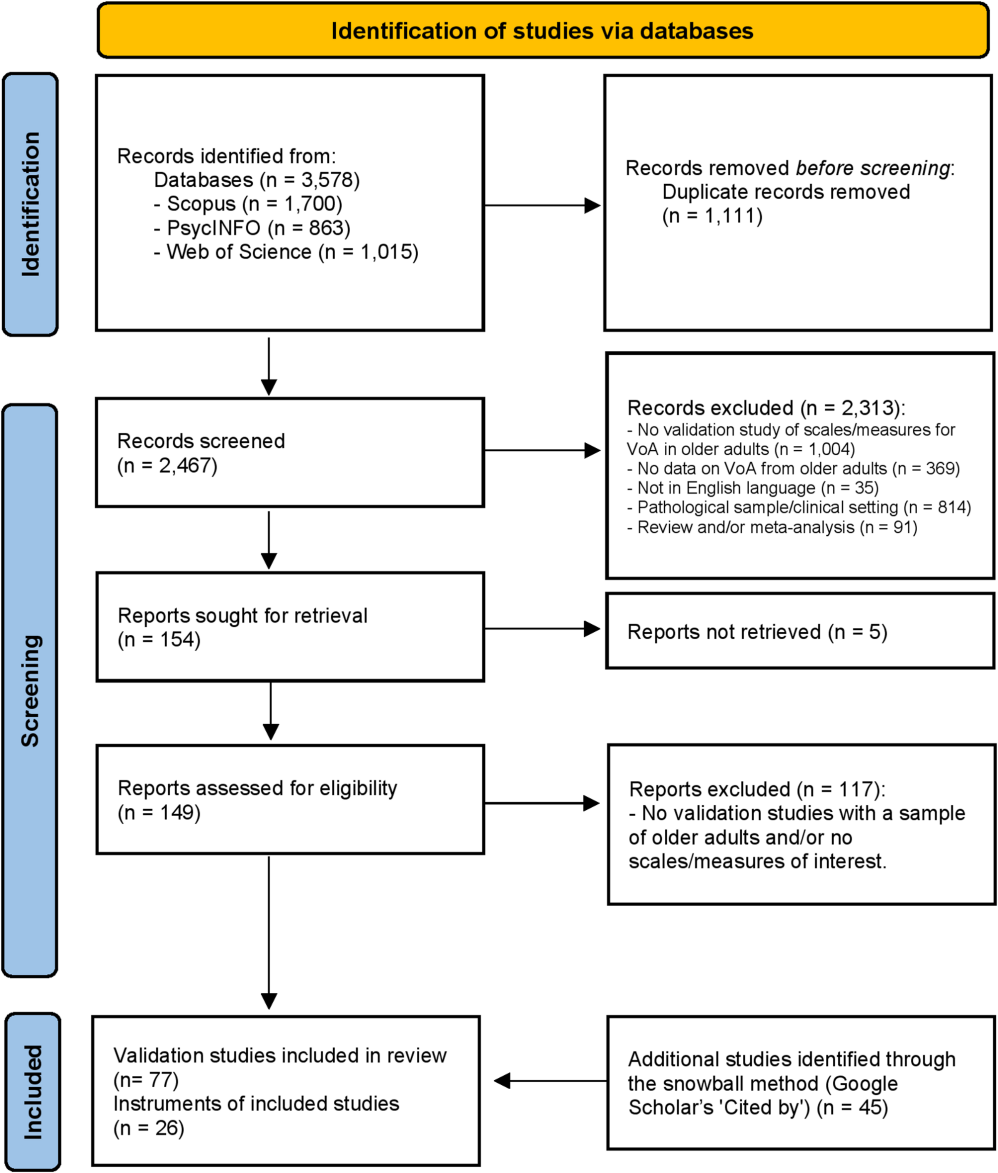
PRISMA 2020 flow diagram of the study selection procedure

Of the included reports, 23 were validation studies involving 10 self-reported instruments. Additionally, 55 reports were screened because they featured instruments relevant to the construct of interest even though they were not validation studies. From these, another 15 instruments were identified. Subsequently, 45 studies assessing measurement properties were included from the snowball search (see Methods section).^1^ In total, the current review included 26 instruments that assessed subjective VoA and older adults’ views of changes in cognition with aging, and 77 studies assessing their measurement properties (Fig. 1).

### Description of included studies

Table S3 provides details of the 77 studies conducted to examine the measurement properties of the instruments identified and included in the review. All the validation studies reviewed included a sample of community-dwelling older adults for whom the measures were developed. The participants across these studies consisted of 75,490 adults and older adults. The studies were conducted in various countries worldwide. There were 23 studies in the North America (21 in United States), 3 in South America, 22 in Europe, 17 in Asia, 1 in Australia, and 1 in Africa. Additionally, 10 studies involved multicenter nations. Most of these studies (72) employed a cross-sectional design, and 5 employed a longitudinal design.

### Review findings (characteristics of the instruments)

The included instruments focused on various aspects of subjective VoA, including questionnaires and ad hoc single-item measures, categorized into generalized and personal VoA. Table 1 presents detailed information about each study category, and Table S3 (Supplementary Material) provides specific measurement characteristics from the validation studies.

**Table 1:** Synthesis of the measurement instruments and validation studies grouped by domains of subjective VoA

Subjective VoA	Constructs	Instrument	Instrument characteristics	No. of validation studies [References]
Generalized VoA	Beliefs about aging (and cognitive changes)	Active Ageing Awareness Questionnaire (AAAQ)	*Items:* 14 items and 2 standalone questions *Dimension(s):* total score and 2 subscales (health, non-health construct) *Response scale:* 4-point Likert scale (from “strongly disagree” to “strongly agree”)	1 [31]
		Knowledge of Memory Aging Questionnaire (KMAQ)	*Items:* 28 *Dimension(s):* 2 subscales (normal memory aging, pathological memory aging) *Response scale*: dichotomous (true/false)	1 [32]
		Essentialist and (Non)essentialist beliefs about aging [(N)EBA]	*Items:* 10 or 4 *Dimension(s):* 2 subscales (essentialism, nonessentialism of aging) *Response scale:* 7-point Likert scale (from* “*do not agree” to “absolutely agree”)	10-item (N)EBA: 2 [10, 11] 4-item (N)EBA: 1 [11]
		Single-item scale for (Non)essentialist beliefs about aging [(N)EBA-SIS]	*Item:* 1 *Dimension(s):* unidimensional (total score) *Response scale:* response option from −50 (“Aging is genetically predetermined and immutable”) to 50 (“Aging is malleable and changeable”)	1 [33]
	Old age stereotypes	Ageism Survey (AS)	*Items:* 20 *Dimension(s):* unidimensional (total score) *Response scale:* 3-point Likert scale (“Never”, “Once”, “More than once”)	3 [34–36]
		Age-Based Rejection Sensitivity Questionnaire (RSQ-Age)	*Items:* 15 *Dimension(s):* unidimensional (total score) *Response scale:* 6-point Likert scale (from “very unconcerned” to “very concerned”)	1 [37]
		Everyday Ageism Scale (EAS)	*Items:* 10 *Dimension(s):* total score and 3 subscales (exposure to ageist messages, ageism in interpersonal interactions, internalized ageism) *Response scale:* 4-point Likert scale (items 1–7: from “never” to “often”; items 8–10: from “strongly disagree” to “strongly agree”)	1 [38]
		Expectations Regarding Aging (ERA)	*Items:* 38 *Dimension(s):* total score and 10 subscales (general health, cognitive function, mental health, functional independence, sexual function, pain, sleep, fatigue, urinary incontinence, appearance) *Response scale:* 4-point Likert scale (from “definitely false” to “definitely true”)	ERA: 1 [39] 12-item ERA: 3 [40–42]
		Image of Aging Scale (IAS)	*Items:* 18 *Dimension(s):* 2 subscales (positive age-stereotype component, negative age-stereotype component) *Response scale:* 6-point Likert scale (from “strongly disagree” to “strongly agree”)	IAS: 1 [43] 14-item IAS: 1 [44]
		Multidimensional Scale for the Assessment of the Salience of Age (MSASA)	*Items:* 24 *Dimension(s):* 5 subscales (developmental gains and chances for development, developmental losses and risks of development, older people as a burden on society, age salience) *Response scale:* 4-point Likert scale (from “completely disagree” to “completely agree”)	1 [45]
		Relating to Old People Evaluation (ROPE)	*Items:* 20 *Dimension(s):* total score and 2 subscales (positive types of ageism, negative types of ageism) *Response scale:* 3-point Likert scale (never, sometimes, often)	1 [46]
		Views on Aging Scales (VAS)	*Items:* 27 bipolar statements *Dimension(s):* 8 subscales (family and partnership, friends and acquaintances, religion and spirituality, leisure activities and social or civic commitment, personality and way of living, financial situation and dealing with money-related issues, work and employment, physical and mental fitness, health, and appearance) *Response scale:* 8-point bipolar scale (choosing between opposite poles of a stereotypical belief about age)	2 [47, 48]
	Attitudes toward aging	Attitudes to Ageing Questionnaire (AAQ)	*Items:* 24 *Dimension(s):* 3 subscales (psychological growth, psychosocial loss, physical change) *Response scale:* 5-point Likert scale (from “not all true” to “extremely true”)	AAQ: 6 [5, 49–53] 12-AAQ: 3 [54–56] 19-AAQ: 1 [57] 15-AAQ: 1 [58] 22-AAQ: 2 [59, 60]
Personal VoA	Subjective age	Felt age	*Item:* 1 *Dimension(s):* unidimensional (years) *Response scale:* numerical rating scale	1 [61]
		Perceptions of age	*Items:* 4 single questions *Dimension(s):* 4 subscales (Age-group identity; Comparative age; Felt age; Subjective nearness-to-death) *Response scale:* Age-group identity (ranging from “teenager” to “very old”); Comparative age (from “much younger” to “older”); Felt age (years); Subjective nearness-to-death (years)	1 [62]
	Self-Perceptions of aging (and cognitive changes)	Anxiety about Aging Scale (AAS)	*Items:* 20 *Dimension(s):* total score and 4 subscales (fear of old people, psychological concerns, physical appearance, fear of loss) *Response scale:* 5-point Likert scale (from “strongly disagree” to “strongly agree”)	3 [63–65] 12-AAS: 3 [35, 66, 67]
		Aging-Related Cognitions Scales (AgeCog)	*Items:* 12 *Dimension(s):* 3 subscales (physical decline, continuous growth, social loss) *Response scale:* 4-point Likert scale (from “completely true” to “completely not true”)	2 [68, 69]
		Aging Perceptions Questionnaire (APQ)	*Items:* 32 *Dimension(s):* 7 subscales (timeline chronic, timeline cyclical, consequences positive, consequences negative, control positive, control negative, emotional representations) *Response scale:* 5-point Likert scale (from “strongly disagree” to “strongly agree”)	APQ: 4 [70–73] Brief-APQ: 6 [74–79]
		Metamemory in Adulthood (MIA)	*Items:* 108 *Dimension(s):* 8 subscales (knowledge of memory strategies [strategy], knowledge of memory tasks and processes [task], knowledge of own memory capacities [capacity], attitudes toward own memory perception of change [change], activities supportive of memory [activity], memory and state anxiety [anxiety], memory and achievement motivation [achievement], locus of control in memory abilities [locus]) *Response scale:* 5-point Likert scale (from “agree strongly” to “disagree strongly”)	7 [80–86]
		Memory Controllability Inventory (MCI), Aging Concerns Scales (ACS)	*Items:* 12 (MCI) and 7 (ACS) *Dimension(s):* For MCI: 4 subscales (present ability, potential improvement, effort utility, inevitable decrement); For ACS: 2 sub-scales (independence, Alzheimer’s likelihood) *Response scale:* 7-point Likert scale (from “strongly disagree” to “strongly agree”)	1 [87]
		Memory Functioning Questionnaire (MFQ)	*Items:* 64 *Dimension(s):* 4 subscales (general frequency of forgetting, seriousness of forgetting, retrospective functioning, mnemonics usage) *Response scale:* 7-point Likert scale (ranging from “Always” to “Never” for frequency-related items, and “very serious/bad” to “not serious/very good” for severity-related items)	4 [88–91]
		Practical Memory Concerns Survey (PMCS)	*Items:* 7 open-ended questions *Dimension(s):* 4 subscales (memory self-efficacy, memory management, memory remediation, and memory fears in adulthood) *Response scale:* free-response question	1 [92]
		Subjective Aging Perception Scale (SAPS)	*Items:* 12 *Dimension(s):* 4 subscales (physical self-concept, cognitive self-concept, subjective perception of time, subjective perception of social relations) *Response scale:* 7-point Likert scale (from “totally disagree” to “totally agree”)	1 [93]
		Positive Aging Scale (PAS)	*Items: 8* *Dimension(s):* unidimensional (total score) *Response scale:* 6-point Likert scale (from “disagree strongly” to “agree strongly”)	1 [94]
	Awareness of age-related change	Awareness of Age-Related Change (AARC)	*Items:* 50 *Dimension(s):* 2 subscales and 5 domains each (AARC-Gains: health and physical functioning, cognitive functioning, interpersonal relations, social-cognitive and social-emotional functioning, lifestyle and engagement; AARC-Losses: health and physical functioning, cognitive functioning, interpersonal relations, social-cognitive and social-emotional functioning, lifestyle and engagement) *Response scale:* 5-point Likert scale (from “not at all” to “very much”)	50-item AARC: 2 [15, 95] 10-item AARC: 7 [95–101]
	Attitudes toward own aging	Attitudes Toward Own Aging Scale (ATOA)	*Items:* 5 items from the Philadelphia Geriatric Center Morale Scale *Dimension(s):* unidimensional (total score) *Response scale:* 4-point Likert scale (from “strongly disagree” to “strongly agree”)	3 [13, 65, 102]

For details on the characteristics of the validation studies and all versions of the included instruments see Supplementary Material

#### Instruments assessing generalized VoA

Thirteen self-report instruments focused on individuals’ perceptions, attitudes and expectations regarding old age and older adults in general as well as beliefs about cognitive functioning and age-related cognitive changes in aging. All these measures took the form of questionnaires. We organized them into three categories, each representing a distinct aspect of generalized VoA.

##### Beliefs about aging (and cognitive changes)

This subcategory includes instruments that assess beliefs and knowledge about the aging process as well as beliefs about the effects of aging on cognitive functioning. Four instruments were identified: the Active Aging Awareness Questionnaire (AAAQ) [31], the Knowledge of Memory Aging Questionnaire (KMAQ) [32], the Nonessentialist Beliefs about Aging scale [(N)EBA] [10], and the Single-item scale for (Non)essentialist beliefs about aging [(N)EBA-SIS] [33]. In the recent conceptualization [2], (N)EBA measured a standalone concept expressing (non)essentialist views of aging (i.e., mindset of aging), assessing general beliefs about the inevitability or malleability of age-related changes; therefore, we decided to include it in this broader subcategory.

All these instruments acknowledge the multidimensionality of VoA (as in [17]). These instruments vary in number of items, ranging from a single-item question [33], and 4-item and 10-item versions of the (N)EBA [10], up to 28 items of the KMAQ [32], with various response scales, including bipolar scales and Likert scales. All studies included at least one validation study for internal and structural validity, with the (N)EBA presenting three validation studies (see Table 1, and Supplementary Material).

##### Old age stereotypes

This subcategory includes eight instruments: the Views on Aging Scales (VAS) [47], the Age-Based Rejection Sensitivity (RSQ-Age) [37], the Multidimensional Scale for the Assessment of the Salience of Age (MSASA) [45], the Everyday Ageism Scale (EAS) [38], the Image of Aging Scale (IAS) [43], the Relating to Old People Evaluation (ROPE) [46], the Ageism Survey (AS) [34], and the original Expectations Regarding Aging (ERA) [39]. The ERA presented items with intermediate content regarding the general and personal categories considered here: some items reflected general expectations of older adults (e.g., “As people get older, they worry more”), and others involved more personal evaluations of being older (e.g., “I expect that as I get older, I will become more forgetful”). It is therefore conceived as a measure of attitudes to aging and expectations of one’s’ aging process [9]), but it was here considered a measure of old age stereotypes (in line with [8]).

Most of these instruments evaluated multidimensionality of VoA, containing from two (IAS, ROPE) to eight subscales (VAS). Only the AS assesses the ageism construct as a one-dimensional construct (one factor). The number of items in these instruments ranges from 10 (EAS) to 38 (ERA), and participants responded on a Likert scale ranging from 3 to 5. Validation studies were conducted on older populations: two for the IAS and the VAS and three for the AS. The ERA has an extended version with 38 items and a shortened 12-item version, each supported by multiple studies evaluating its psychometric properties (see Table 1 and Supplementary Material).

##### Attitudes toward aging

The third subcategory includes the AAQ [5]. This questionnaire measures older adults’ opinions of and feelings about events, issues, and experiences of gains and losses associated with aging. However, its items have a more intermediate content, lying between generalized attitudes toward aging (e.g., “Old age is a time of loneliness”) and reflections about one’s aging process (e.g., “My health is better than I expected for my age”). It employs a multidimensional approach (three domains: psychosocial loss, physical change, and psychological growth) with a 5-point Likert scale and has been validated in the older population, including a shortened 12-item version [54], and supported by nine studies examining its psychometric properties (see Table 1 and Supplementary Material).

#### Instruments assessing personal VoA

Thirteen instruments were classified as measures of personal VoA. These tools are designed to assess individuals’ perceptions and personal representations regarding their aging process and the state of being old as well as beliefs and attributions about their cognitive aging and the nature of age-related cognitive changes with aging, especially in the memory domain (see cf. [19]). This category includes single-item question for subjective age, and questionnaires for self-perceptions of aging, attitudes toward one’s own aging, and awareness of age-related change.

##### Subjective age

This subcategory refers to the approach of asking individuals how old they feel or how old they view themselves as a global evaluation of an individual’s perception of’their aging process. It is operationalized in terms of felt age (how old an individual feels), age identity (what age group reflects how an individual feels), comparative age (whether an individual feels younger, older, or the same compared to other people of same chronological age), and subjective proximity to death (what an individual believes remains of their life). These aspects were assessed using either a 4- or 7-point Likert scale [62] and an open-ended response for the number of years [61, 62] (see Table 1 and Supplementary Material).

##### Self-perceptions of aging (and cognitive changes)

This subcategory includes nine instruments designed to assess individuals’ personal experiences as they grow older and their perceived age-related cognitive changes. These instruments are the Aging Perceptions Questionnaire (APQ) [70], the Subjective Aging Perception Scale (SAPS) [93], the Anxiety about Aging Scale (AAS) [63], the Practical Memory Concerns Survey (PMCS) [92], the Aging-Related Cognitions Scales (AgeCog) [68, 69], and Positive Aging Scale (PAS) [94]. We also identified and included in this category other three instruments: the Metamemory in Adulthood questionnaire (MIA) [80], the Memory Functioning Questionnaire (MFQ) [88], and the Memory Controllability Inventory (MCI) [87]. These latter investigate older adults’ memory functioning as the expression of so-called metamemory processes related to knowledge and beliefs about memory functioning and its age-related changes.

In particular, the MFQ [88] encompasses several items that examine self-perceptions and awareness of everyday remembering and forgetting and concerns about forgetting experiences, perceived changes in ones’ memory skills over time, and the use of memory techniques to aid one’s’ memory performance. The MCI [87] focuses on the assessment of beliefs about one’s’ current memory capability and the ability to find ways to support it as well as beliefs about its controllability with age. The MIA [80] investigates several constructs related to general knowledge of basic memory processes, beliefs about one’s memory capacity, and perceptions of how emotional-motivational factors and aids use could affect it as well as the perceived sense of control over memory skills. It is worth mentioning that the MIA evaluates general knowledge and beliefs regarding memory functioning, more likely related to generalized, stereotypical VoA (“As people get older, they tend to forget where they put things more frequently”) and older adults’ self-perceptions of their memory functioning and its age-related changes (“The older I get, the harder it is to remember clearly”), whereas the other two instruments involve only personal reflections and evaluations of memory functioning and its changes.

All these instruments are in the form of questionnaires except for the PMCS, which is presented as a workbook. They employ a multidimensional approach, encompassing various dimensions (ranging from two to seven cognitive dimensions; see Table S3, Supplementary Material) to evaluate perceptions of the effects of aging, and their number of items ranges from eight (AgeCog) to 108 (MIA). The response scales typically consist of 4 to 7 points on the Likert scale. Multiple validation studies were conducted on samples of older adults, ranging from 1 to 7 studies (see Table 1 and Supplementary Material).

##### Awareness of age-related change

The 50-item AARC (AARC-50) [15] and its 10-item abbreviated version (AARC-10) [97] employed a multidimensional approach to assess individuals’ belief that their behavior, level of performance, or ways of experiencing their lives have changed as a consequence of them having grown older (increased chronological age). In the full 50-item and abbreviated 10-item versions, half of the items assess an individual’s awareness of age-related gains, and the other half assess an individual’s awareness of age-related losses in five domains of functioning (physical health and functioning, cognitive functioning, interpersonal relationships, social-cognitive and social-emotional functioning, and lifestyle and engagement). Recently, the subscale from the AARC-50 related to the awareness of age-related changes in the cognitive functioning domain was also used independently (see [16]). It consists of 10 items that keep the distinction in perceptions of age-related cognitive gains, including knowledge, wisdom, and reflexivity (e.g., “With my increasing age, I realize that I have more experience and knowledge to evaluate things and people”), and perceptions of age-related cognitive losses, including processing speed, memory, and mental capacity (e.g., “With my increasing age, I realize that my mental capacity is declining”). These instruments used a 5-point Likert scale. Six validation studies were conducted on the AARC (one on the AARC-50 and five on the AARC-10) (see Table 1 and Supplementary Material).

##### Attitudes toward own aging

This subcategory includes the ATOA, which is a 5-item subscale from the PGCMS [13], employing a dichotomous (“Yes” or “No”) response scale. The ATOA is a unidimensional scale that assesses individuals’ expectations, cognitive representations, and perceptions of their aging process. Nine studies have been conducted to assess the entire instrument’s measurement properties (see Table 1 and Supplementary Material).

### Synthesis of items from instruments assessing the generalized or personal views of age-related cognitive changes

Nine instruments included a total of 156 items related to cognitive changes with aging in older adults, which accounted for 25% of all items across 26 instruments.

#### Memory and learning

Eight instruments included items assessing age-related changes in memory and learning processes with aging, for a total of 150 (94%) of the selected items. These instruments were (1) the SAPS [93], with 2 items (e.g. “I remember the things that happened a long time ago with just as much clarity as those that have happened recently”); (2) the AgeCog scale [68], with 1 item capturing the belief that retaining the ability to learn new things is associated with aging (i.e., “Aging means to me that I retain the ability to learn new things”); (3) the MIA [80], encompassing 47 items reflecting metacognitive aspects related to memory functioning (e.g., “It is important to me to have a good memory”); (4) the 12-Item ERA [40], with 2 items reflecting expectations of increased forgetfulness with age (e.g., “I expect that as I get older I will become more forgetful”); (5) the AARC-50 [15], with 1 item (i.e., “ With my increasing age, I realize that I am more forgetful”); (6) the MCI & Aging Concerns Scales (ACS) [87], including 15 items assessing memory aspects, such as the ability to remember essential things (e.g., “I can remember the things I need to”); (7) the AAAQ [31], with 1 item (“Continue learning new things”); and (8) the MFQ [88], comprising 5 items evaluating self-perceived memory issues (e.g., “How would you rate your memory in terms of the kinds of problems that you have”).

#### Attention

Only the AARC-50 assesses attention, with 1 item (“With my increasing age, I realize that I have a harder time concentrating”).

#### Processing speed

Two instruments assess processing speed changes with aging: the AARC-50, with 1 item (“With my increasing age, I realize that I am slower in my thinking”), and the 12-ERA, with 1 item (“It is impossible to escape the mental slowness that happens with aging”).

#### General cognitive functioning

Three instruments assess cognitive functioning changes with aging from a broader perspective: the AARC-50, with 1 item (“With my increasing age, I realize that my mental capacity is declining”), the SAPS, with 1 item (“I think I have the same mental agility as before”), and the PAS with 1 item (“In life, I have enough mental stimulation”).

### Methodological quality of reviewed instruments

Table 2 presents a comprehensive evaluation of each scale’s measurement properties. Alongside this evaluation, the quality of evidence is assessed using the GRADE approach [29].

**Table 2:** Overall rating and grading the evidence of each measurement property for each scale across all vadidation study

		CONTENT VALIDITY				STRUCTURAL VALIDITY	INTERNAL CONSISTENCY	CROSS CULTURAL VALIDITY	MEASUREMENT INVARIANCE	RELIABILITY	MEASUREMENT ERROR	CRITERION VALIDITY	CONSTRUCT VALIDITY	RESPONSIVENESS
		OVERALL SCORE	RELEVANCE	COMPREHENSIVENESS	COMPREHENSIBILITY									
Generalized VoA														
AAAQ	Rating	-	-	-	-	+	+			-			+	
	GRADE	D	D	D	D	A	A			C			C	
AAQ	Rating	±	±	±	±	±	±	?	±	±			±	
	GRADE	B	B	B	B	B	B	D	D	D			B	
AS	Rating	±	±	-	±	?	+		?					
	GRADE	D	D	D	C	B	A		C					
EAS	Rating	±	±	±	+	+	+		+				-	
	GRADE	D	D	D	D	B	A		B				D	
ERA	Rating	±	±	±	+	±	±			?		?	?	
	GRADE	B	B	A	B	B	C			C		D	B	
IAS	Rating	±	+	-	±	+	-			?			?	
	GRADE	C	B	C	D	B	B			B			C	
KMAQ	Rating	±	±	-	±	+							?	
	GRADE	D	D	D	D	C							D	
(N)EBA	Rating	±	±	±	±	?	+		±				±	
	GRADE	D	D	D	D	C	A		C				C	
(N)EBA-SIS	Rating	±	±	?	?					±			±	
	GRADE	D	D	D	D					C			C	
MSASA	Rating	±	±	+	+	?	?			?			?	
	GRADE	D	D	D	D	C	A			D			D	
ROPE	Rating	±	±	-	±	?	+			?			?	
	GRADE	B	B	B	B	D	C			D			D	
RSQ-Age	Rating	±	±	±	±	?	+			?			+	
	GRADE	D	D	D	D	D	A			D			A	
VAS	Rating	±	±	±	±	?	±			?		±		
	GRADE	D	D	D	D	C	B			D		B		
Personal VoA														
AARC	Rating	±	±	±	±	+	+	?	+	?		±	±	
	GRADE	B	B	B	B	B	A	C	A	C		B	B	
AAS	Rating	±	±	±	±	±	±	?	±			?	?	
	GRADE	C	C	C	C	B	B	D	B			D	D	
AgeCog	Rating	±	±	-	±	?	+						?	
	GRADE	C	C	C	C	A	A						C	
ATOA	Rating	±	±	±	±	+	±		?	±			±	
	GRADE	D	D	C	D	A	B		B	C			B	
APQ	Rating	±	±	±	±	±	±		?	±		±	±	
	GRADE	C	C	C	C	C	B		D	B		B	B	
Felt age	Rating	-	-	-	-	?								
	GRADE	D	D	D	D	D								
MCI, ACS	Rating	±	±	±	±	+	-			?			?	
	GRADE	D	D	D	D	A	A			C			D	
MFQ	Rating	±	±	±	±	±	+		±	?			±	
	GRADE	C	C	C	C	B	A		A	D			B	
MIA	Rating	±	±	±	±	±	±	?	±	?			±	
	GRADE	D	D	D	D	C	B	D	C	D			C	
PAS	Rating	?	?	?	?	+	+		+				±	
	GRADE	B	B	B	B	A	A		A				A	
Perceptions of age	Rating	±	±	-	±	?								
	GRADE	D	D	D	D	D								
PMCS	Rating	±	±	-	±	?								
	GRADE	D	D	D	D	D								
SAPS	Rating	?	±	?	?	+	+						?	
	GRADE	C	C	C	C	A	A						D	

*Overall rating of psychometric property (Rating), *+ (sufficient), —(insufficient), ± (inconsistent), ? (indeterminate). *Overall quality of the evidence (GRADE),* A = High (very confident that the true measurement lies close to the estimate), B = Moderate (moderately confident in the measurement property estimate), C = Low (limited confidence in the measurement property). D = Very low (very little confidence in the measurement property estimate)

*Questionnaires: AAAQ * Active Ageing Awareness Questionnaire, *AARC * Awareness of Age-Related Change, *AAS * Anxiety about Aging Scale, *AAQ * Attitudes to Ageing Questionnaire, *AgeCog * Aging-Related Cognitions Scales, *APQ * Aging Perceptions Questionnaire, *ACS * Aging Concerns Scales, *AS * Ageism Survey, *EAS * Everyday Ageism Scale, *ERA * Expectations Regarding Aging, *IAS * Image of Aging Scale, *KMAQ* Knowledge of Memory Aging Questionnaire, *MCI * Memory Controllability Inventory, *MFQ * Memory Functioning Questionnaire, *MIA * Metamemory in Adulthood, *MSASA * Multidimensional Scale for the Assessment of the Salience of Age, (*N*)*EBA* Essentialist and (Non)essentialist beliefs about aging, (*N*)*EBA*-*SIS * Single-item scale for (Non)essentialist beliefs about aging, *PAS * Positive Aging Scale, *Perceptions of age* Age-group identity, Comparative age, Felt age, Subjective nearness-to-death, *PMCS * Practical Memory Concerns Survey, *ROPE* Relating to Old People Evaluation, *RSQ*-*Age * Age-Based Rejection Sensitivity Questionnaire, *SAPS * Subjective Aging Perception Scale, *VAS * Views on Aging Scales, *ATOA * Attitudes Toward Own Aging Scale

#### Content validity

Content validity was evaluated for all 26 instruments. Most instruments (22 out of 26) received inconsistent ratings, with 2 evaluated as insufficient and 2 as indeterminate. None of the instruments were evaluated as having sufficient/adequate properties. As for the GRADE, 15 instruments were downgraded to very low-quality ratings, 6 to low quality, and another 5 to moderate quality. None of the instruments achieved a high-quality rating.

When we examined the specific measurement properties of content validity (i.e., relevance, comprehensiveness, and comprehensibility), there was heterogeneity in the evaluations.

For relevance, 22 instruments were rated as inconsistent, 2 as insufficient, 1 as sufficient, and 1 as indeterminate. This divergence was also reflected in the GRADE assessment, in which 15 instruments received a very-low-quality grade, 5 were rated as low quality, and 6 were placed in the moderate-quality category. Then, regarding comprehensiveness, a similar pattern emerges, with 13 instruments evaluated as inconsistent, 9 as insufficient, 3 as indeterminate, and only 1 as sufficient. Ratings for 14 instruments were downgraded to very low quality, 7 to low quality, 4 to moderate quality, and only 1 to high quality. In addition, comprehensibility was assessed as inconsistent in 18 instruments, insufficient in 2, indeterminate in 3, and 3 as sufficient. For 15 instruments, the ratings were downgraded to very low quality, 6 to low quality, and 5 to moderate quality.

#### Structural validity

Structural validity was evaluated for 25 instruments. Of those, 10 were categorized as indeterminate, 6 as inconsistent, and 9 as sufficient. With the GRADE approach, 6 instruments achieved a high-quality rating, 8 moderate quality, 6 low quality, and 5 very low quality.

#### Internal consistency

Internal consistency was evaluated for 21 of the 26 instruments. Among them, 11 were determined to have sufficient internal consistency, 7 were considered inconsistent, 2 were considered insufficient, and 1 was considered indeterminate. Following the GRADE approach, 12 instruments were classified as high quality, 7 as moderate, 2 as low quality.

#### Measurement invariance/Cross cultural validity

Measurement invariance was evaluated for 12 instruments. Of those, 5 yielded inconsistent results, 3 were indeterminate, and 4 were sufficient. As for the GRADE assessment, 3 evaluations for studies focusing on measurement invariance were rated high, 3 moderate, 3 low, and 2 very low.

In studies addressing cross-cultural validity, all 4 instruments were rated as indeterminate. The GRADE approach rated 3 of them as very low and 1 low.

Although differences in measurement invariance and cross-cultural validity have been scarcely examined, in some studies, researchers have adapted instruments for use in various sociocultural contexts. For instance, the APQ has been culturally adapted in several countries, including Ireland [70], France [71], the Netherlands [72], China [73], Iran [74, 75], Turkey [76], Japan [77]. The 10-AARC was developed in Germany and the United States [97] and has been adapted to several cultural contexts: Brazil [98], UK [96], Norway [99], Burkina Faso [100], Turkey [95], and Taiwan [101]. Similarly, the MIA scale was initially developed and adapted in the USA [80, 81] and underwent cultural adaptation in South Africa [82] and the Netherlands [83]. The MFQ was created and adapted in the USA [88–90], and then adapted for use with a sample of older individuals in Italy [91]. The AAQ, initially developed in English in various centers [5], has been subsequently translated and culturally adapted in multiple regions/countries: Brazil [60], Canada [59] and Norway [49], Spain [55], France [50], Malaysia [51, 52], Scotland [54], Portugal [57], China [58], and Poland [56]. The AAS, originated in English in the USA [63], has been translated and adapted in other countries, including Australia [53], Iran [66], and Mexico [64]. Furthermore, the AS, initially developed and adapted in English in the USA [34], has been translated and culturally adapted in Turkey [35] and Iran [36]. Also, the NEBA was created and validated with samples of older individuals in the USA and Germany [10, 11].

#### Reliability

Reliability was assessed for 15 instruments. Of those, 10 demonstrated indeterminate results, 4 were inconsistent, and 1 was insufficient. As for the GRADE assessment, 7 instruments were evaluated as very low quality, 6 as low quality, and 2 as moderate quality.

#### Criterion validity

Criterion validity was assessed for only 5 instruments. Of those, 3 were inconsistent, and 2 were indeterminate. According to the GRADE approach, 2 instruments were rated as very low quality, and 3 as moderate quality.

#### Construct validity

Assessments of construct validity encompassed 21 instruments. Of those, 9 were evaluated as indeterminate, 9 as inconsistent, 2 as sufficient, and 1 as insufficient. Regarding the GRADE assessment, 7 instruments were rated as very low quality, 6 as low quality, 6 as moderate quality, and 2 as high quality.

In addition, it is noteworthy that none of the instruments evaluated measurement error or responsiveness. This absence underscores an evidence gap related to the instruments’ accuracy and sensitivity in tracking changes over time and detecting variations due to measurement imprecision.

## Discussion

This systematic review aimed to identify self-reported instruments measuring subjective VoA among older adults and to categorize them according to the recent theoretical distinction between generalized and personal VoA (see [2]). For the first time, how VoA instruments have addressed views on cognitive changes in aging was also examined. Following COSMIN guidelines, their measurement and psychometric properties were then assessed.

Our review included 26 self-report VoA instruments across 77 validation studies involving samples of older adults. These instruments were categorized as either generalized VoA or personal VoA [2]; within each category, further distinctions were made to capture their specific nuances. Generalized VoA encompassed “*old age stereotypes*”, “*attitudes toward aging*” and “*beliefs about aging*”, which also included beliefs about aging-related cognitive changes to specifically examine this domain. Personal VoA encompassed “*subjective age*”, “*attitudes toward one’s aging*”, “*awareness of age-related change*”, and “*self-perceptions of aging*”, with this latter construct also extended to age-related cognitive changes with aging.

In particular, as for generalized VoA, of the 12 questionnaires and 1 single-item-question identified, eight were included in the subcategory “*old age stereotypes*”, targeting socially shared beliefs and stereotypes about aging, including ageism scales. The AAQ was classified within the “*attitudes toward aging*” subcategory, reflecting opinions and feelings about aging and older people in general. The subcategory, labeled “*beliefs about aging (and cognitive changes)*”, included 3 instruments based on the concept of (non)essentialism of aging (i.e., beliefs about aging as an inevitable or malleable process [10]), such as the 10-item or 4-item (N)EBA, and its new single item question. This subcategory also encompassed instruments measuring beliefs/knowledge about awareness of the aging process (AAQ) and about age-related changes in memory/cognition (KMAQ).

Regarding personal VoA, we identified 11 questionnaires and two single-item questions, grouped into four subcategories. Two single-item questions targeted “*subjective age*”, evaluating general feelings of growing older through the discrepancy between individuals’ felt and chronological age. Eight instruments were included in the “*self-perceptions of aging (and cognitive changes)*” subcategory, assessing personal experiences of aging as well as age-related cognitive changes (APQ, SAPS, AAS, AgeCog, PAS), and older adults’ knowledge, evaluations, and expectations/concerns regarding memory functioning and its age-related changes (MCI, MFQ, MIA). These latter instruments allow examination of self-assessment of memory and beliefs about memory functioning, along with its regulation and controllability with aging. It is worth noting that these latter aspects are also part of the metacognitive processes related to memory, according to the metamemory framework, which emphasize the role of knowledge about how cognitive abilities and memory function, along with regulatory processes (monitoring and control skills), in supporting cognitive and memory performance in older adults.

The subcategory labeled “*awareness of age-related change*” included the 50- and 10-item versions of the AARC instrument, which employs a multidimensional, lifespan developmental perspective to assess individuals’ perceptions that their behavior, performance, or experiences have changed—both in terms of gains and losses—due to aging [103]. The “*attitudes toward own aging*” subcategory, included the ATOA from the PGMCS [13], which asks individuals to evaluate age-related changes and yields a general score indicative of personal self-perceptions of aging [13, 104].

All these personal VoA instruments thus reflect the dynamic and complex nature of personal VoA [1, 14, 105, 106], capturing how people perceive their age-related changes and the personal experiences of being older adults, whether through unidimensional representations (e.g. ATOA, felt age) or multidimensional ones (e.g. AARC).

A closer look at how VoA instruments specifically address older adults’ views of age-related cognitive changes with aging, as proposed here for the first time, highlights the need for a more comprehensive assessment of views of cognitive aging within the current VoA framework. Out of the twenty-six instruments included in our review, only eight assess older adults’ perceptions, experiences, and expectations regarding cognitive functioning and its age-related changes with aging. In particular, memory expectations (ERA) and attitudes toward aging related to memory (AAQ) were examined in two generalized VoA questionnaires, while only four personal VoA questionnaires included items targeting everyday memory challenges (AgeCog, SAPS), awareness of age-related changes in memory functioning (AARC), and the perception of one’s own mental abilities as sufficiently stimulated (PAS). Three other instruments (MFQ, MCI, MIA) specifically focused on self-assessed memory and metamemory functioning in older adults, whose impact on memory performance is well-documented [20, 107, 108]. It is also worth noting that the memory domain was overrepresented. Only two instruments included items assessing perceptions of general cognitive functioning (AARC, SAPS), and few instruments addressed other cognitive domains, such as attention (assessed only in the AARC) and processing speed (assessed in personal VoA with the AARC and general VoA with the ERA-12). Because age-related cognitive changes represent a prominent aspect of the aging process and could be perceived by older adults as more or less threatening, with subsequent consequences for their everyday functioning, future studies should also be devoted to capturing older adults’ views of how aging affects various cognitive domains.

Further, assessing older adults’ views on cognitive aging within the current VoA framework – which, as relieved by our synthesis, also includes metamemory aspects – can deepen our understanding of how generalized and personal beliefs about age-related memory changes relate to memory functioning among older adults.

As for the methodological quality and psychometric soundness, only half of the VoA instruments (13 out of 26, 50%) presented validation studies that thoroughly investigated their reliability and validity. Despite the availability of the scales, the psychometric properties of the instruments present several methodological weaknesses. Content validity was inadequately assessed, with only three generalized VoA instruments (ERA, AAQ, ROPE) and two personal VoA instruments (AARC, PAS) receiving a moderate quality rating. Construct validity had moderate-high quality ratings for five personal VoA instruments (AgeCog, PAS, AtoA, MCI, SAPS), and high quality rating only for one generalized VoA instrument (AAQ). Internal consistency was rated with high quality for six generalized VoA (EAS, AAQ, RSQ-Age, MSASA, AS, NEBA) and for six personal VoA instruments (AARC, SAPS, MFQ, MCI, AgeCog, PAS). For construct validity, three personal VoA instruments showed moderate quality (AARC, AtoA, MFQ) and one high quality (PAS), while only one generalized VoA instrument received a high rating (RSQ-Age) and two a moderate rating (AAQ, ERA). Regarding reliability, only one generalized VoA instrument (IAS) showed moderate quality, while none of the personal VoA instruments obtained adequate quality of evidence. Furthermore, cross-cultural and criterion validity were assessed for only a few instruments, with low-quality evidence (four and five instruments, respectively), while neither measurement error nor responsiveness has yet been assessed.

In addition, among the nine instruments identified as assessing or including items related to older adults’ views on age-related cognitive changes, there was considerable heterogeneity in the quality of their psychometric properties. These instruments received inconsistent evaluations for content validity (6 out of 9), with only ERA and PAS rated as moderate quality. While most instruments demonstrated high-quality structural validity (five out of nine) and internal consistency (seven out of nine), the quality of evidence for reliability was low (five out of six). Only a few instruments provided adequate assessment of construct validity (PAS, MCI, AARC, ERA) and measurement invariance (AARC, MFQ, PAS). As previously mentioned, neither measurement error nor responsiveness has been assessed to date. Both the assessment of psychometric properties of the existing VoA instruments considered here, and the development of new, high-quality, psychometrically sound instruments examining also generalized and personal views of cognitive aging in older adults should therefore be considered by researchers.

Nonetheless, the VoA classification proposed here could guide professionals in selecting generalized VoA instruments capable to assess socially shared beliefs and stereotypes and drive the implementation of clinical interventions or campaigns to eradicate incorrect, negative views of the aging process. At the same time, personal VoA instruments, including those designed to depict older adults’ views of their cognitive changes with aging, could serve to promote a more flexible and adaptive perspective/view on the aging process, particularly in those individuals at risk of developing neurocognitive disorders (e.g. Mild Cognitive Impairment or Alzheimer’ disease), or in the prodromal phase of cognitive decline (e.g. Subjective Cognitive Decline), to promote healthy aging. Holding ageist/stereotypical, negative VoA, particularly with respect to one’s own cognitive functioning, represents, in fact, one of the most striking psychological barriers to the promotion of healthy aging (WHO Decade of Healthy Aging; [109]): it may discourage not only older adults but also middle-aged adults from engaging in cognitively stimulating activities and adopting health-promoting behaviors (e.g. cognitive stimulation/training programs, and well-known proxies of cognitive reserve, e.g. [110]). To enhance the efficacy of cognitive stimulation/training programs for older adults [21, 110], for instance, the inclusion of a pretraining phase triggering and ameliorating views of cognitive aging facets, such as eradicating negative stereotypes, conveying a malleable view of the cognitive aging process and the key proactive role that individuals could have in shaping it, should be considered. Furthermore, examining VoA with a focus on age-related cognitive changes facets among older adults with poor memory performance and subjective memory complaints (see [111]) could contribute to creating more individualized and engaging training programs and clinical activities aimed at changing their negative mindset and attributions about memory functioning, and promoting their self-efficacy regarding memory and its controllability in older age [112].

Some limitations of this review process should also be acknowledged. Formulating inclusion criteria for subjective VoA instruments was challenging, given broad and multifaced nature of the construct. Despite following PRISMA guidelines and using the snowballing method, some other relevant studies may have been missed. To ensure comprehensiveness and relevance, we included only instruments validated on samples of older adults, excluding those developed on younger populations. Following the COSMIN guidelines, we evaluated the GRADE assessment of instruments, even if they were used in only one validation study. This approach might still provide a comprehensive picture of the evidence for all the measures included, but further evidence is necessary to attain a more extensive and higher-quality body of evidence. Furthermore, considering the importance of also evaluating subjective views of one’s own (age-related) cognitive changes in prevention and clinical interventions (e.g., cognitive and metacognitive training for older adults; [21]), future instruments should be developed to examine more specifically subjective VoA related to cognitive functioning in older age.

In conclusion, this systematic review presents the available instruments used to examine older adults’ perceptions, attitudes, and expectations related to old age in general and their own aging process. It offers insights into the strengths and weaknesses, also with respect to the psychometric and measurement properties, of various generalized and personal VoA instruments for older adults, here newly categorized according to the recent theoretical VoA reconceptualization. Future research in this field should be devoted to further addressing an underrepresented domain in the multidimensionality of the VoA construct: the perception of age-related changes in cognitive functioning, so crucial in older adults’ everyday life functioning, and which could allow obtaining insights for clinical and applied research and practice for promoting older adults’ quality of life and healthy aging.

## Supplementary Information

Supplementary Material 1.

## Data Availability

The data that support the findings of this study are available from the corresponding authors on reasonable request.

## Footnotes

1 After identifying studies through the database literature search, we used the “cited by” function in Google Scholar (which can also be useful for grey literature) on the original validation articles. This process led to the identification of 45 additional validation studies across all included instruments (until February 2025).
