# Supplementary material for: Examining subjective views of the aging process in older adults: a systematic review following the COSMIN methodology
Source: BMC Geriatr. 2025 Nov 13;25:897. doi: 10.1186/s12877-025-06472-w (PMC12616921; doi:10.1186/s12877-025-06472-w)
Supplement: Supplementary file 1 — Supplementary Material 1. [file 12877_2025_6472_MOESM1_ESM.docx]

**Examining Subjective Views of the Aging Process in Older Adults: A Systematic Review following the COSMIN methodology**

SUPPLEMENTARY MATERIAL

Summary of the content:

PART 1 – Details of inclusion criteria

PART 2 – Details of study eligibility process

PART 3 – Details of results

PART 4 – Description of reviewed instruments

**PART 1 – DETAILS OF INCLUSION CRITERIA**

**Table S1.** *Inclusion and exclusion criteria used for the study selection.*

| **Study variables** | **Inclusion criteria** | **Exclusion Criteria** |
| --- | --- | --- |
| *P (Population)* | The targeted population consisted of community dwelling, autonomous, typically aging older adults aged 55 years and over (males and females). We refer to older adults with a normal (typical) aging process as those experiencing normal age-related changes (including in cognition) and trajectories across various health and quality of life domains, occurring without the presence of pathological conditions or disorders (e.g. Craik & Salthouse, 2011). Eligible articles must have an explicit focus on measurement in older age (defined here as starting at age 55 because age-related changes in several health and cognitive variables become more evident as early as age 50; see also Salthouse, 2009). | Participants who were < 55 years old, clinical populations (e.g., people with mild cognitive impairment, dementia, or other neurological or psychiatric conditions, institutionalized older adults). Studies with a focus on young and middle-aged adults were not considered unless they include a separate sample of older adults. |
| *I (Instruments)* | Validated measurement instruments designed for use with older adults (e.g., scales, assessment tools) and self-report measures (e.g., questionnaires or single-item questions) to assess subjective VoA as well as age-related cognitive changes. These measures should be validated using a sample of older adults and encompass domains/concepts of subjective views and perceptions of aging. | Any measures and self-reported instruments that were not specifically validated for use with older adults and diagnostic tools or on the assessment of specific mental health conditions. Performance tests, cognitive tests, language assessments, biometric tests, and performance-based functional behavioral assessments were also excluded. |
| *C (Construct)* | Constructs related to subjective VoA as well as specific aging process effects on cognitive domains in older age (age-related cognitive changes), focusing on individuals’ perceptions, beliefs, attitudes, and expectations regarding their aging process and aging in general. | Constructs not concerning subjective VoA or specific aging process effects on cognitive domains as well as those that were primarily focused on the clinical setting (i.e., pathological process) or targeted these constructs in younger age groups. |
| *O (Outcomes)* | Two main outcomes of interest. (1) Measurement characteristics and psychometric properties of the measurement instruments. The measurement characteristics included psychological domains that assessed subjective VoA as well as views on cognitive aging. (2) The psychometric properties included the measurement instruments’ validity, consistency, and reliability. |  |
| *S (Study design)* | Eligible articles used in validation or reliability studies on the measurement properties. Articles published in peer-reviewed journals and assessment handbooks (if accessible online) in English or Italian. | Publications such as conference abstracts, case studies, protocols, dissertations, and systematic reviews. Instruments developed for acute care, mental health care, palliative care, primary care, or hospitalized settings. Also, those instruments that assessed transfer from or to any of the aforementioned care settings. |

**Table S2**. *Search queries for databases on instruments assessing subjective views of the aging processes in older adults.*

| **Database** | **Search query** |
| --- | --- |
| *Scopus* | ( TITLE-ABS-KEY ( ( "older adult*" OR "older people" OR "elderly" ) ) AND TITLE-ABS-KEY ( ( "cognitive concerns" OR "awareness of age-related cognitive change*" OR "cognitive perceptions" OR "aging concerns" OR "felt age" OR "aging awareness" OR "awareness of age-related change*" OR "subjective aging" OR "views on aging" OR "age perception" OR "self-perceptions of aging" OR "age-related belief*" OR "belief* about aging" OR "belief* on aging" OR "age stereotyp*" OR "aging stereotyp*" OR "ageism" OR "myth* of aging" OR "attitud* toward aging" ) ) AND TITLE-ABS-KEY ( ( "Survey" OR "Scales" OR "Tools" OR "Measurement*" OR "Instrument*" OR "Assessment*" OR "Validity" OR "reliability" OR "consistency" OR "screening" ) ) ) |
| *Web of Science* | (“older adult*” OR “older people” OR “elderly”) (All Fields) and (“cognitive concerns” OR “awareness of age-related cognitive change*” OR “cognitive perceptions” OR “aging concerns” OR “felt age” OR “aging awareness” OR “awareness of age-related change*” OR “subjective aging” OR “views on ageing” OR “age perception” OR “self-perceptions of aging” OR “age-related belief*” OR “belief* about aging” OR “belief* on aging” OR “age stereotyp*” OR “aging stereotyp*” OR “ageism” OR “myth* of aging” OR “attitud* toward aging”) (All Fields) and (“Survey” OR “Scales” OR “Tools” OR “Measurement*” OR “Instrument*” OR “Assessment*” OR “Validity” OR “reliability” OR “consistency” OR “screening”) |
| *PycInfo* | ((“older adult*” OR “older people” OR “elderly”)) AND ((“cognitive concerns” OR “awareness of age-related cognitive change*” OR “cognitive perceptions” OR “aging concerns” OR “felt age” OR “aging awareness” OR “awareness of age-related change*” OR “subjective aging” OR “views on ageing” OR “age perception” OR “self-perceptions of aging” OR “age-related belief*” OR “belief* about aging” OR “belief* on aging” OR “age stereotyp*” OR “aging stereotyp*” OR “ageism” OR “myth* of aging” OR “attitud* toward aging”)) AND ((“Survey” OR “Scales” OR “Tools” OR “Measurement*” OR “Instrument*” OR “Assessment*” OR “Validity” OR “reliability” OR “consistency” OR “screening”)) |

**PART 2 – DETAILS OF STUDY ELIGIBILITY PROCESS**

**Figure S1** *Algorithm for the suggested judgement of the study eligibility process (Y= yes, N= no, PY= probably yes, PN= probably not).*

**

**PART 3 – DETAILS OF RESULTS**

**Table S3.** *Details of the measurement instruments and their measurement properties retrieved by the validation studies.*

| **SCALE NAME** | **AUTHORS** | **STUDY DESIGN** | **COUNTRY** | **SAMPLE** | **SCALE CHARACTERISTICS** | **RESULTS (MEASUREMENT PROPERTIES)** | |
| --- | --- | --- | --- | --- | --- | --- | --- |
|  |  |  |  |  |  | **RELIABILITY** | **VALIDITY** |
| **GENERALIZED VOA** | | | | | | | |
| **ACTIVE AGEING AWARENESS QUESTIONNAIRE (AAAQ)** | *Bahuri et al., 2021 | Cross-sectional | Malaysia | Adults and community-dwelling OA  *N:* Sample 1 = 110; Sample 2 = 81; Sample 3 = 404  *Age:* Sample 1: M = 50.19, SD = 5.52; Sample 2: M = 49.40, SD = 5.70; Sample 3: 49.90, SD = 5.80  *Gender:* Sample 1 = 34.5% F; Sample 2 = 42% F; Sample 3 = 48.5% F | *N ° of items:* 14 + 2 standalone questions (The first question asks if the participants have heard the term ‘active ageing’. The second question is an open-ended question, where participants are asked to give their opinion about factors that may help them to age actively)  *Dimension(s):* total score and 2 dimensions (health, non-health)  *Response scale:* 4-point Likert scale (from “strongly disagree” to “strongly agree”) | **INTERNAL CONSISTENCY:**   - ***Composite reliabilities***   - Inter-item correlation was used to assess redundancy among items. The CR for the health and non-health subscales were 0.91 and 0.89, respectively.  - AVE were 0.56 for the health and 0.58 for the non-health sub-scales, respectively.  - Cronbach’s α were 0.92 for health and 0.91 for non-health subscales, respectively.   - ***Multi-item scale reliability***   - Both the health and participation constructs had CITC below 0.3. The CITC for the security construct was 0.711.  **RELIABILITY:**   - ***Test-retest stability***   - ICC values for the final set of items ranged from 0.489 to 0.739. | **STRUCTURAL VALIDITY:**   - ***Exploratory Factor Analysis***   -The initial EFA revealed a four factors structure (67% of variance explained): factor 1 = 10.97, factor 2 = 1.86, factor 3 = 1.08, factor 4 = 0.63  - Parallel analysis also revealed a four factors structure: factor 1 = 1.74, factor 2 = 1.63, factor 3 = 1.53, factor 4 = 1.43.  - EFA was repeated, but the number of factors was fixed at two by removing those with eigenvalues too close or less than 1. This model explained 58% of variance with a KMO = 0.92 (p < .001)   - ***Confirmatory Factor Analysis (CFA)***   - A two-dimensional structure consisting of a health and a non-health construct was confirmed through the CFA, after testing four different models (CMIN/df = 2.77, GFI = 0.90, AGFI = 0.86, TLI = 0.95, CFI = 0.94, RSMEA = 0.08). |
| **AGE-BASED REJECTION SENSITIVITY QUESTIONNAIRE (****RSQ-Age)** | *Kang & Chasteen, 2009 | Cross-sectional | Canada | Community-dwelling OA  *N:* Sample 1 = 103; Sample 2 = 68  *Age:* Sample 1: M = 71.32, SD = 6.38 (60-87); Sample 2: M = 71.35, SD = 6.12 (60-80)  *Gender:* Sample 1 = 49% F; Sample 2 = 53% F | *N° of items:* 15  *Dimension(s):* unidimensional  *Response scale:* 6-point Likert scale (from “very unconcerned” to “very concerned”) | **INTERNAL CONSISTENCY:**  *Sample 1:*  - Cronbach’s α was 0.91.  - Each of the item-total correlations was greater than 0.44.  *Sample 2:*  - Cronbach’s α was 0.88.  - Each of the item-total correlations was greater than 0.41.  **RELIABILITY:**   - ***Test-retest stability***   - The 1-year test-retest reliability of the scale was high (r = 0.74, p < .01), indicating stability of the RSQ-Age construct and measure. | **STRUCTURAL VALIDITY:**   - ***Exploratory Factor Analysis***   - PCA was conducted on the 15 product scores obtained for each scenario.  - Based on the scree test, only one component was retained, which had an eigenvalue of 6.45 and accounted for 43% of the total variance.  **CONSTRUCT VALIDITY:**   - ***Convergent validity***   ***Correlations:***  - RSQ-Age showed significant positive correlations with awareness of ageism (r = 0.34, p < .01), self-consciousness (r = 0.31, p < .01), RS-personal (r = 0.36, p < .01), and age-based stigma consciousness (r = 0.27, p < .01). RSQ-Age also showed a significant negative relationship with self-esteem (r = - 0.49, p < .01). |
| **ATTITUDES TO AGEING QUESTIONNAIRE (AAQ)** | Laidlaw et al. 2007 | Cross-sectional | Spain, England, Israel, Hungary, Denmark, Scotland, China, Germany, Norway, Czech Republic, United States, Japan, Sweden, Canada, Lithuania | Community-dwelling and older residents  *N:* Sample 1 = 1,356; Sample 2 = 5,566  *Age:* Sample 1 = 60-99; Sample 2 = 60-100  *Gender:* Sample 1 = 60% F; Sample 2 = 59% F | *N° of items:* 24  *Dimension(s):* 3 dimensions (psychological growth, psychosocial loss, and physical change)  *Response scale:* 5-point Likert scale (from “not all true” to “extremely true”) | **INTERNAL CONSISTENCY:**  - Cronbach’s α were 0.75 for psychological growth, 0.84 for psychosocial loss, 0.68 for physical change, and 0.86 for the total.  - PSIs (the IRT equivalent of Cronbach alpha analyses) for each scale were adequate (0.81, 0.81, and 0.74, respectively). | **STRUCTURAL VALIDITY:**   - ***Exploratory Factor Analysis***   - PCA followed by Varimax rotation. The analyses point to three- and five- factor solutions; the four-factor and six-factor and upwards solutions all contained non-viable factors consisting of only two items. Visual inspection of the Scree Plot also strongly supported the three-factor solution.   - ***Confirmatory Factor Analysis***   - CFA for the 24-item scale showed good fit indices for a three correlated factor model (χ^2^ = 4559.9, df = 248, CFI = 0.84, RMSEA = 0.06), which showed significant further improvement by allowing two or three items to cross-load onto other subscales.   - ***Item Response Theory***   - Both RUMM and WINMIRA analyses were conducted on the initial set of 31 items, as well as on each subscale, to assess item characteristics and test the underlying IRT model. The net effect of the RUMM and the WINMIRA analyses was the production of three eight-item scales that retained the following items  **MEASUREMENT INVARIANCE**:   - ***Item Response Theory***   - DIF was tested for the factor Centre (European vs non-European centers), age group and gender. No DIF was found, except for Scale 2 items for the Centre factor and age group. |
| 12-AAQ | Laidlaw et al. 2018 | Cross-sectional | Spain, England, Israel, Hungary, Denmark, Scotland, China, Germany, Norway, Czech Republic, United States, Japan, Sweden, Canada, Lithuania | Community-dwelling and older residents  *N:* Sample 1 = 2,487; Sample 2 = 2,488; Sample 3 = 792  *Age:* Sample 1: M = 72.3, SD = 8 (60-97); Sample 2: M = 72.2, SD = 8.1 (60-99); Sample 3: M = 74, SD = 0.3 (73-74)  *Gender:*  Sample 1 = 57.3% F; Sample 2 = 57.4% F; Sample 3 = 48.6% F | *N° of items:* 12 | **INTERNAL CONSISTENCY:**  - Cronbach’s α for the psychosocial loss and physical change subscales of 12-item AAQ-SF were above 0.70, maintaining high consistency with the AAQ; thus, this format is adopted for the AAQ-SF. The psychological growth subscale of AAQ-SF demonstrated a lower alpha coefficient (α = 0.62). | **STRUCTURAL VALIDITY:**   - ***Exploratory Factor Analysis***   - The selected 12 items support a 3-factor solution with eigenvalues greater than 1 explaining 40% of variance.   - ***Confirmatory Factor Analysis***   - The data support a 3-factor structure identical to that of the original AAQ, providing an adequate fit for the data. Sample 2: χ^2^ = 807.4, df = 51, p < .001, CFI = 0.88, RMSEA = 0.08, TLI = 0.84. Sample 3: χ^2^ = 314.2, df = 51, p < .001, CFI = 0.87, RMSEA = 0.08, TLI = 0.83.  **CONSTRUCT VALIDITY:**   - ***Convergent and divergent validity***   ***T-tests:***  - In general, individuals defining their current QOL as very poor (“unhealthy”) reported statistically significant negative attitudes compared with those who identified their QOL as very good (“healthy”) (Cohen’s d: psychosocial loss = 0.81; physical change = 1.17; psychological growth= 0.27). Individuals with depressive symptoms (HADS-D/A ≥ 8) correlated to significantly more negative attitudes on all AAQ-SF subscales (psychosocial loss: t = 6.78, p < 0.01, d = 1.24; physical change: t = 3.47, p < 0.01, d = 0.63; psychological growth: t = 2.11, p < 0.05, d = 0.39) compared to individuals with low depression levels (HADS-D/A <8).. Similar patterns were found for anxiety (psychosocial loss: t = 5.27, p < 0.01, d = 0.48; physical change: t = 2.28, p < 0.05, d = 0.21; psychological growth: t = 0.57, n.s.).. |
| 22-AAQ | Chachamovich et al., 2008 | Cross-sectional | Brazil | Community-dwelling OA  *N:* Sample 1 = 424, Sample 2 = 5,238  *Age:* old *=* 60–69; old-old = 70-79: very old = +80  *Gender:* Sample 1 = 64.2% F; Sample 2 = 57.9% F | *N° of items:* 22  *Response scale*: 4-point Likert scale | **INTERNAL CONSISTENCY:**  - The Brazilian dataset showed Cronbach’s α of .86 for the psychosocial loss subscale (and 0.85 for the international dataset), 0.80 for the physical change subscale (0.82 for the International dataset) and 0.67 for the psychological growth subscale (0.70 for the International  dataset).  - PSIs (the IRT equivalent of Cronbach α analyses) for each scale were all good and were 0.82 for the psychosocial loss subscale, 0.75 for the physical change subscale, and 0.71 for the psychological growth scale. | **STRUCTURAL VALIDITY:**   - ***Exploratory Factor Analysis***   - Data were initially examined through a PCA with Varimax rotation. The three-factor solution accounted for 34.45% of the total variance, whereas in the international sample the same structure was responsible for 32.74%.   - ***Confirmatory Factor Analysis***   - Similarly to the international findings, the 24-item version showed the best structure (χ^2^ = 645.19 p = .061, df = 249, CFI = 0.83, RMSEA = 0.06).   - ***Item Response Theory***   - Responses were tested according to the Rasch model for polytomous scales. Analysis using the new 4-point scale showed that all subscales had remarkable improvement, with no model or item misfitting (22-item). Psychosocial loss: χ^2^ = 77.06, df = 40, p < .001, PSI = 0.87; physical chance: χ^2^ = 109.4, df = 48, p < .001, PSI = 0.81, psychological growth: χ^2^ = 59.06, df = 48, p = .13, PSI = 0.75.  **MEASUREMENT INVARIANCE:**   - ***Item Response Theory***   - DIF was tested for the levels of depression, age group and gender. No DIF was found in the final model (22-item and 4-point response scale).  **CONSTRUCT VALIDITY:**   - ***Divergent validity***   ***Correlations:***  ***-*** GDS scores significantly correlated with 22-AAQ subscales (psychosocial loss = -0.59; psychological growth = -0.59; physical change: r = -0.35). |
| 15-AAQ | de Lima et al., 2022 | Cross-sectional | Portugal | Community-dwelling OA  *N:* 400  *Age:* M = 56.76, SD = 18.75 (18-95)  *Gender:* 64.5% F | *- N° of items:* 15 | **INTERNAL CONSISTENCY:**  - CR of the three factors were acceptable (Cronbach’s α ranging from 0.65 to 0.84) | **STRUCTURAL VALIDITY:**   - ***Confirmatory Factor Analysis***   - CFA confirmed the 3-factor structure with acceptable fit: χ^2^ = 287.32, df = 87, p < .001, CFI = 0.87, GFI = 0.90, TLI = 0.84, RMSEA = 0.08.  **MEASUREMENT INVARIANCE**  - The multi-group analysis was used to study the invariance of the model. Results showed that the model invariance is demonstrated when compared in two different samples.  **CONSTRUCT VALIDITY:**   - ***Convergent validity***   - It was also not possible to fully verify convergent validity, since the AVE values are below 0.50, the minimum recommended value for this index. |
| AAQ | Kalfoss et al., 2010 | Cross-sectional | Canada & Norway | Community-dwelling OA  *N:* Canadian = 202; Norwegian = 490  *Age:* Canadian: M = 72.32, SD = 7.8 (60-95); Norwegian: M = 77.62, SD = 7.2 (60-91)  *Gender:* Canadian: 54% F; Norwegian: 57.6% F | *-* | **INTERNAL CONSISTENCY:**  **-** Cronbach’s α for  Canadian and Norwegian samples were = 0.86 and = 0.82, respectively. For the three factors, the Cronbach’s α values ranged from 0.70 to 0.79. | **STRUCTURAL VALIDITY:**   - ***Confirmatory Factor Analysis***   - CFA showed that all 24 items in the AAQ were retained (p < .001), even though goodness of fit between the hypothesized measurement model and the Canadian study sample data was not achieved (χ^2^ = 603.11, df = 249, p < .001, GFI = 0.80, CFI = 0.76, RMSEA = 0.08). This same model, tested using the data from Norway, resulted in slightly better fit statistics (χ^2^ = 913.78, df = 249, p < .001, GFI = 0.85, CFI = 0.74, RMSEA = 0.07)  **CONSTRUCT VALIDITY:**   - ***Convergent validity***   For both Norway and Canada samples, all AAQ subscales (psychosocial loss, physical change, psychological growth) positively correlated with all WHOQOL-BREF domains (physical and psychological health, social relationship, environment) (Norway: r ranging from 0.229 to 0.606; Canada: r ranging from 0.265 to 0.633, all ps<.001), all WHOQOL-OLD domains (sensory ability, autonomy, past, present and future activities, social participation, death and dying, intimacy) (Norway: r ranging from 0.176 to 0.474; Canada: r ranging from 0.289 to 0.650, all ps<.001, expect for sensory ability [p>.05] and death and dying [p>.05]), and health satisfaction (Norway: r ranging from 0.226 to 0.533; Canada: r ranging from 0.310 to 0.617, all ps<.001).   - ***Divergent validity***   For both Norway and Canada samples, all AAQ subscales negatively correlated with GDS (Norway: r ranging from -0.305 to -0.510; Canada: r ranging from -0.374 to -0.620, all ps<.001). |
| AAQ | Lucas-Carrasco et al., 2013 | Cross-sectional | Spain | Community-dwelling OA  *N:* 242  *Age:* M = 71.10, SD = 7.10 (60-94)  *Gender:* 60.3% F | *-* | **INTERNAL CONSISTENCY:**  - Cronbach’s α was 0.59 for psychological growth, 0.73 for physical change, and 0.70 for psychosocial loss. | **STRUCTURAL VALIDITY:**   - ***Exploratory Factor Analysis***   - After conducting principal components factor extraction and Varimax rotation, the solution revealed a three-factor model that accounted for 34% of the variance.  **CONSTRUCT VALIDITY:**   - ***Convergent validity***   ***Correlations*:**  - Moderate correlations were found between the AAQ physical change and psychosocial loss subscales with the physical and psychological WHOQOL-BREF domains, the WHOQOL-OLD, the total GDS, and the PCS-12   - ***Known-group validity***   ***T-Tests*:**  - Men scored higher on psychosocial loss (t(219) = 2.229, p = .027) than women. Higher educational level was linked to higher physical change (t(211) = -2.138, p = 0.034) and lower psychosocial loss (t(217) = 2.641, p = 0.009). Lower depressive symptoms (GDS-30 < 11) and fewer chronic conditions (<4) were associated with higher physical change and lower psychosocial loss. Participants from primary care and community centers scored higher on physical change and psychological growth than caregivers. |
| AAQ | Marquet et al., 2016 | Cross-sectional | Belgium | Community-dwelling OA  *N:* 238  *Age:* M = 73.84, SD = 8.66  *Gender:* 66.4% F | *-* | **INTERNAL CONSISTENCY:**  - Internal consistency measured by Cronbach’s α was .81 for the total score, 0.75 for physical change, 0.62 for psychological growth and 0.74 for psychosocial loss.  **TEST-RETEST RELIABILITY:**  - Test–retest correlations were high for the total score (*r* = 0.79; *p* < .001) and all sub­scales (0.77 for psychological growth, 0.81 for physical change and 0.76 for psychosocial loss; all *p* < .001). | **STRUCTURAL VALIDITY:**   - ***Exploratory Factor Analysis***   - Eight factors with eigenvalues >1 emerged, but the scree test and parallel analyses suggested three or four factors, explaining 42.75% and 36.90% of variance, respectively. The four-factor solution had one trivial factor with only three items loading ≥ 0.40, so the three-factor solution was chosen.  **CONSTRUCT VALIDITY:**   - ***Convergent validity***   ***Correlations:***  - Correlations between AAQ and subjective age, WHOQOL-OLD, health, and depression showed that more positive attitudes toward aging were associated with feeling younger (r = –0.34, p < .001), greater quality of life (r = 0.51, p < .01), better subjective mental (r = 0.30, p < .001) and physical health (r = 0.46, p < .001), and lower levels of depression (r = –0.40, p < .001). Regarding subscales, a more positive view of physical functioning (Physical Change) was related to lower negative impact of sensory losses (r = 0.37, p < .05), feeling younger (r = –0.35, p < .001), better mental (r = 0.24, p < .01) and physical health (r = 0.49, p < .001). Higher psychosocial loss was linked to lower satisfaction with past and future activities (r = –0.40, p < .05), poorer physical (r = –0.40, p < .001) and mental health (r = –0.35, p < .001), and feeling older (r = 0.20, p < .05). Depressive mood was also associated with higher psychosocial loss (r = 0.37, p < .001). Finally, perceiving aging as a time of wisdom and gains (Psychological Growth) was related to greater satisfaction with past and future experiences (r = 0.50, p < .01).   - ***Known-groups validity***   ***Mann-Whitney U test:***  - No differences were found between men and women for the total score or AAQ subscales. Higher education was associated with higher total scores (p < 0.01) and Physical Change (p = 0.02), but with lower Psychosocial Loss (p < 0.01). |
| AAQ | Rejeh et al., 2017 | Cross-sectional | Iran | Community-dwelling OA  *N:* 400  *Age:* old = 60-70; old-old =71-80; very old = 81-90  *Gender:* 54.5% F | *-* | **INTERNAL CONSISTENCY:**  - Cronbach’s α coefficient was reported as 0.90 for the total score, 0.85 for physical change, 0.91 for psycho­logical growth and 0.93 for psychosocial loss.  **TEST-RETEST RELIABILITY:**  - Correlations were high for the total score and all dimensions (*r* = 0.32 to *r* = 0.76; *p* < .001).  - ICC for the total instrument was reported as 0.74–0.88. | **CONTENT VALIDITY:**  - The AAQ's content validity was assessed by six specialists using the CVI and S-CVI/Ave method, with an S-CVI/Ave of 0.92.   - ***Face validity***   **-** Face validity was conducted by 15 older people.  **STRUCTURAL VALIDITY:**  ***Exploratory Factor Analysis***- Three factors were extracted and identified using a minimum eigenvalue of 1 as the factor criterion. The visual inspection of the scree plot strongly supported the suggested three factor solutions accounting for 76.87% of the total variance.  **CONSTRUCT VALIDITY**   - ***Convergent validity***   ***Correlations*:**  - AAQ showed large correlations with WHOQOL-BREF (r = 0.7) and SF-36 (r = 0.5–0.7). Positive physical functioning was linked to fewer sensory loss impacts on QoL (r = 0.56, p < .001; r = 0.78, p < .001).   - ***Known-group validity***   Participants with higher educational levels scored higher on the AAQ total score (p < 0.001). For AAQ subscales, they scored higher on Physical Changes (p = 0.060) but lower on Psychosocial Loss (p < 0.010), with no significant differences in Psychological Growth (p > 0.05). No significant gender differences were found (all p > 0.05), though men scored higher on most domains. |
| 19-AAQ | Rejab et al., 2022 | Cross-sectional | Malaysia | Community-dwelling OA  *N:* 254  *Age:* M = 66.46, SD = 5.37 (60-88)  *Gender:* 51.2% F | *N° of items:* 19 | **INTERNAL CONSISTENCY:**  - Cronbach’s α of the final version of the 19 items AAQ total score was of 0.79. Cronbach’s α of the physical change (7 items), psychosocial loss (8 items), and psychological growth (4 items) factors were 0.74, 0.82, and 0.71, respectively. | **CONTENT VALIDITY:**  - An expert panel (a psychiatrist, geriatrician, and family physician) assessed the content validity and confirmed that all 24 items were relevant and culturally appropriate for Malaysia.   - ***Face validity***   -The final version of AAQ was administered to 10 older adults to assess face validity. Participants reported the AAQ questions were relevant, easy to understand, and culturally appropriate. They completed the questionnaire independently in 10–15 minutes.  **STRUCTURAL VALIDITY:**   - ***Exploratory Factor Analysis***   - An initial EFA identified five factors explaining 40.9% of the variance, but the scree plot suggested three factors. A second EFA with three factors explained 37.9% of the variance. |
| 12-AAQ | Gao et al., 2024 | Cross-sectional | China | Community-dwelling OA  Sample 1  *N:* 442  *Age:* M = 68.62, SD = 5.55  *Gender:* 217 F  Sample 2  *N:* 311  *Age:* M = 65.73, SD = 4.52  *Gender:* 214 F  Sample 3  *N:* 164  *Age:* M = 68.03, SD = 4.72  *Gender:* 109 F | *N° of items for Sample 1: 24*  *N° of items for Samples 2 and 3: 12* | **INTERNAL CONSISTENCY**  - Cronbach’s α for subscales ranged from 0.76 to 0.80 for Sample 1 (24-AAQ).  - Sample 2: α = 0.80 for psychosocial loss, 0.73 for physical change, and 0.71 for psychological growth)  - Sample 3: α = 0.65 for psychosocial loss, 0.75 for physical change, and 0.74 for psychological growth).  **RELIABILITY:**   - ***Test-retest stability***   - Retest (after 2-3 weeks): *r* ranging from 0.77 to 0.88. | **STRUCTURAL VALIDITY**   - ***Exploratory Factor Analysis***   *-* KMO and Bartlett test statistic were computed: KMO = 0.81 and *p* <.001 for Bartlett’s test of sphe­ricity.   - ***Confirmatory Factor Analysis***   - Sample 2: χ2/df = 2.343, GFI = 0.941, RMSEA = 0.066, CFI = 0.933, and SRMR = 0.058. -Sample 3: χ2/df = 1.986, GFI = 0.907, RMSEA = 0.078, CFI = 0.896, and SRMR = 0.082.  **CONSTRUCT VALIDITY:**   - ***Convergent validity***   - Subscales of AAQ significantly correlated with depression (CES-D; [psychosocial loss: r=0.46, physical change: r=-0.46, psychological growth: r=-0.39]), anxiety (GAD; [psychosocial loss: r=0.38, physical change: r=-0.30, psychological growth: r=-.23]), and quality of life (WHOQQL-Bref; [physical change: r=-0.42, psychological growth: r=-0.34]).   - ***Discriminant validity***   - Significant differences between individuals with “excellent”/“good” self-rated health vs those with “fair”/“bad”/“poor” in the psychosocial loss and physical change subscales: (t = 3.28, p =.001 for psychosocial loss; and t = 4.85, p <.001 for physical change). No significant difference in the psychological growth subscale (t = 1.22, p =.224) was found. Similar patterns of results were obtained when comparing participants with vs without chronic disease (psychosocial loss: t = 2.41, p =.017; physical change: t = 3.39, p <.001; psychological growth: t = 0.67, p =.504). |
| 12-AAQ | Low et al., 2024 | Cross-sectional | Canada | Adults and Community-dwelling OA  *N:* 517  Age: M= 56.28, SD = 2.82  *Gender:* 409 F |  | **INTERNAL CONSISTENCY**  Cronbach’s α = 0.78 for psychological growth, 0.61 for psychosocial loss, and 0.81 for physical change, respectively. | **STRUCTURAL VALIDITY:**   - ***Confirmatory Factor Analysis***   - The three-factor structure was confirmed: χ2=454.12, df= 205, CFI= 0.866, TLI= 0.849, RMSEA [90% CI]= 0.049 [0.043–0.055].  **CONSTRUCT VALIDITY:**   - ***Convergent validity***   - 12-AAQ subscales correlated positively with RSES scores: Psychosocial Loss with Self-Deprecation (λ = 0.51) and Self-Confidence (λ = 0.42); Psychological Growth with Self-Confidence (λ = 0.47) and Self-Deprecation (λ = 0.29); and Physical Change with Self-Confidence (λ = 0.35) and Self-Deprecation (λ = 0.34). |
| AAQ | Zadworna et al., 2025 | Cross-sectional | Poland | Community-dwelling OA  Pilot study  *N:* 50  *Age range:* 60-84  *Gender:* 37 F  Study  *N:* 500  *Age:* M=66.75, SD= 5.10  *Gender:* 269 F |  | **INTERNAL CONSISTENCY:**  - Cronbach’s α = .84 for psychosocial loss, α = .78 for physical change, α = .73 for psychological growth, α = .89 for total score.   - ***Test-retest stability***   - After three weeks: r_tt_ = .87 (p < .001) for total score; psychosocial loss: r_tt_ = .80, p < .001; physical change: r_tt_ = .83, p < .001; psychological growth: r_tt_ = .75, p < .001.  - Intraclass correlation coefficients (ICC): .93, with a 95% CI [.91, .95] for total score; ICC = .89, 95% CI [.86; .91] for psychosocial loss; ICC = .91, 95% CI [.88; .93] for physical change; ICC = .86, 95% CI [.81; .89] for psychological growth | **CONTENT VALIDITY:**  Pilot study: feedback regarding the comprehensibility of instructions and questions.  **STRUCTURAL VALIDITY:**   - ***Confirmatory Factor Analysis***   - The factor structure was confirmed: CMIN/df = 3.58, RMR = 0.07, CFI = 0.85, RMSEA = 0.07, 95% CI [0.06-0.08], GFI = 0.86, AGFI = 0.83, NFI = 0.81, IFI = 0.86, TLI = 0.83.   - ***Convergent validity***   - AAQ total score correlated with Personal Wellbeing Index [PWI] (r= 0.52), Self-Rated Health [SRH] (r= 0.52), and Geriatric Depression Scale [GDS] (r=-0 .69); psychosocial loss correlated with PWI (r= .41), SRH (r= 0.41), and GDS (r=- 0.64); physical change correlated with PWI (r= 0.64), SRH (r= 0.64), and GDS (r=- 0.63); psychological growth correlated with PWI (r= 0.21), SRH (r= 0.21), and GDS (r=- 0.42). |
| **EVERYDAY AGEISM SCALE (EAS)** | *Allen et al., 2022 | Cross-sectional | United States | Community-dwelling OA  *N:* Phase I = 100; Phase II = 2,048  *Age:* 50–80  *Gender: Phase I =* 47% F, Phase II = 52.4% F | *N° of items:* 10  *Dimension(s):* total score and 3 dimensions (exposure to ageist messages, ageism in interpersonal interactions, internalized ageism)  *Response scale:*  Items 1-7 with a 4-point Likert scale (“never”, “rarely”, “sometimes”, “often”). Items 8–10 with a 4-point Likert scale (“strongly disagree”, “disagree”, “agree”, “strongly agree”). | **INTERNAL CONSISTENCY:**  - Readability statistics, assessed using the Flesch-Kincaid grade level, indicated that the text was at an eighth-grade reading level or lower.  - Cronbach’s α coefficients for the subscales were all above 0.70. The overall scale had a Cronbach’s α = 0.77.  - The 10 items were retained in the analysis because the correlations between the items within each factor were deemed adequate, with values greater than 0.3. | **STRUCTURAL VALIDITY:**   - ***Exploratory Factor Analysis***   - EFA showed a three-factor structure after 21 iterations of rotation (eigenvalues greater than 1.00 and collectively explained 62.66% of the variance).  - Sensitivity analysis, using parallel analysis with polychoric correlations over 300 iterations, and scree plots, further confirmed the presence of the three-factor structure.  **CONSTRUCT VALIDITY:**   - ***Known-group validity***   - The older age group and those who reported that they appeared older than their actual age reported more everyday ageism than their younger and younger looking counterparts, respectively. This pattern was replicated in ageist messages (age group only), interpersonal interactions (both), and internalized ageism (relative appearance only). When stratified by age group, the three-factors structure was replicated for both the younger (60-64) and older (65-80) age groups. |
| **(NON)ESSENTIALIST BELIEFS ABOUT AGING ([N]EBA)** | *Weiss et al., 2016 | Longitudinal | United States and Germany | Adults and community-dwelling OA  *N:* Study 1 = 250; Study 2 = 103; Study 3 = 174  *Age:* Study 1:M = 42.21, SD = 19.46 (18-77); Study 2:M = 42.83, SD = 13.48 (20-77); Study 3:M = 55.58, SD = 4.69(34-67)  *Gender:* Study 1 = 75% F; Study 2 = 51% F; Study 3 = 60% F | *N° of items:* 4  *Dimension(s):* unidimensional; continuum of beliefs with malleable beliefs about aging in contradistinction to fixed beliefs about aging  *Response scale:* 6-point Likert scale (from “do not agree” to “absolutely agree”) | **INTERNAL CONSISTENCY:**  - Cronbach’s α was 0.73 (Weiss & Grah, 2014). | NA |
| (N)EBA | *Weiss et al., 2021 | Cross-sectional | United States and Germany | Adults and community-dwelling OA  *N:* Study 1 = 124;  Study 2 = 1,080  *Age:* Study 1: M = 63.73, SD = 8.28;  Study 2: M = 45.08, SD = 15.76  *Gender:* Study 1 = 77.4% F; Study 2 = 49.6% F | *N° of items:* Two versions*:*   - 4-item version (see Weiss et al., 2016) - 10-item version   *Dimension(s):* 2 dimensions: the beliefs that aging-related changes are relatively fixed (essentialism), the belief that aging-related changes are relatively malleable (nonessentialism).  *Response scale:* 7-point Likert scale (from “do not agree” to “absolutely agree”) | - *4-item version (See Weiss et al. 2016):*   **INTERNAL CONSISTENCY:**  - (N)EBA was measured at four occasions during the study and the internal consistencies (i.e., Cronbach’s α) were 0.72., 0.55, 0.64, and 0.68, respectively.  **RELIABILITY***:*   - ***Test-retest stability***   - Results suggested that (N)EBA remained quite consistent across time. The average intercorrelation between (N)EBA was r = 0.64 (p < .001) and the test–retest stability coefficients ranged from 0.58 to 0.76 across the four measurement occasions.   - *10-item version:*   **INTERNAL CONSISTENCY:**  Cronbach’s α of the entire aging essentialism scale was 0.81 (the  United States: 0.84; Germany: 0.72). | - *4-item version:*   **CONSTRUCT VALIDITY:**   - **DISCRIMINANT AND CONVERGENT VALIDITY:**   - Analyses supported the discriminant and convergent validity of the scale, as essentialist beliefs about aging were moderately associated with other aging attitude measures (AARC)   - *10-item version (See Weiss et al. 2016)*   **STRUCTURAL VALIDITY:**   - ***Confirmatory Factor Analysis***   - The CFA confirmed for the two-factorial model: χ^2^ = 283.79, df = 34, CFI = 0.94, RMSEA = 0.08, SRMR = 0.06, AIC = 37,25, BIC = 37,40.  **MEASUREMENT INVARIANCE:**   - ***Measurement invariance:***   - Age: model comparisons confirmed measurement invariance across age groups.  - Sex: significant mean differences at the factor score level between men and women such that men reported significantly higher levels of essentialist beliefs, while women reported significantly higher levels of nonessentialist beliefs.  - Countries: Essentialist and nonessentialist beliefs about aging were endorsed significantly more in Germany, when compared with the United States. |
| **Single-item scale**  **for assessing these (non)-essentialist beliefs about aging [(N)EBA-SIS]** | *Weiss et al., 2025 | Cross-sectional | Germany | Study 1  *N:* 168  *Age:* M= 63.24; SD= 5.88  *Gender:* 59% F  Study 2  *N:* 98 (hospitalized multimorbid patients)  *Age:* M=83.07, SD = 6.14  *Gender:* 75.5% F | *N° of items:* 1 item “What do you think: Is the way we age predetermined by genetics and immutable, or is it malleable and changeable?”  *Dimension(s):* unidimensional  *Response scale:* the scale was anchored from *“*Aging is genetically predetermined and immutable” (-50) to “Aging is malleable and changeable” (+50) | **Study 1**  **RELIABILITY***:*   - ***Test-retest stability***   - After four-month interval: r = 0.68 | **Study 1**  **CONSTRUCT VALIDITY:**   - ***Convergent validity***   - Signifincant correlations between [N]EBA-SIS with 10-item [N]EBA total score (r = 0.71), EBA subscale (r=0.46) and NEBA subscale (r= -0.62)   - ***Divergent Validity***   - No significant correlation with chronological age. No significant association with AARC-Gains, but positively with AARC-Losses (r_(164)_= 0.25). Correlations between [N]EBA-SIS and education (r_(213)_=- 0.24), life satisfaction( r_(218)_=- 0.18), subjective age bias (r_(218)_= 0.20).  **Study 2**  **CONSTRUCT VALIDITY:**   - ***Convergent validity***   - (N)EBA-SIS significantly correlated with (N)EBA scale (r(98) = 0.45   - ***Divergent Validity***   - NEBA-SIS significantly correlated with the perception of being younger or older than one’s chronological age (r = 0.25)  - No significant correlations with demographic factors (e.g., age, gender, education level), geriatric assessments (e.g., Barthel Index, Lachs geriatric screening, MMSE, Geriatric Depression Scale), or life satisfaction. |
| **EXPECTATIONS REGARDING AGING SURVEY (ERA-38)** | Sarkisian et al. 2001 | Cross-sectional | United States | Adults and community-dwelling OA  *N:* OA = 49; primary care clinicians = 11  *Age:* older adults: M = 78 (65-91); primary care clinicians: M = 37 (30-51)  *Gender:* OA = 34.2% F; primary care clinicians = 54.5% F |  | See Sarkisian et al. 2002 | **CONTENT VALIDITY:**  - Focus group to determine which domains of expectations regarding aging. Each domain was ranked by the frequency of mention by all participants, and separately by older adults and by physicians. Qualitative content analysis of the focus group transcripts identified 26 domains of expectations regarding aging. |
| 38-ERA | Sarkisian et al. 2002 | Cross-sectional | United States | Community-dwelling OA  *N:* 429  *Age:* M = 76  *Gender:* 54% F | *Dimension(s):* total score and 10 dimensions (i.e., general health, cognitive function, mental health, functional independence, sexual function, pain, sleep, fatigue, urinary incontinence, appearance)  *Response scale:* 4-point Likert scale (from “definitely false” to “definitely true”) | **INTERNAL CONSISTENCY:**  - With the exception of the pain scale (Cronbach’s α = 0.58), internal consistency reliability exceeded 0.73 for all scales. Item discrimination rates ranged from 44% (pain) to 100% (sexual function and sleep); again, the pain scale’s poor performance was an outlier, as all other scales had item discrimination rates greater than or equal to 80%. | **CONSTRUCT VALIDITY:**   - ***Convergent validity***   ***Correlations*:**  - 38-ERA scores correlated moderately with Physical Component Summary (PCS-12) and Mental Component Summary (MCS-12) scores and inversely with Geriatric Depression Scale (GDS) scores and age. |
| 12-ERA | Nia et al., 2023 | Cross-sectional | Iran | Community-dwelling OA  *N:* 400  *Age:* M = 71.32, SD = 6.09  *Gender:* 39.5% F | *N° of items:* 12  *Dimension(s):* 3 dimensions (stereotypes [cognitive component], prejudice [emotional component], discrimination [behavioral component])  *Response scale:* 4-point Likert scale (1 = “definitely false”, 2 = “somewhat false”, 3 = “somewhat true”, 4 = “definitely true”) | **INTERNAL CONSISTENCY:**  - Cronbach’s α (ranging from 0.79 to 0.85), McDonald’s omega (ranging from 0.79 to 0.85), CR (ranging from 0.77 to 0.85), and MaxR (ranging from 0.78 to 0.86) were all greater than 0.7. | **CONTENT VALIDITY:**  **-** 12-ERA survey was reviewed by 15 experts for feedback on wording, grammar, item allocation, and scaling. Content validity was assessed using the Content Validity Ratio (CVR) and Content Validity Index (CVI). The CVR for all items was above 0.49. Additionally, the modified kappa coefficient (K) was calculated, with each item’s K value exceeding 0.6, confirming the appropriateness of the items.  **STRUCTURAL VALIDITY:**   - ***Exploratory Factor Analysis***   - Maximum likelihood EFA with Varimax rotation was conducted. The extracted three factors explained 65.764% of the total variance, comprising 11 items.   - ***Confirmatory Factor Analysis***   - Maximum likelihood CFA confirmed the obtained factor structure. The results showed that after reviewing the model modification indices, the revised three factors model fits the data well, as evidenced by goodness-of-fit indices (χ^2^ = 94.516, df = 49, p < .05, χ^2^/df= 1.93, CFI = 0.95, NFI = 0.90, RMSEA = 0.07)  **CONSTRUCT VALIDITY**:   - ***Convergent validity***   - AVE should be greater than 0.5, and CR should be greater than 0.7. The results indicated that AVE of physical health was more than 0.5 (0.50), but AVE of mental health (0.45) and cognitive function (0.50) were slightly less than 0.5. It is argued that AVE is a conservative and strict measure of convergent validity, and convergent validity can be achieved on the basis of CR alone. Therefore, this study established convergent validity referring to the CR of greater than 0.7. |
| 12-ERA | Beser et al., 2012 | Cross-sectional | Turkey | Community-dwelling OA  *N:* 120  *Age:* M = 71.68; SD = 5.37 (65-86)  *Gender:* 54% F | *-* | **INTERNAL CONSISTENCY:**  - Cronbach’s α coefficient was found to be 0.76 for the total scale. For the subscales, it was 0.51 for the physical health subscale, 0.48 for the mental health subscale, and 0.75 for the cognitive function subscale. The item-total score correlations ranged from 0.36 to 0.69, and these correlations were statistically significant. | **STRUCTURAL VALIDITY:**   - ***Confirmatory Factor Analysis***   - CFA confirmed that the three-factor model had a good fit: χ^2^ = 80.51, p < .10, RMSEA = 0.06, CFI = 0.94, SRMR = 0.08, NNFI = 0.92. |
| **IMAGE OF AGING SCALE** | Levy et al., 2004 | Cross-sectional | United States | Community-dwelling OA  *N:* Sample 1 = 20; Sample 2 = 68; Sample 3 = 76  *Age:* Sample 1: M = 66.4, SD = 10.9; Sample 2: M = 69.1, SD = 12; Sample 3: M = 73, SD = 8.7  *Gender:* Sample 1 = 40% F; Sample 2 = 44,1% F; Sample 3 = NA | *N° of items:* 18  *Dimension(s):* 2 dimensions (positive age-stereotype component, negative age-stereotype component)  *Response scale:* 5-point Likert scale: (1) how characteristic of old people the item was considered to be, with anchors of 1 = not at all characteristic and 5 = very characteristic, and (2) how positive or negative the item was considered to be, with anchors of 1 = very negative and 5 = very positive | **INTERNAL CONSISTENCY:**  - The Cronbach’s α for the positive age-stereotype component and negative age-stereotype component were 0.84 and 0.82, respectively.  **RELIABILITY**:   - ***Test-retest stability***   - The test-retest reliability of the positive age-stereotype component was found to be 0.92 over a week, while the negative age-stereotype component had a test-retest reliability of 0.79. | **CONSTRUCT VALIDITY:**   - ***Convergent validity***   ***Correlations*:**  - The convergent validity of the positive age-stereotype component was supported by its correlation of .83 with the positivity rating of the open-ended measure.  - The negative age-stereotype component of the scale was found to be associated with higher lifetime exposure to television, even after controlling for age, depression, education, and self-rated health. |
| 14-IAS | Bai et al., 2012 | Cross-sectional | China | Community-dwelling OA  *N:* 445  *Age:* 60-69; 70-79; 80+  *Gender:* 49.7% F | *N° of items:* 14 | **INTERNAL CONSISTENCY:**  - Internal consistency reliability of the five subscales of IAS was acceptable with Cronbach’s α coefficient ranging from 0.51 to 0.65 (total = 0.73) and Guttman split-half ranging from 0.42 to 0.57. The corrected item-total correlations for each item were almost all in a moderate range.  **RELIABILITY**:   - ***Test-retest stability***   - The overall test-retest reliability reached 0.87. | **STRUCURAL VALIDITY:**   - ***Exploratory Factor Analysis***   - EFA revealed a five-factor solution with 14 item which accounted for 60.2% of the total variance.   - ***Confirmatory Factor Analysis***   - The result showed an acceptable overall fit of the five-factor model compared with either the nine-factor model or the two-factor model that had been suggested by the original scale development study: χ^2^ = 75.29, df = 65, p = 0.18, CFI = 0.97, TLI = 0.96, and RMSEA = 0.03.  **CONSTRUCT VALIDITY:**   - ***Convergent validity***   ***Correlations:***  - The overall IAS scores were correlated significantly with greater interest in carrying out more daily activities (r = 0.29, p < .001) and were negatively correlated with number of chronic diseases (r = –0.34, p < .001). It also predicted higher levels of life satisfaction (r = 0.27, p < .001). The five subscale scores were also found to have positive associations with life satisfaction, and daily activity levels, while they have negative associations with the number of chronic diseases. |
| **KNOWLEDGE OF MEMORY AGING QUESTIONNAIRE (KMAQ)** | *Cherry et al., 2000 | Cross-sectional | United States | Adults and community-dwelling OA  *N:* Sample 1 = 143; Sample 2 = 85; Sample 3 = 32; Sample 4 = 46  *Age:* Sample 1: M = 21.8, SD = 5.1; Sample 2:  M = 73.1, SD = 5.5; Sample 3: M = 25.2, SD = 7.6; Sample 4: M = 23.05, SD = 3.59  *Gender:* NA | *N° of items:* 28  *Dimension(s):* 2 dimensions (normal memory aging, pathological memory aging)  *Response scale*: dichotomous response (true/false) | **INTERNAL CONSISTENCY:**  - The KMAQ demonstrated an internal consistency reliability with a Cronbach’s α coefficient of 0.55. | **CONTENT VALIDITY:**  - 20 experts assessed the content validity of an initial 34-item version. Reviewer comments that indicated subjectivity, poor working, or ambiguity were the primary criteria for removal of items. Taking into consideration reviewer comments and item-difficulty indices, 6 items were eliminated.  **STRUCTURAL VALIDITY:**   - ***Item Response Theory***   - The initial 34-item version was administered to undergraduate students. On the basis of their performance, item difficulty indices were calculated (i.e., the proportion of participants answering each item correctly).  **CONSTRUCT VALIDITY*:***   - ***Convergent validity***   ***Correlations:***  -. Significant associations between KMAQ pathological memory aging and FAMHQ. No correlation between ADK and KMAQ.   - ***Known-group validity***   ***ANOVA:***  - Mental health professionals were more accurate than undergraduate students. Older adults were more knowledgeable on issues related to pathological memory aging compared with normal memory aging, as was also the case for mental health professionals.  **RESPONSIVENESS**:   - ***ANOVA***:   - The KMAQ is sensitive to instruction, as shown in the third study where students were part of a series of lectures about normal and pathological changes in memory. |
| **MULTIDIMENSIONAL SCALE FOR THE ASSESSMENT OF THE SALIENCE OF AGE IN SOCIAL INTERACTION** | *Kruse & Schmitt, 2006 | Cross-sectional | Germany | Adults and community-dwelling OA  *N:* 1,275  *Age:*45-75  *Gender:* 56.8% F | *N° of items:* 24  *Dimension(s):* 5 dimensions (developmental gains and chances for development, developmental losses and risks of development, older people as a burden on society, age salience)  *Response scale:* 4-point Likert scale (from “completely disagree” to “completely agree”) | **INTERNAL CONSISTENCY:**  - Cronbach’s α was adequate for all subscales, ranging from 0.62 to 0.72. | **STRUCTURAL VALIDITY:**   - ***Exploratory Factor Analysis***   - PCA with direct Oblimin rotation was performed, revealing a five-factor structure of variations in agreement with the age-stereotyping statements.  - The factor loadings of the items on the corresponding factors ranged from 0.39 to 0.77, and no item had a substantial loading (>0.30) on a non-corresponding factor.  - The low inter-correlations between the five subscales indicate that the four age stereotypes and age salience represent independent dimensions of the social cognition of aging and older people.  **CONSTRUCT VALIDITY:**   - ***Known-group validity***   ***General linear model:***  - The main effects of age group were that it significantly predicted age salience, older people as a burden on society, and the perception ofold age as a life phase of developmental gains and potential. |
| **RELATING TO OLD PEOPLE EVALUATION (ROPE)** | *Cherry & Palmore, 2008 | Cross-sectional | United States | Adults and community-dwelling OA  *N:* 314 (College students = 147; Older adults = 120; University community = 47)  *Age:* College students: M = 22.9, SD = 5.6; Older adults: M = 70.9, SD = 10.0; University community: M = 38.0, SD = 16.7  *Gender:* 68.5% (College students = 74,1%; Older adults = 62.5%; University community = 66%) | *N° of items:* 20  *Dimension(s):* total score, and 2 dimensions (positive types of ageism, negative types of ageism)  *Response scale:* 3-point Likert scale (never, sometimes, often) | **INTERNAL CONSISTENCY:**  - The estimated Cronbach’s α value was 0.70, which is considered adequate. However, it is slightly lower than the standard convention of 0.80.  **RELIABILITY:**   - ***Test-retest stability***   - Pearson's product-moment correlations were calculated to assess the test-retest reliability (N = 90). The correlations between the first and second administration were found to be 0.57 for positive ageism items and 0.72 for negative items. These values indicate a moderate to strong level of stability over time. | **CONTENT VALIDITY:**   - ***Face validity***   - The items were considered to have high face validity, as all respondents appeared to understand the items without further explanation. |
| **AGEISM SURVEY (AS)** | Palmore, 2001 | Cross-sectional | United States | Community-dwelling OA  *N:* 84  *Age:* M = 75 (60-93)  *Gender:* 65% F | *N° of items:* 20  *Dimension(s):* unidimensional (total score)  *Response scale:* 3-point option (“Never”, “Once”, “More than once”) | **INTERNAL CONSISTENCY:**  - The Cronbach’s α was found to be 0.81 | **CONTENT VALIDITY:**   - ***Face validity***   - A panel consisting of older individuals and colleagues were asked to provide their interpretation of each item.  **STRUCTURAL VALIDITY:**   - ***Exploratory Factor Analysis***   - The EFA revealed one main factor with an Eigenvalue of 4.74, suggesting that most of the variance in the data can be explained by this single factor.  **CONSTRUCT VALIDITY:**   - ***Known-group validity***   ***χ^2^ test and Wilcoxon test:***  - No difference between age, gender, and education in the frequency of item reported. |
| AS | Erol et al., 2016 | Cross-sectional | Turkey | Community-dwelling OA  *N:* 236  *Age: M =* 64.44, SD = 9.57 (50-95)  *Gender:* 69.1% F | *-* | **INTERNAL CONSISTENCY:**  - Cronbach’s α for the Turkish ageism survey showed good internal consistency at 0.86. The item-total correlation results indicated that the correlation values for all of the items in the survey were 0.31. | **CONTENT VALIDITY:**  - The Turkish and English versions of the instrument were reviewed by an expert panel of seven university faculty members, including public health physicians and nurses. Experts rated the items based on the CVI (1 = unsatisfactory, 4 = very satisfactory). To be considered 80% valid, items had to receive a score of 3 or 4. The item-level CVI for the Turkish version ranged from 0.85 to 1.00, and the scale-level CVI was 0.98.  **STRUCTURAL VALIDITY:**   - ***Exploratory Factor Analysis***   - EFA showed that the survey could be categorized into five dimensions. However, due to lack of meaningful subdimension cohesion, the survey was assessed as a single dimension. |
| 11-AS | Nia et al., 2021 | Cross-sectional | Iran | Community-dwelling OA  *N:* 400  *Age:* M = 71.32, SD = 6.09  *Gender:* 39.5% F | *N° of items:* 11  *Dimension(s):* three subscales (Age-related deprivation, Dignity, Employment)  *Response scale:* 3-point option (“Never”, “Once”, “More than once”) | **INTERNAL CONSISTENCY:**  - Cronbach’s α were adequate for the three factors (Age-related deprivation= 0.73, Dignity = 0.70, Employment= 0.71).  - The CR for all factors was between 0.70 and 0.73, and the MaxR was between 0.70 and 0.74, suggesting that the construct reliability was sufficient for all factors. | **CONTENT VALIDITY:**   - ***Content validity***   - Based on the response from 15 experts, CVR of all 20 items was higher than the minimum threshold of 0.49. The results showed that the modified K coefficient value for each item of the Persian version of the ageism survey was higher than 0.6.  **STRUCTURAL VALIDITY:**   - ***Exploratory Factor Analysis***   - EFA with Promax rotation revealed a three-factors structure: age-related deprivation with five items, dignity with three items, and employment with three items.   - ***Confirmatory Factor Analysis***   - The CFA revealed that the three-factor structure fitted the model well (χ^2^ = 75.79, df = 41, p < .05, GFI = 0.97, CFI = 0.97, NFI = 0.93, IFI = 0.97, RFI = 0.91, TLI = 0.96, SRMR = 0.04, RMSEA = 0.05).  **MEASUREMENT INVARIANCE:**  - A few invariance models were created to detect whether the Persian version of the ageism survey model holds in both male and female groups. Using the absolute value of ΔCFI < 0.01 and ΔRMSEA < 0.01 criteria, both metric and scalar invariance were found between male and female groups.  **CONSTRUCT VALIDITY:**   - ***Convergent validity***   - Although the AVE for all factors is < 0.5, it is close to the threshold. The study’s findings showed that the AVE for each construct is less than its CR, and the CR for all factors is > 0.7. |
| **VIEWS ON AGING SCALES (VAS)** | *Kornadt et al. (2011) | Cross-sectional | Germany | Adults  *N:* Convenience sample = 14; Sample *=* 700  *Age*: Convenience sample: M = 73(56-87); Sample: M = 54.37, SD = 14.55  *Gender:* Convenience sample = NA; Sample = 49.9% F | *N° of items:* 27 bipolar statements  *Dimension(s):* 8 dimensions (“family and partnership”, “friends and acquaintances”, “religion and spirituality”, “leisure activities and social or civic commitment”, “personality and way of living”, “financial situation and dealing with money-related issues”, “work and employment, physical and mental fitness, health, and appearance”)  *Response scale:* 8-points bipolar scale (choosing between opposite poles of each stereotypical belief about age [i.e. each statement]) | **INTERNAL CONSISTENCY**:  - Cronbach’s α ranged from good (0.67 for financial situation and dealing with money-related issues domain) to very good (0.86 for leisure activities and social or civic commitment domain) with a mean alpha of = 0.78  - The corrected item-total correlations, as well as those for all subdomains, were found to be acceptable. | **STRUCTURAL VALIDITY:**   - ***Exploratory Factor Analysis***   - PAF with Varimax rotation was performed on the 27 items that represent evaluations of "old persons" across various life domains.  - The Scree Plot was examined to determine the number of factors to retain, and it suggested an eight-factor solution. The eigenvalues of all eight factors were greater than 1, collectively explaining 68% of the total variance in the data.  **CONSTRUCT VALIDITY:**   - ***Convergent validity***   ***Correlations:***  - Correlation between the stereotype ratings and life satisfaction ratings across the eight life domains for each participant were computed. Results indicated that with increasing age, domain-specific age stereotypes become more predictive for domain-specific life satisfaction. |
| Brief- Scales on Views on Aging | Kornadt et al., 2018 | Cross-sectional | Germany | Adults and community-dwelling OA  *N:* 301  *Age:* M = 50.56, SD = 15.72 (23-88)  *Gender:* 49.8% F | *N° of items:* 18  *Dimension(s):* 9 dimensions (“family,” “friendships,” “leisure,”, “personality,” “finances,” “work,” “appearance,” “fitness,” “health”)  *Response scale:* 8-points bipolar scale (choosing between opposite poles of each stereotypical belief about age [i.e. each statement]) | NA | **CONSTRUCT VALIDITY:**   - ***Convergent validity***   ***Correlations:***  - Correlations between constructs for domains that matched in content (i.e., correlations within the same domain) and those where constructs from different domains were correlated. |
| **PERSONAL VOA** | | | | | | | |
| **ANXIETY ABOUT AGING SCALE (AAS)** | *Lasher & Faulkender, 1993 | Cross-sectional | United States | Adults and community-dwelling OA  *N:* 312  *Age:* 25-34; 35-44; 45-64; 65-74; 75+  *Gender:* 58.3% F | *N° of items:* 20  *Dimension(s):* 4 dimensions (fear of old people, psychological concerns, physical appearance, fear of loss)  *Response scale:* 5-point Likert scale (from “strongly disagree” to 5 “strongly agree”) | **INTERNAL CONSISTENCY:**  - Cronbach’s α coefficient for the total twenty-item AAS was 0.82. Fear of old people = 0.78, psychological concerns = 0.74, physical appearance = 0.71, fear of loss = 0.69. | **STRUCTURAL VALIDITY:**   - ***Exploratory Factor Analysis***   - PCA with direct Oblimin rotation (gamma = 0) was used. After several solutions, 20 items were retained, explaining 50.60% of the variance.  **CONSTRUCT VALIDITY:**   - ***Known-group validity***   ***Correlations*:**  - Intercorrelations between the four AAS subscales, total AAS score, and demographic variables showed that being male, having low or poor-quality contact with the elderly, was associated with higher fear of aging. Poor-quality contact correlated with higher anxiety about psychological concerns, while physical appearance was linked to age. Additionally, male participants with less and poorer-quality contact with elderly individuals were more likely to fear loss. |
| AAS | Pakpour et al., 2021 | Cross-sectional | Iran | Community-dwelling OA  *N:* 703  *Age:* M = 69.41, SD = 8.  *Gender:* 40.8% F | *-* | **INTERNAL CONSISTENCY:**  - All Cronbach’s α (ranging from 0.70 to 0.88) values were above the acceptable thresholds in four subscales.  - The corrected item-total correlations exceeded the recommended threshold of a value of 0.40, ranging from 0.40–0.80. | **CONTENT VALIDITY:**  - Expert committee: All translators, the project manager, two health education specialists, and two geriatricians evaluated the AAS's cross-cultural face validity, making modifications for cultural adaptability and addressing translation issues.  - Piloting: The Iranian (Persian/Farsi) AAS was tested on 10 older adults to gather feedback on the clarity and understandability of the items.  **STRUCTURAL VALIDITY:**   - ***Confirmatory Factor Analysis***   - Applying CFA indicated that the model’s four original factors are the best solution, representing 55% of the total variance. The result of the CFA showed that this four-factor model had a good fit for the data: χ^2^ = 593.84, df = 164, p < 001, CFI = 0.93, TLI = 0.91, RMSEA = 0.08, SRMR = 0.07.   - ***Item Response Theory***   - Rasch analyses using the partial credit model showed that item fit was within the acceptable range for both Infit and Outfit MnSq (0.5–1.5). The item separation index and reliability were above the acceptable thresholds (>2 and >0.7, respectively).  **MEASUREMENT INVARIANCE**:   - ***Item Response Theory***   - DIF analysis across gender and living status showed all contrasts were < 0.5 logits, indicating invariance across these groups.  **CONSTRUCT VALIDITY:**   - ***Convergent validity***   - AVE and CR were computed using the factor loadings extracted from the CFA. CR (ranging from 0.79 to 0.90), and AVE (ranging from 0.43 to 0.63) values were above the acceptable thresholds in four subscales.   - ***Known-group validity***   ***T-test and ANOVA:***  - There was no significant correlation between gender (p = 0.31), educational level (p = 0.52), and marital status (p = 0.86) with a mean score of anxiety of aging. |
| 12-AAS | Sargent-Cox et al., 2013 | Cross-sectional | Australia | Adults and community-dwelling OA  *N:* 783  *Age:* M = 57.3, SD = 13.66 (20-97)  *Gender:* 42% F | *N° of items:* 12 | **INTERNAL CONSISTENCY:**  - Internal consistency for the four factors, as measured by Cronbach’s α, was good for fear of old people (α = 0.80), psychological concerns (α = 0.80), and physical appearance (α = 0.73) dimensions, and moderate for fear of loss factor (α = 0.69). | **STRUCTURAL VALIDITY:**   - ***Confirmatory Factor Analysis***   - CFA value of the a priori first-order four-factor AAS model indicated reasonable fit on most goodness of fit indices: χ^2^ = 491, df = 164, p < 001, CFI = 0.91, RMSEA = 0.05.  **MEASUREMENT INVARIANCE**:   - ***Multiple group analysis***   - MGA was used to test for gender and age measurement invariance in the four-factor AAS model. Measurement invariance for both gender and age groups (young adults 20–44 years; mid-aged adults 45–64 years, older adults 65+ years old) was found for three of the factors, but not for all items in the fear of loss factor. |
| 12-AAS | Zueck-Enríquez et al., 2021 | Cross-sectional | Mexico | Community-dwelling OA  *N:* 601  *Age:* M = 70.69, SD = 8.10 (60-90)  *Gender:* 65.6% F | *-* | **INTERNAL CONSISTENCY:**  - The reliability of each dimension of the tested models was calculated using  Cronbach’s α and the omega coefficient. The factors obtained in the confirmatory factor analyses in both models, except for the psychological concerns factor in the AAE-4F model, reach internal consistency values above 0.70. | **STRUCTURAL VALIDITY:**   - ***Confirmatory Factor Analysis***   - CFA confirmed the four-dimensional structure. The overall results of the confirmatory factor analysis (χ^2^ = 122.26, GFI = 0.97; RMSEA = 0.05;CFI = 0.98) of the second and last model tested (AAE-4Fm) corresponding to the four-dimensional structure of the previous model without items 2, 3, 4, 5, 6, 7, 16 and 19, which were not sufficiently well explained by the original model or that according to the modification indices were not adequate, indicate that this measurement model is better than the previous model and its fit is optimal.  **MEASUREMENT INVARIANCE:**  - The factor loadings and the intercepts are considered invariant in the two populations (men and women). Strong factorial invariance: χ^2^ = 249.26, df = 114, GFI = 0.93. NFI = 0.92, CFI = 0.96, RMSEA = 0.05, AIC = 333.26. However, there are differences between populations on the means of physical appearance and fear of loss factors.  **CONSTRUCT VALIDITY**:   - ***Discriminant validity***   ***Correlations:***  - Low to moderate intercorrelations between the factors are observed, showing an adequate discriminant validity between them. |
| AAS | Pifer & Segal, 2024 | Cross-sectional | United States | Community-dwelling OA  *N:* 151  *Age:* M = 66.21, SD = 5.11 (60-84)  *Gender:* 59.6% F |  | **INTERNAL CONSISTENCY:**  - AAS total score (α=.92) and subscales: Fear of Old People (α=.83), Psychological Concerns (α=.85), Physiological Concerns (α=.87), and Fear of Losses (α=.83). | **STRUCTURAL VALIDITY:**   - ***Confirmatory Factor Analysis***   - CFA confirmed 4-factor structure: χ2<.05, CFI <.90, and RMSEA was >.08  **CONSTRUCT VALIDITY:**   - ***Convergent* validity**   - Correlations between AAS total score with ageism (Fraboni Scale of Ageism; r=−0.51, p < .01), expectations regarding aging (ERA; r =−0.60, p < .01), and death anxiety (Templer Death Anxiety Scale; r=−0.65, p < .01). |
| **AGING PERCEPTIONS QUESTIONNAIRE (APQ)** | *Barker et al., 2007 | Cross-sectional | Ireland | Community-dwelling OA  *N:* 2,033  *Age:* M = 74.1, SD = 6.8  *Gender:* 57% F | *N° of items:* 32  *Dimension(s):* 7 dimensions (timeline chronic, timeline cyclical, consequences positive, consequences negative, control positive, control negative, emotional representations)  *Response scale:* 5-point Likert scale (from “strongly disagree” to “strongly agree”) | **INTERNAL CONSISTENCY:**  - Cronbach’s α coefficients for the subscales were above 0.70, indicating good internal reliability. In most cases, the coefficients exceeded 0.80, further supporting the reliability of the instrument. | **STRUCTURAL VALIDITY:**   - ***Confirmatory Factor Analysis***   - CFA results indicated that the model had a good fit, as evidenced by the chi-square value (χ^2^ = 2788, df = 496), and fit indices (CFI = 0.91, IFI = 0.91, GFI = 0.91, RMSEA = 0.05, p < .001). This suggests that each subscale of the instrument addresses a unique dimension of an individual's experience of aging.   - ***Mokken scale analysis:***   - Mokken scale analysis revealed strong scalability based on the Loevinger coefficient of H. The following subscales demonstrated high scalability: timeline chronic (H = 0.62), timeline cyclical (H = 0.65), consequences negative (H = 0.56), control positive (H = 0.48), and emotional representations (H = 0.55). However, for the control negative subscale, one item did not meet the scalability criterion and was excluded from further analysis. |
| APQ | Chen et al., 2015 | Cross-sectional | China | Community-dwelling OA  *N:* Pilot Sample = 94; EFA sample = 379; CFA sample = 379  *Age:* Pilot Sample: M = 71.80, SD = 6.48; EFA sample: M = 72.38, SD = 6.54: CFA sample: M = 73.75, SD = 6.7  *Gender:* Pilot Sample = 53.2% F; EFA sample = 52.5% F; CFA sample = 53.3% F | *-* | **INTERNAL CONSISTENCY:**  - Cronbach’s α were 0.88 for the total scale, 0.87 for timeline acute/chronic, 0.70 for timeline cyclical, 0.84 for emotional representations, 0.82 for control positive, 0.75 for control negative, 0.67 for consequences positive, 0.84 for consequences negative, indicating acceptable internal consistency reliability.  **RELIABILITY:**   - ***Test-retest stability***   - ICC were all greater than 0.8, indicating good stability for each item of the C-APQ. | **CONTENT VALIDITY:**   - ***Content validity index:***   - CVI of C-APQ was assessed by each expert rating each item on a 5-point Likert scale (1 = ‘strongly disagree’ to 5 = ‘strongly agree’) for equivalence of translation. All of the computed ICVI values were greater than 0.7, and most of them were greater than 0.90, indicating adequate content validity.  **STRUCTURAL VALIDITY**   - ***Exploratory Factor Analysis***   - EFA revealed 7 eigenvalues greater than 1, which accounted for 61.6% of variance, with most items loading above 0.40.   - ***Confirmatory Factor Analysis***   - A CFA model with 7 factors failed to perform exact fit (χ^2^/df = 2.03, p <.001). An acceptable fit was indicated by RMSEA = 0.05, NNFI = 0.91, IFI = 0.92 and CFI = 0.92. The result of the CFA supported the multidimensional nature of aging experience, with seven factors structure very similar to the result of the original version from Irish population. |
| APQ | Ingrand et al., 2012 | Cross-sectional | France | Community-dwelling OA  *N*: 656 (Sample 1 = 262; Sample 2 = 394)  *Age*: Sample 1: M = 59.8, SD = 2.8 (54–64); Sample 2: M = 77.4, SD = 8.2 (65–106)  *Gender*: Sample 1 = 53.4% F; Sample 2 = 58.4% F | *-* | **INTERNAL CONSISTENCY:**  - Cronbach’s α ranged from 0.52 to 0.86. | **STRUCTURAL VALIDITY*:***   - ***Confirmatory Factor Analysis***   - CFA model with 7 factors failed to perform exact fit in the older sample (χ^2^ = 1204.6, df = 443, p < .001). An acceptable fit was indicated by RMSEA) 0.07 whereas the CFI value (0.93) was slightly below the suggested cut-off. The same seven-factor a-priori-defined model yielded a similar, but slightly better fit in the 55–64 as in the older group (RMSEA = .07; CFI = .95) but also failed to perform exact fit (χ² = 953.3 df = 443 p < .001).  **MEASUREMENT INVARIANCE:**   - ***Multigroup Confirmatory Factor Analysis****:*   ***-*** χ^2^ test rejected measurement invariance in relation with differences in some parameters of the model. However, the comparison of model fit with ΔCFI = 0.00 compared to a cut-off value of 0.02 was good evidence in favor of approximate factorial invariance between age groups. |
| Brief-APQ | Miremadi et al., 2020 | Cross-sectional | Iran | Community-dwelling OA  *N:* 500  *Age:* 60–80+  *Gender:* 52.2% F | *N° of items:* 20  *Dimension(s): 4* dimensions (consequences negative, emotional representations, control positive, consequences positive)  *Response scale:* 5-point Likert scale (from “strongly disagree” to “strongly agree”) | **INTERNAL CONSISTENCY:**  - Cronbach’s α was estimated first for the whole questionnaire and then for each extracted factor.  Finally, Cronbach’s α of 0.83 and ranging from 0.80 to 0.87 were found for the whole scale and the subscales, respectively.  **RELIABILTY:**   - ***Test-retest stability***   - Stability was assessed using the ICC. An ICC of 0.96 ranging from 0.90 to 0.98 were found for the whole scale and the subscales, respectively and construct reliability statistic (0.86, 0.83, 0.88 and 0·83 respectively) of the APQ in the four extracted factors in the present study was estimated to be desirable (> 0.7) | **STRUCTURAL VALIDITY:**   - ***Exploratory Factor Analysis***   - The latent factors were extracted using the maximum likelihood estimation and the Varimax rotation. In the model, four factors capable of explaining 52.8% of variance were extracted, based on eigenvalues above 1 and scree plots.   - ***Confirmatory Factor Analysis***   - After reviewing model misfit, the single factor consisting of 19 items with good fit to the data was achieved. The fit indices were as follows: χ^2^ = 258.05, df = 145, CMIN/df = 1.78, RMSEA = 0.04, PCFI = 0.82, PNFI = 0.79, AGFI = 0.93, IFI = 0.97, CFI = 0.97.  **CONSTRUCT VALIDITY:**   - **Convergent and discriminant validity**   - Factor AVE (0.54, 0.52, 0.70 and 0·638 respectively) was greater than MSV (0.33, 0.33, 0.10 and 0.10 respectively) and ASV (0.11, 0.11, 0.03 and 0.03 respectively) |
| APQ | Slotman et al., 2015 | Cross-sectional | Netherlands | Community-dwelling OA  *N:* 1,280  *Age: M =* 78.59, SD = 6.17  *Gender:* 57.6% F |  | **INTERNAL CONSISTENCY:**  - Cronbach’s of the APQ dimensions ranged from 0.74 to 0.86, indicating either acceptable (α ≥ 0.70) or good (α ≥ 0.80) reliability of the dimensions  **SHORT VERSION**  **INTERNAL CONSISTENCY:**  **-** All Cronbach’s α were above 0.70, except for the control positive dimension (0.69). | **STRUCTURAL VALIDITY:**   - ***Confirmatory Factor Analysis***   - CFA confirmed a 7 factors structure (χ^2^ = 2043.91, df = 443, p < .001). SRMR and RMSEA indicated good fit (SRMR= 0.06; RMSEA = 0.05). CFI was slightly below the cut-off o 0.90 (CFI = 0.88).  **CRITERION VALIDITY**:   - ***Inter-factor and inter-instruments correlations***   - Correlation between factors similar to those present in the original version of the instrument and none exceed the critical point of 0.80. Most remarkable difference was the inter-factor correlations for the consequence positive dimension that were, for all but the control positive dimension, in the opposite direction.  **CONSTRUCT VALIDITY**:   - ***Convergent validity***   ***Stepwise regression analyses***  - APQ dimensions predict wellbeing except timeline chronic dimension and emotional representations dimensions, and physical functioning, except consequence positive dimensions and control negative dimensions.  **- SHORT VERSION**  **STURCTURAL VALIDITY*:***   - ***Item reduction analysis***   - Item reduction was performed in order to create a shorter version of the APQ, referred to as APQ-S . After item reduction, model fit statistics of the 21-item APQ-S indicated appropriate goodness of fit: χ2(168) = 589.80, p < .001; RMSEA = 0.05; SRMR = 0.04; CFI = 0.94.  **CRITERION VALIDITY**:   - ***Inter-factor and inter-instruments correlations***   - Correlations were similar to those found with the long versions of the instrument.  **CONSTRUCT VALIDITY**:   - ***Convergent validity***   ***Regressions:***  - APQ-S regressions coefficients were largely similar to the coefficients found when regressing APQ dimensions on wellbeing and physical functioning.   - ***Known-group validity***   ***Correlations***:  - Older age correlated with greater chronic and cyclical awareness of aging, more negative consequences and emotions, and less control over negative events. Men and less educated individuals showed more chronic awareness, while cyclical awareness was stronger among the single or widowed. Women saw more positive effects of aging, whereas single/widowed, low-educated, and Dutch respondents perceived more negative effects of aging. Lower education and income were linked to stronger negative emotions. Non-Dutch older adults, as well as those married or highly educated, felt more control over negative events. |
| Brief-APQ | Sexton et al., 2014 | Cross-sectional | Ireland | Community-dwelling OA  *N:* 6,718  *Age: Median = 62, IQR =* 56-70  *Gender:* 54% F | *N° of items:* 17  *Dimension(s):* 5 dimensions (timeline chronic, consequences positive, emotional representations, consequences and control negative, control positive)  *Response scale:* 5-point Likert scale (from “strongly disagree” to “strongly agree”) | **INTERNAL CONSISTENCY:**  - Cronbach’s was >0.7 for all sub-scales in the shortened version, indicating that internal consistency was preserved**.** | **STRUCTURAL VALIDITY:**   - ***Confirmatory Factor Analysis*:**   **-** The timeline cyclical dimension was removed because of weak empirical evidence. Consequences and control negative dimensions were combined in order to achieve good fit. The final five-factors model displayed excellent fit (χ^2^ = 1433.54, df = 109, p < .01, RMSEA = 0.04, CFI = 0.97, TLI = 0.96.  **CRITERION VALIDITY:**  ***Correlations*:**  - Correlations between APQ dimensions were largely preserved in the shortened version, with significant associations in the same direction and similar magnitude of the original study.  **CONSTRUCT VALIDITY:**   - ***Convergent and divergent validity***   ***Regressions:***  **-** Associations with relevant theoretical constructs (such as physical limitations, depression, and quality of life) were also preserved in the shorter version of the scale. |
| Brief-APQ | Jaafar et al., 2018 | Cross-sectional | Malaysia | Community-dwelling OA  *N:* 91  *Age:* M *=* 60.48, SD = 7.2  *Gender:* 59.3% F | *-* | **INTERNAL CONSISTENCY:**  - Cronbach’s showed good internal consistency for each of the domains except for emotional representation (0.60) and item-total correlations within each domain were all strong (> 0.30).  **RELIABILITY:**   - ***Test-retest stability***   - The test retest showed good agreement (ICC = 0.74). | **STRUCTURAL VALIDITY:**   - ***Confirmatory Factor Analysis***   - CFA confirmed a good model fit of the original B-APQ model (five factors) to the data (χ^2^ = 173.7, df = 109, p < .01, RMSEA = 0.04, CFI = 0.96).  **CONSTRUCT VALIDITY**:   - ***Convergent and divergent validity***   ***Correlations:***  - The shortened version presented significant correlations with instruments measuring perceived health, quality of life, and depression, anxiety and stress.   - ***Known-group validity***   ***Correlations:***  - There were no significant differences seen in any of the domains across gender, marital status, education level, ethnic groups, area of residence and employment status. However, results showed that those who had a chronic illness had significant lower scores on the timeline chronic, emotional representations, consequences and control negative, consequences positive and control-positive domains. |
| Brief-APQ | Moghadam et al., 2016 | Cross-sectional | Iran | Community-dwelling OA  *N:* 400 (60-75 = 291, 75-90 = 98, 90+ = 11)  *Age:* 60-90+  *Gender:* 50.5% F | *-* | **INTERNAL CONSISTENCY:**  - Cronbach’s was 0.75 for the whole scale and as 0.69, 0.53, 0.77, 0.67, and 0.70 for the subscales of timeline chronic, positive consequences, positive control, negative consequences and control, and emotional representations.  Correlation coefficient for the subscales: timeline chronic, positive consequences, positive control, negative consequences and control, and emotional representations was found to be 0.99, 0.90, 0.98, 0.99, and 0.95 respectively.  **RELIABILITY:**   - ***Test-retest stability***   - The test–retest correlation coefficient was found to be 0.95 (p < .001). | **CONTENT VALIDITY:**   - ***Content validity index***   - Based on the views expressed by the eight experts, CVI was found to be 0.97, 0.95, and 0.96 for relevance, clarity, and simplicity of items of the scale, respectively. CVI for the five subscales: timeline chronic, consequences positive, control positive, consequences and control negative, and emotional representations were found to be 1.00, 0.87, 0.95, 0.97, 0.95, respectively.  **STRUCTURAL VALIDITY:**   - ***Confirmatory Factor Analysis***   - CFA indicated appropriateness of the 17 items and five dimensions of the Persian version model of B-APQ. The fit indices were as follows: χ^2^ = 261.34, p < .001, RMSEA = 0.06, CFI 0 0.91, GFI = 0.93, AGFI = 0.90, RMR = 0.75. |
| Brief-APQ | Slotman et al., 2017 | Cross-sectional | Netherlands ( Turkish elderly migrants) | Community-dwelling OA  *N:* 438  *Age:* M = 72.82, SD = 5.24 (65–95)  *Gender:* 45.26% F | - | **INTERNAL CONSISTENCY:**  - The majority of APQ-S subscales had adequate internal consistency, with Cronbach’s α ranging from 0.75 (timeline chronic) to 0.88, except for timeline cyclical subscale (α = 0.56). | **STRUCTURAL VALIDITY:**   - ***Confirmatory Factor Analysis***   - The latent seven-factors model was found to have an acceptable fit, with RMSEA, SRMR, and CFI statistics within the boundaries of good fit (RMSEA = 0.06; SRMR = 0.07; CFI = 0.90).  **CRITERION VALIDITY:**   - ***Inter-factor and inter-instruments correlations***   - Several significant correlations were found between the subscales, and none exceed the critical point of 0.80. Comparison of the inter-factor correlations with correlations found in the Dutch version revealed no difference in directionality.  **CONSTRUCT VALIDITY:**   - ***Convergent and divergent validity***   ***Correlations:***  - The dimensions were correlated with SPF-IL, PCS and MCS of HRQoL, and the number of  chronic conditions. |
| Brief-APQ | Abe & Tomiyama, 2024 | Cross-sectional | Japan | Community-dwelling OA  *N:* 1500  *Age:* M= 72.97, SD = 4.94  *Gender:* 849 F |  | **INTERNAL CONSISTENCY:** - The factors showed good indexes: timeline-chronic, Cronbach’s α = 0.80; consequences positive, Cronbach’s α = 0.76; consequences and control negative, α = 0.91; control positive, Cronbach’s α = 0.85, and Cronbach’s α = 0.73 for emotional representations. | **STRUCTURAL VALIDITY:**   - ***Confirmatory Factor Analysis***   - The model’s goodness of fit included  χ2 (109) = 1319.735 (p < 0.001), GFI = 0.901, AGFI = 0.862, CFI = 0.906, and RMSEA = 0.086  **CONSTRUCT VALIDITY:**  - Correlations with cognitive and physical  functioning for timeline-chronic (r = 0.176, p < 0.001), consequences and control positive (r = −0.181, p < 0.001), and emotional representation (r = 0.171, p < 0.001) factors. Depression was associated with emotional representations (r = 0.379, p < 0.001); well-being was associated with positive consequences (r = 0.264, p < 0.001), control positive (r = 0.258, p < 0.001), and emotional representations (r = −0.260, p < 0.001). |
| **AGING-RELATED COGNITIONS SCALES (AGECOG)** | *Steverink et al., 2001 | Cross-sectional | Germany | Adults and community-dwelling OA  *N*: 4,034 *Age*: M = 60.1, SD = 12.18 (40-85) *Gender:* 48.8% | *N° of items:* 12  *Dimension(s):* 3 dimensions (physical decline, continuous growth, social loss)  *Response scale:* 4-point Likert scale (from “completely true” to “completely not true”) | **INTERNAL CONSISTENCY:**  - The internal consistency coefficients (Cronbach’s α) for the three factors were found to be 0.79, 0.78, and 0.77, which can be considered satisfactory. | **STRUCTURAL VALIDITY:**   - ***Exploratory Factor Analysis***   – EFA revealed a three-factor structure that accounted for 60.5% of variance.  **CONSTRUCT VALIDITY:**   - ***Convergent and divergent validity***   ***Regressions:***  - All three dimensions of the aging experience were also found to be related to both positive and negative effects and, with the exception of physical decline, to life satisfaction.   - ***Known-group validity***   ***Regressions:***  - Not only being younger but also having better subjective health, higher income, less loneliness, higher education, and greater hope were negatively associated with physical decline and social loss and positively associated with continuous growth. |
| AgeCog | Wurm et al., 2007 | Longitudinal | Germany | Adults and community-dwelling OA  *N:* 1,286  *Age:* M = 57, SD = 10.81 (40-85)  *Gender:* 47.5% F | *N° of items*: 8 items  *Dimensions*: 2 dimensions (AgeCog Physical Losses, AgeCog Ongoing Development)  *Response scale:* 4-point Likert scale (from “completely true” to “completely not true”) | **INTERNAL CONSISTENCY:**  - To compute the scale reliability of the three latent constructs with multiple items, the method proposed by Raykov (2004) was applied. The values suggest that the reliability of the three constructs is good at baseline and follow-up. | **STRUCTURAL VALIDITY:**   - ***Structural Equation Modeling***   **-** A model including AgeCog Physical Losses, AgeCog Ongoing Development and another Hope scale was conducted. This model showed a very good fit: χ^2^ = 366.33, df = 122 RMSEA =0.04, NNFI = 0.97, and SRMR = 0.023  **MEASUREMENT INVARIANCE:**  - Metric invariance was found for AgeCog Ongoing Development and partial invariance for AgeCog Physical Losses and Hope scales. Longitudinal invariance tests confirmed that factor loadings and error variances were invariant over time, ensuring consistent attributes and measurement precision across occasions. |
| **ATTITUDES TOWARD OWN AGING (ATOA), SUBSCALE FROM THE PHILADELPHIA GERIATRIC CENTER MORALE SCALE (PGCMS)** | *Lawton, 1975 | Cross-sectional | United States | Adults and community-dwelling OA  *N:* 828  *Age:* M = 72.6  *Gender:* 72% F | *N° of items:* 5 items for the subscale named “Attitudes towards own aging”  *Dimension(s):* unidimensional  *Response scale:* 4-point Likert scale (from “strongly disagree” to “strongly agree”) | **INTERNAL CONSISTENCY:**  - The ATOA scale demonstrated good internal consistency (α = 0.81). | **STRUCTURAL VALIDITY:**   - ***Exploratory Factor Analysis***   - Two rotations, a six-factor and a five-factor Varimax rotation, were performed, explaining 50% and 46% of the total variance, respectively.  - A three-factor solution with a rotated Varimax rotation was derived from 15 items, accounting for 43% of the total variance for the general PGCMS scale, of which the ATOA represents one dimension. |
| ATOA | Niklasson et al., 2015 | Cross-sectional | Sweden | Community-dwelling OA  *N*: Sample 1 = 493*;* Sample 2 = 54  *Age*: Sample 1: M = 89, SD = 4.4*;* Sample 2: M = 84.7, SD = 6.7  *Gender*: Sample 1 = 64.5% F; Sample 2 = 66.7% F | *-* | **INTERNAL CONSISTENCY:**  -The ATOA scale demonstrated low internal consistency (α = 0.48).  **RELIABILITY:**   - ***Test-retest stability***   - Intra-rater test–retest reliability analysis for the 17 items of the PGCMS showed that absolute agreement varied between 63.0 and 90.7 % and Cohen’s Kappa varied between 0.24 and 0.77.  - The results from the intra-rater test–retest within 1 week from the sample 2 show an intraclass correlation coefficient of 0.89 of the PGCMS. | **STRUCTURAL VALIDITY:**   - ***Confirmatory Factor Analysis***   - The 17-item three-factor model of PGCMS scale showed a good fit for all indices except CFI: chi = 217, df = 114, p < .001, RMSEA = 0.04, PCLOSE = 0.91, GFI = 0.95, AGFI = 0.93, CFI = 0.90. |
| ATOA | Wong et al., 2004 | Cross-sectional | China | Community-dwelling OA and older residents  *N*: community-dwelling OA = 759; older residents = 388  *Age*: Old = 65–74; Old-old = 75–89; Very old = 90  *Gender*: community-dwelling OA = 56.4% F; older residents = 70.4% F | *-* | NA | **STRUCTURAL VALIDITY:**   - ***Exploratory Factor Analysis***   - The EFA using principal axis factoring and Promax rotation revealed two underlying factors for the PGCMS scale, called “reconciled aging” and “unstrained affect”.   - ***Confirmatory Factor Analysis***   - The CFA also proved that this two-factor model adequately fit all the samples. Fit indices were as follows: CFI = 0.96, GFI = 0.99, RMSEA = 0.04, NFI = 0.95, NNFI = 0.96, RFI = 0.93.   - ***Item Response Theory***   - According to the Rasch model, the items were easily endorsed with positive responses by community-dwelling OA and by older residents for the unstrained effect scale.  **CONSTRUCT VALIDITY:**   - ***Divergent validity***   ***Correlations:***  Construct validity is demonstrated by the good overall correlation between the reconciled aging and unstrained affect domains and the GDS total scores. |
| **50 item - AWARENESS OF AGE-RELATED CHANGE QUESTIONNAIRE (50-AARC)** | *Brothers et al. 2019 | Cross-sectional | United  States & Germany | Community-dwelling OA  *N:* Study 1 = 396; Study 2 = 424  *Age:* Study 1: M = 65.45, SD = 13.75 (40-98); Study 2: M = 69.53, SD = 12.52 (42-98)  *Gender:* Study 1 = 55.3% F; Study 2 = 52.4% F | *N° of items:* 50  *Dimension(s):* 2 dimensions (AARC-Gains: health and physical functioning, cognitive functioning, interpersonal relations, social-cognitive and social-emotional functioning, lifestyle and engagement; AARC-Losses: health and physical functioning, cognitive functioning, interpersonal relations, social-cognitive and social-emotional functioning, lifestyle and engagement)  *Response scale:* 5-point Likert scale (from “not at all” to “very much”) | - **189-item version**   **INTERNAL CONSISTENCY:**  - Internal consistency reliability and item-total correlations (ITC) were calculated for both AARC-Gains and AARC-Losses, as well as the 10 theorized behavioral domains. Coefficients ranged from α = 0.79 for the Interpersonal Relations–Negative scale to α = 0.92 for the Cognitive Functioning–Negative scale.   - **50-item version**   **INTERNAL CONSISTENCY:**  - Cronbach's α coefficients ranged from 0.73 to 0.89, indicating good reliability of the reduced subscales. Item-level reliabilities were also good overall, with most items exceeding the cutoff of 0.30.  - Correlations between the subscales of the long and short versions of the questionnaire were high, ranging from 0.82 to 0.98. | - **189-item version**   **STRUCTURAL VALIDITY:**   - ***Exploratory Factor Analysis***   - EFA with Promax rotation was conducted, explaining 68.23% of the total variance. Two factors emerged, with Factor 1 accounting for 47.54% and Factor 2 accounting for an additional 20.69% of the variance.   - ***Confirmatory Factor Analysis***:   - CFA showed a satisfactory overall fit of the two-factor model: χ^2^ = 72.92, df = 29, p < .01, CFI = 0.97, TLI = 0.95, RMSEA = 0.09, SRMS = 0.06.   - **50-item version**   **CONTENT VALIDITY:**   - ***Item reduction***   - An item was marked for deletion if doing so would not decrease the scale reliability below an acceptable level (α = 0.70), if it was redundant, and if it had low ITC.  **STRUCTURAL VALIDITY:**   - ***Confirmatory Factor Analysis***   - CFA confirmed two-factor structure with acceptable fit: χ^2^ = 51.11, df = 30, p < .01, CFI = 0.99, TLI = 0.99, RMSEA = 0.04, SRMS = 0.03.  **CONSTRUCT VALIDITY:**   - ***Convergent and divergent validity***   ***Correlations:***  - AARC-Losses showed significant correlations with other measures of subjective aging, such as felt age, ATOA, and the AgeCog scales, indicating convergent validity.  - A more negative view of one’s own aging was associated with higher scores on AARC-Losses. AARC-Gains, on the other hand, showed a significant association only with the ongoing development subscale of the AgeCog scales, indicating divergent validity. |
| **10-ITEM AWARENESS OF AGE-RELATED CHANGE QUESTIONNAIRE (10-AARC)** | Kaspar et al., 2019 | Cross-sectional | United States & Germany | Community-dwelling OA  *N:* 819  *Age:* M = 64.13, SD = 12.85 (40-98)  *Gender:* 60% F | *N° of items:* 10  *Dimension(s):* 2 dimensions (AARC-Gains: health and physical functioning, cognitive functioning, interpersonal relations, social-cognitive and social-emotional functioning, lifestyle and engagement; AARC-Losses: health and physical functioning, cognitive functioning, interpersonal relations, social-cognitive and social-emotional functioning, lifestyle and engagement)  *Response scale:* 5-point Likert scale (from “not at all” to “very much”) | **INTERNAL CONSISTENCY:**  - The relative model fit and estimated reliability for the gains and losses composites were deemed acceptable, indicating satisfactory reliability of the measures. McDonald’s omega in the total sample were 0.72 for AARC-Gains and 0.80 for AARC-Losses. Considering only the old age subsample (70+) omega were 0.71 for Gains and 0.79 for Losses. | **STRUCTURAL VALIDITY:**   - ***Item Response Theory*:**   - Item parameter estimates from the comprehensive Generalized Partial Credit Model of Gains and Losses were used in the item selection process for the 10-AARC. To retain the theoretical structure of the concept of AARC, two items per behavioral domain with significant loadings were selected, one reflecting a gain-related experience and the other a loss-related experience. The item subset selected for the 10-AARC covered the perceived age-related gains and losses across a reasonable range of the latent target domains with satisfying precision.  **MEASUREMENT INVARIANCE:**  ***Structural equation modeling:***  - Compared to a baseline model with factor loadings and item intercepts freely estimated in each age group, restricting factor loadings to be the same across all three age groups did not increase model misfit substantially (M1: Δχ^2^ = 19.55, Δdf = 20, p =.49). Hence, the meaning of the concept of AAARC as captured byu the 10-AARC appeared to be the same across the age span considered, allowing for valid representation of AARC-Gains and Losses in correlational studies covering a wide range of the adult life span.  - However, there was evidence of a response shift, suggesting that individuals may have different perceptions of age-related gains and losses as they age.  **CONSTRUCT VALIDITY**   - ***Criterion validity:***   ***Correlations*:**  - High correlations were observed between the AARC-50 and AARC-10, with values as high as 0.89 for perceived age-related losses and 0.88 for perceived age-related gains. This indicates strong convergence between the two versions of the AARC questionnaire.  **CONSTRUCT VALIDITY:**   - ***Convergent and divergent validity***   ***Correlations:***  - The overall pattern of associations between AARC and external criterion variables related to subjective aging (ATOA), psychological wellbeing (SWLS, SPWB), and health (SF-36, CES-D-R10) was very similar for both the original 50-item and the proposed 10-item version. |
| 10-AARC | Neri et al., 2021 | Cross-sectional | Brazil | Community-dwelling OA  *N*: 387  *Age*: M = 67.9, SD = 5.6  *Gender*: 59.7% F | *-* | **INTERNAL CONSISTENCY:**  - The scale and its factors were tested for internal consistency using Cronbach’s α. The levels of internal consistency were high (from α = 0.70 to α = 0.85), further supporting the evidence of internal validity. | **STRUCTURAL VALIDITY:**   - ***Exploratory Factor Analysis***   - EFA revealed 2 factors explaining 55.4% variance. Then, Varimax and Oblimin rotations were applied.   - ***Confirmatory Factor Analysis***   - CFA confirmed the two-factors structure. Two sequences of item reallocation were performed, resulting in a return to the first factorial solution. The fit indices were as follows: chi = 27.21, df = 26, GFI = .095, AGFI = 0.91, CFI = 0.99, NNFI = 0.99, SRMR = 0.06, RMSEA = 0.02.  **MEASUREMENT INVARIANCE:**  **-** The sample was divided twice. First, randomly, and second by age to test the hypothesis of invariance of the composition of the factors for age group. For both EFA and CFA, the results for the 60-69 years subsample were similar to those of the randomized subsamples. This factorial solution was not maintained on the CFA to the data from the second 70-80+ years subsample.  **CONSTRUCT VALIDITY:**   - ***Convergent validity***   ***Correlations:***  - For the total sample and age subsamples, negative correlations between frailty score and 10-AARC scale in gains were found. The inverse pattern was identified for losses: the higher the frailty score, the higher the losses score. This pattern repeated for self-rated health compared with peers of the same age, with systematically lower correlations, except for losses among individuals aged 70 and over. |
| 10-AARC | Sabatini et al., 2020 | Longitudinal | UK | Community-dwelling OA  *N*: 9,410  *Age*: M = 65.9, SD = 7.1 (51-95)  *Gender*: 79.9% F | *-* | **INTERNAL CONSISTENCY:**  - For the AARC-10 SF item-to-total score correlations had values between 0.67 and 0.78. Cronbach’s α was 0.77 for the Gains scale and 0.80 for the Losses scale. | **STRUCTURAL VALIDITY:**   - ***Confirmatory Factor Analysis***   **-** CFA confirmed the hypothesized two-factor model. The fir indices were as follows: RMSEA = 0.07, CFI = 0.94, TLI = 0.92, SRMR = 0.05**.**  **MEASUREMENT INVARIANCE:**  **-** Compared to the model with all parameters freely estimated, the model that restricted factor loadings to be the same across gender and educational levels did not reduce model fit substantially. Hence, the meaning of the concepts of AARC-Gains and Losses as captured but the 10-AARC appeared to be the same for males and females.  **CONSTRUCT VALIDITY:**   - ***Known-group validity***   ***Regressions:***  - Being older, employed, and having a university education predict lower levels of AARC-Gains, while being female predicts higher levels of AARC-Gains. Furthermore, being female, married, in a civil partnership, or co-habiting, and having a university education predict fewer AARC-Losses; while being older predicts more AARC-Losses. |
| 10-AARC | Testad et al., 2022 | Cross-sectional | Norway | Community-dwelling OA  *N*: 1,510  *Age*: Median = 63.2, IQR = 57.1-69.1  *Gender*: 79.7% F | *-* | **INTERNAL CONSISTENCY:**  - Cronbach’s α demonstrated acceptable internal consistency of 0.74 for AARC-Gains and 0.81 for AARC-Losses. | **STRUCTURAL VALIDITY:**   - ***Confirmatory Factor Analysis***   ***-*** CFA with additional cross-loading confirmed the hypothesized two-factors model. The fir indices were as follows: RMSEA = 0.05, CFI = 0.98, TLI = 0.96, SRMR = 0.03.  **MEASUREMENT INVARIANCE:**   - **Multigroup Confirmatory Factor Analysis**   - Measurement invariances hold for the classes defined by sex, marital status, and education group, since all goodness-of-fit measures fell within the pre-defined range for acceptable model fit. For the model with additional cross-loading, the invariance was also established for age group and current employment.  **CONSTRUCT VALIDITY:**   - ***Convergent and divergent validity***   ***Correlations*:**  - Gains and Losses were differentially correlated with cognitive functioning. Higher scores on AARC-Losses showed either negligible or small associations with poorer performance in almost all the cognitive tasks examined except from scores on the Stroop test. On the other hand, higher AARC-Gains were not significantly correlated with most of the cognitive tasks, except for poorer performance on verbal reasoning.   - ***Known-group validity***   ***Regressions:***  - Higher scores of AARC-Gains were associated with younger age and being female; lower scores of AARC-Losses were associated with university education, being currently employed, and being married, or in a civil partnership, or co-habiting. |
| 10-AARC | Schönstein et al., 2023 | Cross-sectional | Germany & Burkina Faso | Adults and community-dwelling OA  Burkina Faso sample:  *N:* 3,028  *Age: M= 54.31, SD = 11*  *Gender:* 50% F  German sample:  *N:* 541  *Age:* M= 68.85, SD = 8.2  *Gender:* 57% F | *-* | NA | **STRUCTURAL VALIDITY:**   - ***Confirmatory Factor Analysis***   - The two-factor structure (AARC-Gains and AARC-Losses) fits for both Burkina Faso (CFI=0.912, SRMSR= 0.046, RMSEA [90% CI]= 0.072 [.067; .077]) and German (CFI=0.887, SRMSR= 0.081, RMSEA [90% CI]= 0.080 [.067; .094]) samples.  **MEASUREMENT INVARIANCE**:  - A simultaneous factor analysis assessed the invariance of the factor structure across age groups (below and above 60 years of age). The similar fit indices (CFI, SRMSR, RMSEA) across the different analyses (configural, weak, and strong) indicate that the AARC construct is measured consistently across age groups. |
| 10-AARC & 50-AARC | Genç et al., 2023 | Cross-sectional | Turkey | Adults and community-dwelling OA  *N:* 570  *Age:* M= 53.4, SD=10.4  *Gender:* NA |  | **INTERNAL CONSISTENCY:**  - 50-AARC: Cronbach’s α for the AARC-50 Gains and AARC-50 Losses dimensions were 0.943 and 0.887, respectively.  - 10-AARC: Cronbach’s α for the AARC-10 Gains and AARC-10 Losses dimensions were 0.806 and 0.642, respectively.  **RELIABILITY:**   - ***Test-retest stability***   - Correlation coefficient between the test-retest scores (after 14 days) was found to be 0.90 (p < 0.001; n = 570). | **CONTENT VALIDITY**  - Based on the opinions from 9 experts, CVI was found to be 0.90, with an agreement of 90%.  **CONSTRUCT VALIDITY**   - ***Confirmatory factor analysis***   - 50-AARC: Two-factor structure was confirmed (χ2/df= 3.077, CFI= 0.785, GFI=0.770, RMSA= 0.060, SRMR= 0.081)  - 10-AARC: Two-factor structure was confirmed (CMIN/DF=3.661; RMSA= 0.068; CFI= 0.931; GFI= 0.958; NFI= 0.909; AGFI = 0.932).   - **Criterion‑related/concurrent validity**   - 50-AARC: Correlations between SF-12 physical component dimension and the AARC-50 gains sub-dimension (r = 0.035, p = 0.409), AARC-50 losses subdimension (r = − 0.271, p < 0.001). Correlations SF-12 mental component and the AARC-50 gains sub-dimension (r = 0.113, p = 0.007), AARC-50 losses sub-dimension (r = − 0.528, p < 0.001).  - 10-AARC: Correlations between SF-12 physical component dimension and AARC-10 gains subdimension (r = 0.042, p = 0.322), and AARC-10 losses subdimension (r = − 0.283, p < 0.001). Correlations SF-12 mental component and AARC-10 gains sub-dimension (r = 0.051, p = 0.225), and AARC-10 losses sub-dimension (r = − 0.453, p < 0.001). |
| 10-AARC | Tseng et al., 2024 | Cross-sectional | Taiwan & Germany | Adults and community-dwelling OA  Study 1  Taiwan sample =  *N:* 292  *Age:* M=77.38, SD=6.24  *Gender:* 52%  Study 2  German sample =  *N:* 118  *Age:* M=71.18, SD=4.14  *Gender:* 69%  Taiwan sample =  *N:* 297  *Age:* M=74.26 SD=4.04  *Gender:* 56% |  | Study 1  **INTERNAL CONSISTENCY:**  - Adequate internal consistency for the two dimensions: Cronbach’s α = 0.79 for AARC-Gains and Cronbach’s α = 0.82 for AARC-Losses | Study 1  **CONSTRUCT VALIDITY**   - ***Confirmatory factor analysis***   - Two-factor structure was confirmed [χ2(34) = 78.80, *p* < .001, CFI = 0.96, TLI = 0.95, RMSEA = 0.06, SRMR = 0.03].   - ***Convergent* validity**   ***Correlations:***  - Correlations (\|*r\|*) with other subjective VOA (Felt Age, ATOA, EBA) ranged from 0.21to 0.52, for AARC-Losses, whereas the correlation coefficients for AARC-Gains ranged from 0.12 to 0.27.   - ***Discriminant* validity**   ***Correlations:***  - EBA scores were positively associated with AARC-Gains (r=0.12), and negatively associated with AARC-Losses (r=-0.33)   - ***Criterion*** validity   ***Correlations:***  - AARC-Gains correlated with SF-12 physical component (*r* = 0.17), but not significantly associated with SF-12 mental component or chronic disease burden.  - AARC-Losses correlated with chronic disease burden (r = 0.30), lower levels of SF-12 physical component (r = −0.43), and lower levels of SF-12 mental component (r = −0.38).  Study 2  MEASUREMENT INVARIANCE   - **Gender**   - Compared to the configural invariance model, the metric invariance model between men and women did not decrease the model fit: men and women responded to the items of the AARC-Gains and AARC-Losses subscales in a similar way.   - **Age groups**   - The scalar invariance model yielded a significantly worse fit compared to the metric fit: individuals may experience age-related changes that are intrinsically and qualitatively different at different stages of later adulthood.   - **Countries**   - The scalar invariance model yielded a significantly worse fit compared to the metric fit: differences among Taiwanese and German samples in AARC dimensions. |
| **FELT AGE: SINGLE ITEM ADAPTED** | *Barrett, 2003 | Cross-sectional | United States | Adults and community-dwelling OA  *N:* 2,864  *Age:* M = 44.97, SD = 13.37 (25-74)  *Gender:* 55.9% F | *N° of items:* 1  *Dimension(s):* unidimensional (years)  *Response scale:* numerical rating scale | NA | NA |
| **MEMORY CONTROLLABILITY INVENTORY (MCI) & AGING CONCERNS SCALE (ACS)** | *Lachman et al., 1995 | Cross-sectional | United States | Adults and community-dwelling OA  *N:* Sample 1 = 140; Sample 2 = 209; Sample 3 = 162  *Age:* Sample 1: M = 69.31, SD = 5.42 (60-85); Sample 2: 71.58, SD = 6.33 (55-86); Sample 3: 54.14, SD = 22.07 (20-90)  *Gender:* Sample 1 = 65.7% F; Sample 2 = 32.5% F; Sample 3 = 59.3% F | *N° of items:* 12 (MCI) and 7 (ACS)  *Dimension(s):* For MCI, 4 dimensions (present ability, potential improvement, effort utility, inevitable decrement); For ACS, 2 dimensions (independence, Alzheimer’s likelihood)  *Response scale:* 7-point Likert scale (from “strongly disagree” to “strongly agree”) | **MCI**  **INTERNAL CONSISTENCY:**  - The coefficient alphas across the three studies ranged from 0.58 to 0.70 for the Present Ability scale, 0.62 to 0.75 for the Potential Improvement scale, 0.65 to 0.73 for the Effort Utility scale, and 0.58 to 0.77 for the Inevitable Decrement scale.  - The item-to-total correlations ranged from 0.30 to 0.68.  **RELIABILITY**:   - ***Test-retest stability***   - The test-retest reliability of the four scales ranged from 0.50 to 0.65 for the 9-day posttest and from 0.46 to 0.57 for the three-month posttest.  **ACS**  **INTERNAL CONSISTENCY:**  - The coefficient alphas across the three studies ranged from 0.49 to 0.68 for the Independence scale and from 0.65 to 0.73 for the Alzheimer’s scale.  **RELIABILITY**:   - ***Test-retest stability***   - After 9-day interval: r ranged from 0.52 to 0.67  - After three-month interval: r ranged  from 0.59 to 0.65. | **MCI**  **STRUCTURAL VALIDITY*:***   - ***Confirmatory Factor Analysis***   - CFA was employed to assess the 3-factors structure. The priori model provided a good fit in all three samples. Sample 1: χ^2^ = 80.26, df = 45, p < .001, GFI = 0.92, AGFI = 0.85; Sample 2: χ^2^ = 58.26, df = 45, p =.09 , GFI = 0.95, AGFI = 0.92; Sample 3: χ^2^ = 69.90, df = 45, p = .01 , GFI = 0.93, AGFI = 0.89.  **CONSTRUCT VALIDITY:**   - ***Convergent validity***   ***Correlations*:**  **-** Belief in inevitable memory loss was linked to poorer memory performance, while higher perceptions of memory ability, optimism, and effort to improve memory were associated with better performance.  - The highest correlation was found between the PIC Chance scale and MCI Inevitable Decrement, both reflecting the belief that cognitive aging is inevitable. The lowest correlations were between PIC Powerful Others and MCI scales, as the latter does not measure beliefs about control by powerful others.   - ***Known-group validity***   ***Correlations*:**  - In Sample 3, age was significantly correlated with the MCI Ability (r = -0.24, p < .03) and Decrement (r = 0.30, p < .007) subscales, but only in the older age group (65-90). Older adults were more likely to believe in inevitable memory decline and have less confidence in their memory. No consistent differences were found by sex or education.  **ACS**  **STRUCTURAL VALIDITY*:***   - ***Confirmatory Factor Analysis***   - CFA was employed to assess the 2-factors structure. The a priori model provided a good fit in all three samples. Sample 1: χ^2^ = 18.26, df = 14, p = .19, GFI = 0.96, AGFI = 0.93; Sample 2: χ^2^ = 10.46, df = 12, p =.58, GFI = 0.99, AGFI = 0.97; Sample 3: χ^2^ = 20.80, df = 12, p = .05, GFI = 0.97, AGFI = 0.92.  **CONSTRUCT VALIDITY:**   - ***Convergent validity***   ***Correlations*:**  - Independence in managing memory (without relying on others) correlated with higher scores on Present Ability, Potential Improvement, and Effort Utility, and rejection of inevitable memory loss. Conversely, concerns about Alzheimer’s disease were linked to lower perceived memory ability and a greater belief in inevitable memory decline. |
| **MEMORY FUNCTIONING QUESTIONNAIRE (MFQ)** | *Gilewski et al. 1988 | Longitudinal | United States | Adults and community-dwelling OA  *N:* 778  *Age:* M = 56.9, SD = 20.8 (16-89)  *Gender:* 55.9% F | *N° of items:* 64  *Dimension(s):* 4 dimensions (general frequency of forgetting, seriousness of forgetting, retrospective functioning, mnemonics usage)  *Response scale:* 7-point Likert scale (ranging from “Always” to 7 “Never” for frequency-related items, and “very serious/bad” to “not serious/very good” for severity-related items) | **INTERNAL CONSISTENCY:**  - The internal consistency estimates (Cronbach’s α) for the four factor scores were 0.94, 0.94, 0.89, and 0.83, indicating that the factors are highly reliable.  **RELIABILITY:**   - ***Test-retest stability***   - The stability of the MFQ was assessed by comparing the covariance matrices of the scales comprising the factor scores longitudinally.  - The covariance matrices did not show significant differences (χ^2^ = 35.48, df = 26, p = .49), indicating stability, and the goodness of fit of the comparison was 0.99. | **STRUCTURAL VALIDITY:**   - ***Exploratory Factor Analysis***   - EFA using principal-axis factoring was employed, revealing three four-factor domains, explaining 36.7% of variance.  - PCA with an oblique (Oblimin) rotation was performed, and factor loadings of at least 0.35 were considered significant.  **MEASUREMENT INVARIANCE**:  - The covariance matrices for the summed responses to the MFQ scales for the two age groups (16-54 vs 55-89) were analyzed. There were no reliable age differences: χ^2^ = 34.16, df = 36, p = .55. Invariance of the factor structure was confirmed across age groups, two independent samples, and over a span of three years. |
| MFQ | Zelinski et al., 1990 | Cross-sectional | United States | Community-dwelling OA  *N:* Sample 1 = 198; Sample 2 = 89  *Age:* Sample 1: M = 67.85, SD = 6.89; Sample 2: M = 70.59, SD = 6.05  *Gender:* Sample 1 = 51,5% F; Sample 2 = 59.6% F | *-* | **INTERNAL CONSISTENCY:**  **-** The internal consistency of MFQ factor scores was also high, with Cronbach’s α ranging from 0.94 to 0.84. | **STRUCTURAL VALIDITY:**  - The covariance matrix for scores on the MFQ scales from was compared with that of an independent sample of subjects from the MFQ factor analysis study (Gilewski et al, 1990). There were no differences between matrices, χ^2^ = 12.72, df = 36, p = 1, GFI = 0.98, indicating that the relationships between MFQ scales was invariant across samples of older adults  **CONSTRUCT VALIDITY**   - ***Concurrent validity***   ***Regressions***:  - Results revealed moderate concurrent validity with memory measures (self-appraisal of everyday memory functioning and several memory tasks). |
| MFQ | Pedone et al., 2005 | Cross-sectional | Italy | Adults and community-dwelling OA  *N:* 506  *Age:* M = 44.21, SD = 14.36 (20-70)  *Gender*: 55.1% F | *-* | **INTERNAL CONSISTENCY:**  - Cronbach’s α was satisfactory for the 4 subscales (ranging from 0.70 to 0.95). | **STRUCTURAL VALIDITY:**   - ***Confirmatory Factor Analysis***   **-** CFA confirmed a 4-factor structure: χ^2^ = 174.9, df = 48, p < .001, RMSEA = 0.07, NNFI = 0.97, CFI = .98.  **MEASUREMENT INVARIANCE**:  - Simultaneous CFA indicated that the factor structure was invariance across sex (χ^2^ diff = 8.58, df = 8, p = .37), age (χ^2^ diff = 21.37, df = 16, p = .16), and education (χ^2^ diff = 23.44, df = 16, p = .10). |
| MFQ | Revell et al., 2001 | Cross-sectional | United States | Community-dwelling OA  *N*: 367  *Age*: M = 71.83, SD = 8.07  *Gender*: 56.9% F | *-* | NA | **STRUCTURAL VALIDITY:**   - ***Exploratory Factor Analysis***   - PCA with Promax rotation of the three and six factor solutions accounted for the highest total variances (41-49% and 62-75%, respectively). The six-factor solution was not parsimonious and was thus dropped in favor of the three.  **MEASUREMENT INVARIANCE**:  - Strict factorial invariance was present between the groups of age (χ^2^ diff = 1.86, df = 6, NFI = 0.86, TLI = 0.93, CFI = 0.94, RMSEA = 0.05) and education (χ^2^ diff = 2.17, df = 6, NFI = 0.87, TLI = 0.91, CFI = 0.92, RMSEA = 0.06) and weak factorial invariance was present for male and female groups (χ^2^ diff = 2.04, df = 4, NFI = 0.90, TLI = 0.92, CFI = 0.94, RMSEA = 0.05). |
| **METAMEMORY IN ADULTHOOD (MIA)** | Dixon & Hultsch, 1983a | Cross-sectional | United States | Young adults and community-dwelling OA  *N:* Sample 1: YA = 60; OA = 60; Sample 2: YA = 36; middle-aged= 36; OA = 36;  Sample 3: YA = 50; middle-aged = 50; OA = 50  *Age:* Sample 1: YA = 18-37; OA = 50-81; Sample 2: YA = 21-39; middle-aged = 39-58; OA = 60-84; Sample 3: YA = 21-39; middle-aged = 39-58; OA = 60-74  *Gender:* 90% *F* | *N° of items:* 108  *Dimension(s):* 8 dimensions (knowledge of memory strategies [strategy], knowledge of memory tasks and processes [task], knowledge of own memory capacities [capacity], attitudes toward own memory perception of change [change], activities supportive of memory [activity], memory and state anxiety [anxiety], memory and achievement motivation [achievement], locus of control in memory abilities [locus])  *Response scale:* 5-point Likert scale (from “agree strongly” to “disagree strongly”) | **INTERNAL CONSISTENCY:**  - The estimate of internal consistency for the overall instrument was found to be acceptable for all age groups in each sample. Cronbach’s α for the total scale in each sample was also within an acceptable range (Sample 1 = 0.85, Sample 2 = 0.87, Sample 3 = 0.89). | **STRUCTURAL VALIDITY:**   - ***Exploratory Factor Analysis***   - An EFA with an oblique rotation (Promax) was conducted on the MIA in each of the three samples with the goal of obtaining an eight-factor solution based on both theoretical and empirical considerations.  **CONSTRUCT VALIDITY:**   - ***Know-group validity***   ***MANOVA:***  - Younger adults scored significantly higher on the Task, Capacity, and Change scales and there was no difference for the Strategy scale. This outcome was confirmed on the three separate samples. |
| MIA | Dixon & Hultsch, 1983b | Cross-sectional | United States | Adults and community-dwelling OA  *N:* Sample 1: YA = 30, OA = 30;  Sample 2: YA = 36, 36 middle-aged, OA = 36; Sample 3: YA = 50 YA, middle-aged = 50, OA = 50  *Age:* Sample 1: YA = 18-32; OA = 60-81; Sample 2: YA = 21-39;  middle-aged = 39-58; OA = 60-84; Sample 3: YA = 21-39; middle-aged = 39-58; OA = 60-74).  *Gender:* NA | *-* | NA | **CONSTRUCT VALIDITY:**   - ***Convergent validity***   ***Regressions:***  -Strategy (followed by Capacity and Task) is the best predictor of memory performance by young adults; For middle-aged adults, Strategy (followed by Task, Capacity, and Achievement) are the best predictors; for older adults Task (followed by Achievement and Locus) are the most important predictors, and Strategy is relatively unrelated. |
| MIA | Hertzog et al., 1987 | Cross-sectional | United States | Adults and community-dwelling OA  *N:* Sample 1: YA = 60; OA = 60; Sample 2:YA = 36; middle-aged = 36; OA = 36; Sample 3: YA = 50; middle-aged = 50; OA = 50; Sample 4: YA = 60; middle-aged = 60; OA = 60; Sample 5: YA = 24; middle-aged = 24; OA = 24; Sample 6: YA = 60; OA = 60.  *Age:* Sample 1: YA = 22.9; OA = 66.9; Sample 2: YA = 32.3; middle-aged = 48.5; OA = 68.5; Sample 3: YA = 32.0; middle-aged = 49.5; OA = 68.9; Sample 4: YA = 23.10; middle-aged = 44.17; OA = 66.83; Sample 5: YA = 20.50; middle-aged = 44.71; OA = 68.55; Sample 6: YA = 28.71; OA = 67.20;  *Gender:* NA | *-* | NA | **STRUCTURAL VALIDITY:**   - ***Confirmatory Factor Analysis***   - A two-factors model was adopted for the MIA. After several modifications, the final model fitted the validation sample quite well (χ^2^ = 38.76, df = 37, GFI = 0.94)  **MEASUREMENT INVARIANCE**:  - A simultaneous factor analysis assessed the invariance of factor structure across the three age groups. There was a significant loss of fit the in model constraining the factor loadings to be equal over all age groups. This indicated that the hypothesis of invariance should be rejected. |
| MIA | Hertzog et al., 1989 | Cross-sectional | United States, Canada | Adults and community-dwelling OA  *N:* Sample 1 = 360 (YA = 96, OA = 264);  Sample 2 = 415 adults  *Age:* Sample 1: YA: M = 22.11, SD =1.85 (20-26); OA: M =65.88, SD =5.45 (55-78); Sample 2: M = 52.33, SD =13.67 (20-78).  *Gender*: NA | *-* | NA | **STRUCTURAL VALIDITY:**   - **Confirmatory Factor Analysis**   - The model fitted well in both samples. Sample 1: χ^2^ = 91.44, p = .003, GFI = 0.97. Sample 2: χ^2^ = 60.24, p = .36, GFI = 0.97).  **MEASUREMENT INVARIANCE**:  - A simultaneous factor analysis assessed the invariance of factor structure across the two age groups. There was no significant loss of fit the in model constraining the factor loadings to be equal over age groups. This confirmed the hypothesis of invariance.  **CONSTRUCT VALIDITY:**   - ***Convergent validity***   ***Correlations:***  - Correlations between the MIA and the MFQ questionnaires converge to measure a construct labeled “memory self-efficacy”. |
| MIA | McDonough et al., 2019 | Cross-sectional | United States | Community-dwelling OA  *N*: Sample 1 = 382; Sample 2 = 221  *Age*: Sample 1: M = 74.2, SD = 8.7; Sample 2: M = 80.3, SD = 9  *Gender*: Sample 1 = 72.5% F; Sample 2 = 79.1% F | *-* | **INTERNAL CONSISTENCY:**  - Cronbach’s α (0.89) resulted in strong internal reliability. | **STRUCTURAL VALIDITY:**   - ***Exploratory Factor Analysis***   - PCA revealed 4 factors with eigenvalues exceeding 1. Using the scree test, two components were retained. Then an Oblimin rotation was used.   - **Confirmatory Factor Analysis**   - CFA confirmed the two-factors structure. Fit indices were as follow: χ^2^ = 394.58, df = 167, p < .001, RMSEA = 0.07, SRMR = 0.07, CFI = 0.85.  **CONSTRUCT VALIDIY**:   - ***Convergent validity***   ***Regressions***:  ***- Hierarchical*** regression model showed that the revised MIA Change subscale can predict memory performance (RBMT). |
| MIA | Ponds & Jolles, 1996 | Cross-sectional | Netherlands | Adults and community-dwelling OA  *N:* 1,899  *Age:* M = 52, SD = 16.9  *Gender*: 54% F | *-* | **INTERNAL CONSISTENCY:**  - Cronbach’s α ranged from 0.73 to 0.91 and confirmed high internal consistency for all factors.  **RELIABILITY:**   - ***Test-retest stability***   - The stability coefficients were satisfactory for both the 108-item instrument (range = 0.79 to 0.86) and the abridged version (range = .072 to 0.85), except for the Task subscale | **STRUCTURAL VALIDITY:**   - ***Confirmatory Factor Analysis***   - A CFA revealed that a substantial number of items (34 items, or 31%) on the MIA could be eliminated without loss of its 7-factor structure.  **MEASUREMENT INVARIANCE**:  - The same CFA was performed for three age groups. The goodness-of-fit index was 0.96 for the young subsample, 0.99 for the middle-aged subsample, and 0.96 for the old subsample, whereas the overall adjusted goodness-of-fit index was 0.96. The likelihood ratio χ^2^ test was significant. The Bentler-Bonnett normed fit index was 0.93. These data suggest that the factor structure of the abridged MIA is invariant over age.  **CONSTRUCT VALIDITY:**   - ***Divergent validity***   - The divergent validity of the MIA was supported by the fact that no significant correlation was found between depression and anxiety (the MIA was controlled for age, sex, and education).   - **Known-group validity**   **-** Age differences were found on five of the seven subscales of the MIA: Capacity, Change, Anxiety, Achievement, and Strategy. There appear to be few and only small sex differences. |
| MIA | Van Ede, 1995 | Cross-sectional | South Africa | Adults and community-dwelling OA  *N*: 902  *Age*: 20–60+ years  *Gender*: NA | *-* | **INTERNAL CONSISTENCY:**  **-** Cronbach’s α coefficients demonstrated high internal consistency across factors (ranging from 0.68 to 0.94). | **STRUCTURAL VALIDITY:**   - ***Exploratory Factors Analysis***   - The factorial validity of the MIA was investigated by population, age and gender group, as well as for the total group.  - An eight-factor structure was obtained in comparison with the seven factors reported from American and Canadian samples. |
| **PERCEPTIONS OF AGE (AD HOC QUESTIONS: AGE IDENTITY, COMPARATIVE AGE, FELT AGE)** | *Benyamini & Burns, 2020 | Longitudinal | United States | Community-dwelling OA  *N:* 851  *Age:* M = 73, SD = 7  *Gender:* 60% F | *N° of items:* 4  *Dimension(s):* 4 dimensions (Age-group identity; Comparative age; Felt age; Subjective nearness-to-death)  *Response scale:* Age-group identity (ranging from “teenager” to “very old”); Comparative age (from “much younger” to “older”); Felt age (years); Subjective nearness-to-death (years) | NA | NA |
| **POSITIVE AGING SCALE (PAS)** | *Park et al., 2024 | Cross-sectional | United Kingdom | *N:* 501  *Age:* Female= M: 71.6, SD: 6.41; Male= M: 72.2, SD: 6.89; Nonbinary=  78.0 (NA)  *Gender:* Female=366, Male= 134, Nonbinary=1 | *N° items: 8*  *Dimension(s):* unidimensional (total score)  *Response scale:* 6-point Likert scale (from “disagree strongly” to “agree strongly”) | **RELIABILITY***:*  - Satisfactory reliability (ω = 0.86; 95% CI = 0.83–0.89) | **STRUCTURAL VALIDITY:**   - ***Exploratory Factor Analysis***   - EFAs by extracting, respectively, from one to four factors (KMO = 0.88; Bartlett’s test: Chi Square66 = 511.77, p < .001). The unidimensional solution showed all the PAS items being loaded ≥0.41 by a single positive aging factor.   - ***Confirmatory Factor Analysis***   - The model accounting for 8 of the original 18 items was the best to represent the data. CFA was excellent (CFI = 0.99; RMSEA = 0.05 [0.02–0.08]; SRMR = 0.04).  **MEASUREMENT INVARIANCE:**  - No statistically significant differences between models nested for their age groups: metric and scalar invariance between young-old and older adults.  **CONSTRUCT VALIDITY**:   - ***Criterion validity***   *Spearman’s correlations:* PAS scores and cognitive functioning (*r*s = 0.21), general health (*r*s = 0.20), hedonic well-being (*r*s = 0.20), eudaimonic social well-being (*r*s = 0.13), and eudaimonic psychological well-being (*r*s = 0.25). |
| **PRACTICAL MEMORY CONCERNS SURVEY (PMCS)** | *Reese, Cherry, & Norris, 2004 | Cross-sectional | United States | Community-dwelling older adults  *N:* 154  *Age:* young adults: M = 22.2(20-28); middle-aged: (M = 49.7 (38-59); older adults: M = 71.1(61-83)  *Gender:* 74% f | *N° of items:* 7 open questions  *Dimension(s):* 4 dimensions (memory self-efficacy, memory management, memory remediation, and memory fears in adulthood).  *Response scale:* free-response question | **INTERNAL CONSISTENCY:**  - The inter-rater reliability was calculated to assess the level of agreement among the raters who created the pool of items. The inter-rater reliability was found to be 78.8%, indicating a substantial level of agreement between the raters. | NA |
| **SUBJECTIVE AGING PERCEPTION SCALE (SAPS)** | *de Gracia et al. 2004 | Cross-sectional | Spain | Community-dwelling OA and older residents  *N:* 153  *Age:* M = 70.35, SD = 4.55  *Gender:* 58.2% F | *N° of items:* 12  *Dimension(s):* 4 dimensions (physical self-concept, cognitive self-concept, subjective perception of time, subjective perception of social relations)  *Response scale:* 7-point Likert scale (from “totally disagree” to “totally agree”) | **INTERNAL CONSISTENCY:**  - All Cronbach’s α values exceeded 0.80, which is considered adequate. | **STRUCTURAL VALIDITY:**   - ***Exploratory Factor Analysis***   - The validity structure of the questionnaire was examined through a factor analysis of the main components with Varimax rotation, using Kaiser normalization. The analysis confirmed a four-factor structure, which accounted for 89.94% of the total variance.   - ***Confirmatory Factor Analysis***   - A CFA confirmed the 4-factors structure. Fit indices were as follows: χ^2^ = 63.35, df = 48, RMSR = 0.03, GFI = 0.93, AGFI = 0.89, NFI = 0.97, CFI = 0.99, RMSEA = 0.04.  **MEASUREMENT INVARIANCE**:  - A simultaneous factor analysis assessed the invariance of factor structure by context of residence (living in residence vs living independently). A factorial combination of four factors comprising the same items was obtained, but with a different factorial structure. The hypothesis of invariance was rejected.  **CONSTRUCT VALIDITY:**   - ***Convergent validity***   ***Correlations*:**  - Pearson correlation coefficients were calculated between the SAPS with PGCMS and RSES. The correlation coefficients between SAPS and both criteria scales were moderately high, with a correlation coefficient of r = 0.49 for SAPS and PGCMS, and r = 0.51 for SAPS and RSES. |

*Note*. * Original validation study. NA= Not assessed. *Population:* OA = Older adults; *Statistical indices:* AGFI = Adjusted Goodness of Fit Index; ASV = Average Shared Squared Variance; AVE = Average Variances Extracted; CFA = Confirmatory Factor Analysis; CITC = Corrected Item-Total Correlations; CI = Confidence Interval; CMIN/df = Chi-Square Minimum Discrepancy divided by Degrees of Freedom; CVI = Content Validity Index; CR = Composite Reliabilities; DIF = Differential Item Functioning; EFA = Exploratory Factor Analysis; GFI = Goodness of Fit Index; H = Homogeneity Test; ICVI = Item-Level Content Validity Index; ICC = Intra-Class Correlation; IFI = Incremental Fit Index; ITC = Item-Total Correlations; KMO = Kaiser-Meyer-Olkin; M = Mean; MSV = Maximum Shared Squared Variance; PCA = Principal Component Analysis; PAF = Principal Axis Factor Analysis; PCFI = Parsimony Comparative Fit Index; r = Pearson’s r; RMSEA = Root Mean Square Error of Approximation; RUMM = Rasch Unidimensional Measurement Models; SD = Standard Deviation; SMSR = Standardized Root Mean Square Residual; SRMS = Standardized Root Mean Square Residual; TLI = Tucker-Lewis Index; WINMIRA = Windows Mixed Rasch Model Analysis. *Questionnaires:* ADK = Alzheimer's Disease Knowledge; CES-D-R10 = Center for Epidemiologic Studies Depression Scale - Revised, 10 items; FAMHQ = Facts on Aging and Mental Health Quiz; GDS = Geriatric Depression Scale; HRQoL = Health-Related Quality of Life; MCS = Mental Component Summary; PCS = Physical Component Summary; PGC = Philadelphia Geriatric Center Morale Scale; RBMT = Rivermead Behavioural Memory Test; RSES = Rosenberg Self-Esteem Scale; SF-36 = Short Form Health Survey - 36 items; SPF-IL = Social Production Function Instrument for the Level of Well-being; SPWB = Scales of Psychological Well-Being; SWLS = Satisfaction With Life Scale; WHOQOL-BREF = World Health Organization Quality of Life – BREF.

**PART 4 - DESCRIPTION OF REVIEWED INSTRUMENTS**

***Instruments Assessing Generalized VoA***

*Beliefs about aging (and cognitive changes).* The Active Ageing Awareness Questionnaire (AAAQ; Bahuri et al., 2021) is a questionnaire comprising 14 items (e.g., “Having a hobby such as gardening, fishing or baking”) and two stand-alone questions. The questionnaire measures unidimensional active aging awareness by assessing participants’ opinions of factors that may contribute to active aging. It has been validated on a sample of older adults aged 50 and above to assess internal validity, multi-item scale reliability, factorial structure, and test-retest stability.

The Knowledge of Memory Aging Questionnaire (KMAQ; Cherry et al., 2000) is a questionnaire designed to measure the extent to which individuals possess accurate information and beliefs about age-related memory changes. The questionnaire consists of 28 bipolar items with a true-false response scale (e.g., “Frequent complaining about memory problems is an early sign of Alzheimer’s disease”). It includes two main dimensions: normal memory aging and pathological memory aging. It has been validated on a sample of older adults to assess convergent validity and internal consistency.

The Essentialist and (Non)essentialist Beliefs about Aging (NEBA; Weiss et al., 2016) instrument is a questionnaire assessing individuals’ beliefs regarding the fixed and inevitable versus the malleable and modifiable nature of aging. (Non)essentialist beliefs about aging are commonly measured using a brief 4-item or an extended 10-item scale, which includes two subscales: one assessing a fixed view of aging (e.g., “Aging is solely caused by genetic factors”) and the other assessing a malleable view of aging (e.g., “Age is just a number and does not say much about a person”) (Weiss et al., 2016). This scale has demonstrated good internal consistency.

The single-item measure of (Non)essentialist Beliefs about Aging ([N]EBA-SIS; Weiss et al., 2025) is a self-report measure assessing essentialist beliefs about aging (Weiss et al., 2016) with the following question: “What do you think: Is the way we age predetermined by genetics and immutable, or is it malleable and changeable?”. The scale ranges from -50 (“Aging is genetically predetermined and immutable”) to 50 (“Aging is malleable and changeable”). Higher values of this item reflect the belief that aging is inevitable and immutable, lower values indicate a belief in the malleability of aging, and 0 represents a mixed perspective.

*Older age stereotypes.* The Views on Aging Scale (VAS; Kornadt et al., 2011) is a multi-domain questionnaire that consists of 27 bipolar statements assessing stereotypic views of older persons across eight life domains: friends and acquaintances (“new friendships”), financial situation and money-related issues (“financial burden”), family and partnership (“loneliness”), leisure activities and social or civic commitment (“opportunities”), physical and mental fitness, health, and appearance (“sickness”), personality and way of living (“important matters”), religion and spirituality (“meaning”), and work and employment (“work”). This questionnaire has been validated on a sample of young adults and older individuals, and its factorial structure has been evaluated.

The Age-Based Rejection Sensitivity Questionnaire (RSQ-Age; Kang & Chasteen, 2009) assesses concerns about and expectations of rejection based on age. Respondents read and imagine 15 scenarios (e.g., “Imagine that you have worked many years in a particular profession. Recently, an opportunity arose for a few employees to receive additional training.”) and rate them on a 6-point Likert scale, ranging from “very unlikely” to “very likely.” This measure allows for the assessment of age discrimination while minimizing potential bias related to individual sensitivity to age discrimination behaviors. The validation study shows good internal consistency and one-year test-retest reliability, and the one-dimensional structural validity was assessed.

The Multidimensional Scale for the Assessment of the Salience of Age (MSASA; Kruse & Schmitt, 2006) measures the salience of age in social interactions, providing insights into age-related stereotypes or sets of beliefs. The scale comprises 24 items rated on a 4-point Likert scale ranging from “completely agree” to “completely disagree.” It encompasses five domains: developmental gains and chances for development (e.g., “Older people have more inner calmness than younger people”), developmental losses and risks of development (e.g., “Most older people feel lonely”), older people as a burden on society (e.g., “Older people are too expensive for public budgets”), social downgrading of older people (e.g., “The achievements of older people are not appreciated in our society”), and age salience (e.g., “What I expect from others depends on their age”). Validity and internal consistency have been assessed for this scale on a large sample of adults and older adults.

The Everyday Ageism Scale (EAS; Allen et al., 2022) is a questionnaire comprising 10 items that measure the prevalence of routine ageism individuals experience in their daily lives. In particular, it evaluates easily recognizable beliefs, experiences, and concrete behaviors that represent commonly encountered instances of everyday ageism without requiring respondents to identify them explicitly as such. The stem for these items was “How much do you agree with the following statements?” with a 4-point Likert response scale (from “strongly agree” to “strongly disagree”). It consists of three dimensions: the frequency of exposure to ageist messages (e.g., “I hear, see, and/or read things suggesting that older adults and aging are unattractive”), the frequency of ageism in interpersonal interactions (e.g., “Feeling lonely is part of getting older”), and the endorsement of internalized ageism (e.g., “People assume I do not do anything important or valuable”). The scale has been validated in a sample of older adults aged 50 to 80, with the validation process assessing internal consistency, structural validity, and known-group validity.

The Image of Aging Scale (IAS; Levy et al., 2004) asks participants to indicate whether 18 items match the images that come to mind when they think about older people in general (excluding themselves). Half of the items are positive (e.g., “capable”), and half are negative (e.g., “helpless”). Participants rate the extent of agreement on a scale ranging from 0 (“does not match my image of old people at all”) to 6 (“exactly matches my image of old people”). The scale has demonstrated good 1-week test-retest reliability, internal consistency, and convergent validity with older individuals.

The Relating to Old People Evaluation (ROPE; Cherry & Palmore, 2008) is a 20-item questionnaire that assesses two main dimensions of ageist behaviors exhibited by individuals in their everyday lives that is positive (e.g., “Enjoy conversations with old people because of their age”) and negative (e.g., “Avoid old people because they are cranky”). The response scale uses a 3-point Likert scale (“never,” “sometimes,” “often”). The validation process involved younger and older adults and examined test-retest reliability and internal consistency reliability.

The Ageism Survey (AS; Palmore, 2001) consists of 20 items that present a wide range of events related to ageism and asks participants to rate how often they have experienced each one. Examples of items include “I was called an insulting name related to my age” and “I was treated with less dignity and respect because of my age.” Response options included 0 (“never”), 1 (“once”), and 2 (“more than once”). The Ageism Survey has been shown to have high levels of reliability and validity, with all items loading onto a single factor.

The original Expectations Regarding Aging (ERA; Sarkisian et al., 2002) is used to assess individuals’ expectations of their physical and cognitive health as they age, and it consists of 38 items. The Persian Version of the 12-Item ARE (Nia et al. 2023) was evaluated in a sample of older adults aged 65 years and older. This version includes 12 items that measure expectations of aging across three factors: physical health (e.g., “The human body is like a car: when it gets old, it gets worn out”), mental health (e.g., “As people get older they worry more”), and cognitive function (e.g., “Forgetfulness is a natural occurrence just from growing old”). The study findings indicated three-factor construct validity and good internal consistency among the dimensions.

*Attitudes toward aging.* The Attitudes to Ageing Questionnaire (AAQ; Laidlaw et al., 2007) is an instrument developed by the WHOQOL Group to assess individuals’ opinions and feelings regarding events, issues, and experiences related to aging and older people. It consists of 24 items that are divided into three domains: psychosocial loss (e.g., “Old age is a time of loneliness”), physical change (e.g., “It is important to take exercise at any age”), and psychological growth (e.g., “Wisdom comes with age”). The questionnaire’s internal reliability and three-factor structure have been confirmed through exploratory and confirmatory factor analyses as well as the item response theory approach. Rejab et al. (2022) adapted the AAQ and confirmed the presence of the three-factor model and the instrument’s good internal consistency. A short version of the 12-item short form Attitudes to Aging Questionnaire (AAQ-SF; Laidlaw et al., 2018) has been developed. Confirmatory factor analysis has demonstrated that the AAQ-SF maintains an adequate fit with the three-factor structure of the original AAQ. Additionally, the AAQ-SF has shown construct-level measurement invariance across respondents who score above the cutoff.

***Instruments Assessing Personal VoA***

*Subjective age.* The measurement of felt age can vary across studies and commonly includes single-item measures, numerical rating scales, and multi-item scales. Single-item measures ask participants to indicate their perceived age relative to their actual age. Numerical rating scales allow participants to rate their felt age on a scale whereas multi-item scales consist of several statements assessing various aspects of felt age. Benyamini and Burns (2020) measured perceptions of age in four ways. These included age-group identity, in which participants identified themselves with specific age groups; comparative age, which involved comparing one’s age to another age group; perceived age, determined by the number of years participants felt compared to their chronological age (see also National Survey of Midlife Development in the United States); and subjective nearness to death, indicating the age to which participants believe they will live. These approaches to measuring felt age provide insights into individuals’ subjective experiences and perceptions of their aging process. Although these measures lack requirements for psychometric properties (see also Table 2 in the manuscript), Benyamini and Burns (2020) reported positive and significant correlations among these measures, supporting the age perception measures’ construct validity.

*Self-Pperceptions of aging (and cognitive changes).* The Aging Perceptions Questionnaire (APQ; Barker et al., 2007) is a 32-item instrument designed to assess how individuals perceive and evaluate their aging experiences. The APQ includes seven subscales: timeline chronic (e.g., “I am conscious of getting older all of the time”), timeline cyclical (e.g., “I go through cycles in which my experience of ageing gets better and worse”), consequences positive (e.g., “As I get older I get wiser”), consequences negative (e.g., “Getting older restricts the things that I can do”), control positive (e.g., “The quality of my social life in later years depends on me”), control negative (e.g., “Slowing down with age is not something I can control”), and emotional representations (e.g., “I get depressed when I think about how ageing might affect the things that I can do”). The APQ has demonstrated reliability and validity through good internal consistency of its subscales. The subscale interrelations are logically consistent, and there are associations between APQ subscales and indices of physical and psychological health that support this tool’s psychometric properties. The Brief Ageing Perceptions Questionnaire (BAPQ; Sexton et al., 2014) shows improved fit compared to the longer version and aligns with the original conceptual model. The brief version’s internal consistency reliability remains intact. Convergent and discriminant validity are also maintained.

The Subjective Aging Perception Scale (SAPS; de Gracia et al., 1999) comprises 12 items and measures various dimensions of self-concept and relevant aspects of well-being. The factorial validation study identified a four-factor structure: physical self-concept (e.g., “I think I am pretty fit for my age”), cognitive self-concept (e.g., “I think I have the same mental agility as before”), subjective perception of time (e.g., “I am so busy that there just aren’t enough hours in the day”), and subjective perception of social relations (e.g., “I think I have a good relationship with my friends”). Each factor consists of three items. Respondents rate each item on a 7-point Likert scale, ranging from 1 (“totally disagree”) to 7 (“totally agree”). Internal consistency was adequate, and the validity structure was assessed, confirming a four-dimensional structure. The convergent validity with self-esteem and psychological well-being was satisfactory among older adults (de Gracia et al., 1999).

The Anxiety about Aging Scale (AAS; Lasher & Faulkender, 1993) requires the participants to indicate on a 5-point Likert scale the extent to which they agree or disagree with 20 items that assess their overall anxiety about aging. This scale presented four subscales: fear of old people (e.g., “I enjoy being around old people”), psychological concerns (e.g., “I will have plenty to occupy my time when I am old”), physical appearance (e.g., “I have never lied about my age in order to appear younger”), and fear of loss (e.g., “I fear that when I am old all my friends will be gone”). The AAS showed high internal consistency in its scale with a Cronbach alpha of .82. Construct validity was established by demonstrating that the AAS scores are negatively correlated with the amount of contact, self-efficacy, and knowledge of aging and positively correlated with the quality of contact with the elderly (Lasher & Faulkender, 1993).

The Practical Memory Concerns Survey (PMCS; Reese et al., 1999) is a workbook comprising free-response questions (e.g., “Please list the five things that are the most difficult to remember in your day-to-day life”) measuring memory self-efficacy, memory management, memory remediation, and memory fears in adulthood. For each question, participants had to list five responses. The original version of the PMCS showed adequate internal consistency (Reese et al., 1999).

The Aging-Related Cognitions Scales (AgeCog; Steverink et al., 2001; Wurm et al., 2007) is a multidimensional scale that takes a domain-specific approach to assess the various positive and negative aspects of the aging experience separately. Participants rated the extent to which 4 statements reflected their views on aging using the prompt “Ageing means to me that...” The scale covers four domains: physical losses (“...I am less energetic and fit”), social losses (“...I feel less needed”), continuous growth and ongoing development (“...I can still learn new things”), and increased self-knowledge (“...I know myself better”). Each domain consists of four statements that are rated on a 4-point scale ranging from 1 (“strongly agree”) to 4 (“strongly disagree”). The scores for each domain are averaged, and higher values indicate either more negative aging cognitions (AgeCog Physical Losses and AgeCog Social Losses) or more positive aging cognitions (AgeCog Ongoing Development and AgeCog Self-Knowledge). Confirmatory factor analysis models confirmed the scales’ distinctiveness.

The Metamemory in Adulthood (MIA; Dixon, Hultsch & Hertzog, 1988) questionnaire assesses memory functioning and knowledge about memory processes. It consists of seven subscales: task (e.g., “For most people, facts that are interesting are easier to remember than facts that are not”), capacity (e.g., “I am good at remembering names”), change (e.g., “I can remember things as well as always”), anxiety (e.g., “I get upset when I cannot remember something”), achievement (e.g., “It is important to me to have a good memory”), locus (e.g., “I can’t expect to be good at remembering zip codes at my age”), and strategy (e.g., “Do you keep a list or otherwise note important dates, such as birthdays and anniversaries?”). The strategy subscale is further divided into external strategies and internal strategies. External strategies involve the use of memory aids, such as shopping lists and calendars, whereas internal strategies involve internal mnemonic techniques, such as visual imagery, forming associations, and mental rehearsal. Participants indicate the frequency with which they employ each strategy by rating items on a 5-point Likert scale ranging from 1 (“never”) to 5 (“always”). The validation of the questionnaire confirmed the seven-factor structure and demonstrated good internal consistency among the subscales.

The Memory Functioning Questionnaire (MFQ; Gilewski et al., 1988) is a 63-item questionnaire that measures memory functioning and is scored on a Likert scale ranging from 1 (indicating a “serious problem”) to 7 (indicating “never a problem”). The scale has undergone formal validation and includes several subscales based on factor analysis. These subscales include general frequency of forgetting, which assesses the types of memory problems experienced (e.g., “How would you rate your memory in terms of the kinds of problems that you have?”); seriousness of forgetting (“When you actually forget in these situations, how serious of a problem do you consider the failure to be?”), which measures how serious the individual considers memory failures to be in specific situations; retrospective functioning (e.g., “How is your memory compared to the way it was … 1 year ago?”), which compares current and past memory; and mnemonics usage (“How often do you use these techniques to remind yourself about things?”), which evaluates the frequency of using techniques to remind oneself about things. The validation study confirmed a four-factor structure and demonstrated good stability of the questionnaire.

The Memory Controllability Inventory (MCI; Lachman et al., 1995) is a 19-item questionnaire designed to assess beliefs and perceptions about memory control. The MCI consists of six subscales: present ability (e.g., “I can remember the things I need to”), potential improvement (e.g., “I can remember the things I need to”), effort utility (e.g., “If I work at it, I can improve my memory”), inevitable decrement (e.g., “There’s not much I can do to keep my memory from going downhill”), independence (e.g., “As I get older I won’t have to rely on others to remember things for me”), and Alzheimer’s likelihood (e.g., “I sometimes think that 1 have Alzheimer’s disease”). Each subscale captures a specific aspect related to memory control and beliefs about memory abilities. For example, the present-ability subscale assesses individuals’ beliefs about their current memory performance, and the potential-improvement subscale evaluates their confidence in their ability to enhance their memory. Response options for all items range from 1 (“strongly disagree”) to 7 (“strongly agree”). The validation of the MCI demonstrated a six-factor structure, supporting the questionnaire’s subscales. Additionally, the predicted correlations among the subscales provide further evidence of the scale’s validity.

The Positive Aging Scale (PAS; Park et al., 2024) is an 8-item questionnaire that assesses positive aspects of aging through meaningful everyday experiences, group memberships, personal resources, and extrinsic factors. Participants indicate their level of agreement on a 6-point Likert scale ranging from 1 (“strongly disagree”) to 6 (“strongly agree”). The validation study (Park et al., 2024) demonstrated good internal consistency, a unidimensional structure, and no measurement invariance across age groups (young-old and older adults). It also showed good criterion validity with general health, hedonic well-being, eudaimonic social well-being, and eudaimonic psychological well-being. A higher total score reflects more positive perceptions of aging.

*Awareness of age-related change.* The Awareness of Age-Related Change questionnaire (AARC-50; Brothers et al. 2019) consists of 25 items assessing AARC-Gains and 25 items assessing AARC-Losses in the five behavioral domains, namely health and physical functioning, cognitive functioning interpersonal relations, and social-cognitive and social-emotional functioning. Each item begins with the phrase “With my increasing age, I realize that...” followed by either gain-related (e.g., “...I pay more attention to my health”) or loss-related (e.g., “...my ability to move around has gotten worse”) experiences. Participants indicate their level of agreement on a 5-point Likert scale ranging from 1 (“not at all”) to 5 (“very much”). The validation study initially assessed the structure’s validity using the complete version, which comprises 189 items, and later evaluated the measurement properties of the current 50-item version. The study confirmed good psychometric properties, including structure validity, good item and scale reliability, and adequate convergent validity. A short version of the questionnaire, called the 10-item Awareness of Age-Related Change questionnaire (AARC-10; Kaspar et al., 2019), has also been developed. This questionnaire showed adequate fit to the data and reliability for the perceived gain and loss composites. Concurrent and discriminant validity were confirmed.

*Attitudes toward one’s own aging.* The Attitudes Toward Own Aging Scale (ATOA) was originally developed as a 5-item subscale of the Philadelphia Geriatric Center Morale Scale (PGCMS; Lawton [1975](https://link.springer.com/article/10.1007/s10804-018-9283-3#ref-CR18)). Participants indicated their degree of agreement with 5 items (e.g., “Things keep getting worse as I get older”). The original version has a dichotomous response (“Yes” or “No”) response scale. The validation study confirmed that this subscale showed satisfactory internal consistency.
